# Pd-catalysed ligand-enabled carboxylate-directed highly regioselective arylation of aliphatic acids

**DOI:** 10.1038/ncomms14904

**Published:** 2017-04-06

**Authors:** Yan Zhu, Xiaolan Chen, Chunchen Yuan, Guobao Li, Jingyu Zhang, Yingsheng Zhao

**Affiliations:** 1 Key Laboratory of Organic Synthesis of Jiangsu Province, College of Chemistry, Chemical Engineering and Materials Science, Soochow University, Suzhou 215123, China

## Abstract

α-amino acids bearing aromatic side chains are important synthetic units in the synthesis of peptides and natural products. Although various β-C-H arylation methodologies for amino acid derivatives involving the assistance of directing groups have been extensively developed, syntheses that directly employ *N*-protected amino acids as starting materials remain rare. Herein, we report an *N*-acetylglycine-enabled Pd-catalysed carboxylate-directed β-C(*sp*^3^)-H arylation of aliphatic acids. In this way, various non-natural amino acids can be directly prepared from phthaloylalanine in one step in good to excellent yields. Furthermore, a series of aliphatic acids have been shown to be amenable to this transformation, affording β-arylated propionic acid derivatives in moderate to good yields. More importantly, this ligand-enabled direct β-C(*sp*^3^)-H arylation could be easily scaled-up to 10 g under reflux conditions, highlighting the potential utility of this synthetic method.

## Abstract (web summary)

Palladium catalysed C-H functionalization of *sp* ^3^ carbons typically requires the installation and subsequent removal of a temporary directing group. Here the authors report a method allowing C-H functionalization in carboxylic acids in which the carboxylate acts as a directing group.

Aliphatic acids are highly important synthetic units, being commonly found in natural products, approved drugs and biologically important molecules^1^,^2^. Direct C–H functionalization of C(*sp*^3^)–H bonds in aliphatic acids is highly important and attractive, because it provides a straightforward pathway to afford valuable chemicals with higher atom-economy compared to traditional procedures, for which pre-functionalized substrates are needed^3^,^4^,^5^,^6^,^7^,^8^,^9^,^10^. During the last decades, tremendous progress has been made in terms of transition-metal-catalysed functionalization of C–H bonds. Although there have been exciting developments in carboxylate-assisted functionalization of arene C–H bonds^11^,^12^,^13^,^14^,^15^, reports of the direct functionalization of β-C(*sp*^3^)–H bonds remain limited. Only the specific structure of a carboxylic acid facilitates direct β-C(*sp*^3^)-H functionalization in the presence of a palladium catalyst, as disclosed by Yu *et al*. in 2007 (Fig. 1a)^16^. However, the substrate scope was limited to aliphatic acids with a β-quaternary centre, due to the well-known Thorpe–Ingold effect in cyclopalladation^17^, and ratios of mono- to diarylated products in the range 2.5:1 to 5:1 were observed. Thus, the use of various directing groups to achieve regioselective β-C(*sp*^3^)–H bond functionalization of carboxylic acid derivatives^18^,^19^,^20^,^21^,^22^,^23^,^24^,^25^,^26^,^27^,^28^,^29^,^30^,^31^,^32^,^33^,^34^,^35^,^36^,^37^,^38^,^39^,^40^,^41^,^42^,^43^,^44^,^45^ has been extensively explored in the past decades (Fig. 1).

In 2005, Yu *et al*. reported early pioneering work by employing an oxazoline ring as a mono-coordinated directing group to realize β-C(*sp*^3^)-H iodination^18^,^19^. In the same year, Daugulis reported the well-known bidentate directing group 8-aminoquinoline, which has been demonstrated to have excellent ability in assisting β-C(*sp*^3^)-H arylation of carboxylic acids^20^. Since then, a number of β-C(*sp*^3^)-H functionalization reactions, such as arylation^21^,^22^,^23^, alkenylation^24^, alkylation^25^,^26^, alkynylation^27^,^28^, alkoxylation^29^, amination^30^ and so on^31^,^32^,^33^,^34^,^35^, employing various transition metal catalysts, have been developed. Inspired by the assisting ability of 8-aminoquinoline, researchers developed a series of *N*,*N*-bidentate directing groups for site-selective C(*sp*^3^)–H bond functionalization in aliphatic acids, which provided various approaches in the synthesis of important synthetic units (Fig. 1d)^20^. For example, the 2-(pyridin-2-yl)isopropyl directing group was successfully applied in β-C(*sp*^3^)-H arylation, acetoxylation and intramolecular amination with a palladium catalyst by Shi *et al*.^37^

A bidentate α-amino oxazolinyl directing group was developed for site-selective arylation of secondary C(*sp*^3^)–H bonds by Shi's group in 2015 (ref. 38). In recent years, *N*,*O*-bidentate directing groups have been identified as being effective in directing site-selective C–H arylation of carboxylic acids in the presence of a palladium catalyst, and have been developed by the groups of Chatani^39^, Yu^40^,^41^, Hong^42^ and Zhao^43^. Yu's group also demonstrated that mono-coordinated directing groups, such as perfluorinated aryl amides and *N*-methoxyamide, can effectively direct β-C(*sp*^3^)-H arylation with the assistance of mono-*N*-protected amino acid ligands (Fig. 1c)^44^,^45^,^46^,^47^. For example, *N*-methoxyamide has been used as a directing group^48^,^49^ for β-C(*sp*^3^)-H arylation with a pyridine-type ligand, affording various non-natural amino acids from protected alanine derivatives^49^. These developments have greatly expanded the possible approaches to construct key structures of natural products, pharmaceutical agents and organic materials.

Although the directing group strategy^50^,^51^,^52^,^53^ has been successfully deployed in the past decades, an inevitable drawback is the need for additional synthetic steps for installation and removal of the directing group. Thus, an ideal approach to functionalize an aliphatic acid would directly employ the carboxyl group itself as an auxiliary. However, there is still no efficient general approach for the direct functionalization of aliphatic acids that does not require the installation of a directing group. α-Amino acids bearing aromatic side chains are important synthetic units in the synthesis of peptides and natural products. To date, various β-C-H arylation protocols for amino acid derivatives involving the assistance of directing groups have been extensively developed^37^,^43^,^49^,^54^. However, the most straightforward way to synthesize these amino acid derivatives would be to directly employ the *N*-protected amino acid as the starting material. Inspired by recent developments in ligand-accelerated or ligand-enabled C–H activation reactions^54^,^55^,^56^, we speculated that carboxylate-assisted β-C(*sp*^3^)-H activation might be achieved by employing a congruous ligand. Thus, we investigated whether synthetically important phenylalanine derivatives^14^,^54^,^57^,^58^,^59^,^60^,^61^ could be directly synthesized from phthaloylalanine through arylation of the β-C(*sp*^3^)–H bond with a carboxyl group as a coordination centre and the assistance of a ligand.

Herein, we report our discovery that the carboxyl group itself can act as a coordination centre by combination with a mono-protected amino acid ligand to realize β-C(*sp*^3^)-H activation (Fig. 1e). This is the first example of a general, palladium-catalysed, highly site-selective arylation of β-C(*sp*^3^)–H bonds of various aliphatic acids. Various aryl iodides are tolerated, directly affording a large number of non-natural amino acids from phthaloylalanine in moderate to excellent yields. A series of aliphatic acids have also been transformed, highlighting the potential utility of this synthetic method.

## Results

### Optimization of reaction conditions

At the beginning of our study, we first treated phthaloylalanine (**1a**) with 4-iodoanisole in the presence of Pd(OAc)_2_ (5 mol%), Ag_2_CO_3_ (1 equiv), K_2_CO_3_ (0.5 equiv) and (*n*-BuO)_2_PO_2_H (0.2 equiv) in hexafluoroisopropanol (HFIP) at 100 °C for 24 h. Surprisingly, the desired product **3a** was obtained in 8% yield along with 86% of recovered **1a** (Table 1, entry 1). Encouraged by this result, we screened various additives, ligands and solvents (see Supplementary Information, Supplementary Tables 4–9). The results revealed that the phosphine and *N*-coordinated ligand could not give the arylated **3a** in yields higher than 10%. When we tested some well-known mono-protected amino acid ligands, these displayed effective promoting effects on this arylation of β-C(*sp*^3^)–H bonds (entries 6–11). After screening various mono-protected amino acid ligands, it was found that the simple Ac-Gly-OH afforded the phenylalanine derivative **3a** in 86% yield (entry 9). Interestingly, the additives (*n*-BuO)_2_PO_2_H and 1-AdCO_2_H showed no promoting effect when Ac-Gly-OH was employed as the ligand. Further investigations revealed that Ag_2_CO_3_ was indispensable and could not be replaced (entries 14–16).

### Substrate scope of aryl iodides

**Figure 2:**
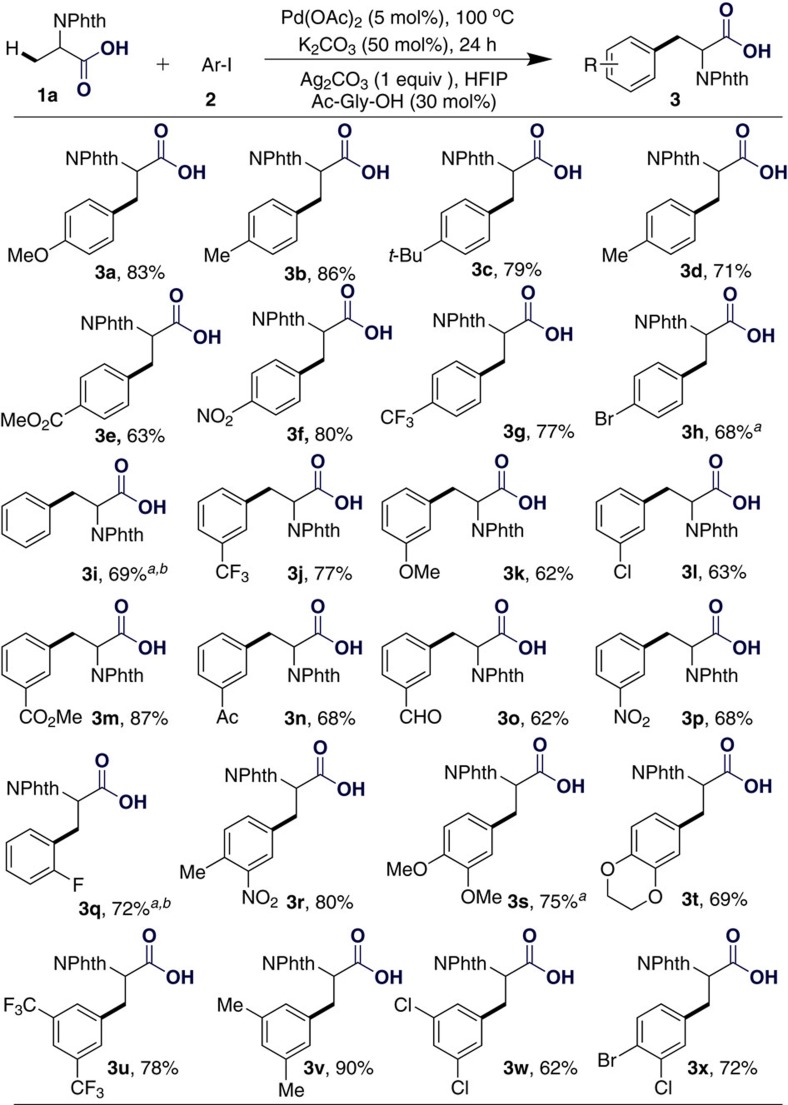
Substrate scope of aryl iodides. Reaction conditions: **1a** (0.2 mmol), **2** (0.3 mmol), Pd(OAc)_2_ (5 mol%), Ag_2_CO_3_ (0.2 mmol), K_2_CO_3_ (0.1 mmol), Ac-Gly-OH (0.06 mmol), HFIP (2 ml), 100 °C, 24 h. Isolated yields. ^a^Pd(OAc)_2_ (10 mol%). ^b^110 °C.

With the optimized conditions in hand, phthaloylalanine (**1a**) was treated with a wide range of aryl iodides to form important non-natural amino acids that are widely applied in the preparation of bioactive peptides. Aryl iodides bearing *para* or *meta* substituents afforded the corresponding products in good to excellent yields (see Fig. 2). Various functional groups, such as MeO, Me, *t-*Bu, F, Cl, Br and CF_3_, were well tolerated in this transformation. It is worthy of mention that the functional groups Ac, CO_2_Me, CHO and NO_2_ were also compatible with this transformation, and the corresponding phenylalanine derivatives were obtained in good yields. An *ortho*-substituted aryl iodide was also tolerated in this transformation by slightly modifying the conditions (**3q**). Multiply-substituted aryl iodides performed well, affording the corresponding non-natural amino acids in good yields (**3r**–**x**). It is worth mentioning that the bromo functional group in **3h** and **3x** could be further applied in peptide synthesis under Davis conditions^62^.

### Substrate scope of carboxylic acids and amino acids

**Figure 3:**
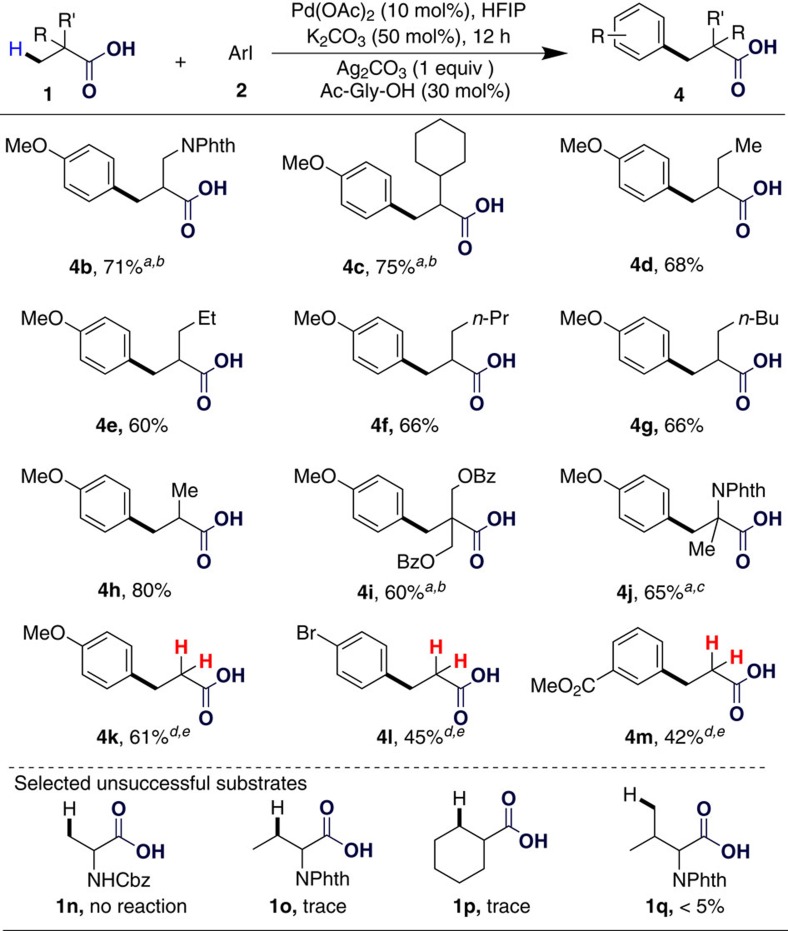
Substrate scope of carboxylic acids and amino acids. Reaction conditions: 0.2 mmol scale, Pd(OAc)_2_ (10 mol%), Ag_2_CO_3_ (0.2 mmol), K_2_CO_3_ (0.1 mmol), Ac-Gly-OH (0.06 mmol), HFIP (2 ml), 80 °C, 12 h. Isolated yields. ^a^100 °C. ^b^24 h. ^c^HFIP (4 ml). ^d^60 h. ^e^150 °C.

To demonstrate the generality of this carboxylate-directed C(*sp*^3^)-H arylation reaction, we next investigated its scope in terms of aliphatic acids under the optimal conditions (see Fig. 3). The phthaloyl-protected β-amino acid **1b** and 2-cyclohexylpropanoic acid **1c** performed well with 4-iodoanisole, affording the corresponding arylated products in good yields (**4b**,**4c**). The simple carboxylic acids (**1d**–**g**) also performed well when two equivalents thereof were treated with one equivalent of 4-iodoanisole at 80 °C for 12 h (**4d**–**g**). Benzoyl-protected 2,2-bis(hydroxymethyl)propionic acid was also compatible with this transformation and an acceptable yield of **4i** was obtained. Isobutyric acid (**1h**) and phthaloyl-protected 2-aminoisobutyric acid (**1j**) both afforded the mono-arylated products in good yields when two equivalents thereof were treated with one equivalent of 4-iodoanisole. The diarylation product of **1j** could also be obtained by treating it with three equivalents of 4-iodoanisole (see Supplementary Information, Supplementary Fig. 47). Direct functionalization of propionic acid is extremely challenging, due to the lack of a steric effect during its cyclopalladation. To our great delight, however, propionic acid also reacted well with 10 mol% palladium acetate at 150 °C for 60 h, affording various β-aryl-propionic acids in synthetically acceptable yields (**4k–m**). All the results indicated the excellent promoting ability of Ac-Gly-OH in this carboxylic-acid-assisted C(*sp*^3^)-H arylation. It is worth mentioning that when 4-iodoanisole was treated with six equivalents of pivalic acid in the presence of Pd(OAc)_2_ (10 mol%), Ag_2_CO_3_ (1 equiv), K_2_CO_3_ (0.5 equiv) and Ac-Gly-OH (0.3 equiv) in HFIP at 80 °C for 12 h, the monoarylated product was obtained in 68% yield and the diarylated product was obtained in less than 5% yield (see Supplementary Fig. 46). However, when carbobenzyloxy-protected alanine (**1n**) was tested, it failed to give any product and the starting material was recovered. Unfortunately, methylene C(*sp*^3^)–H bonds were not tolerated in this ligand-enabled carboxylate-assisted C–H transformation (**1o**,**1p**). Phthaloyl-protected valine (**1q**) was also treated with 4-iodoanisole at 150 °C for 60 h. The desired arylated product was only observed in trace amount, along with near-complete recovery of **1q**.

### Synthetic potential

**Figure 4:**
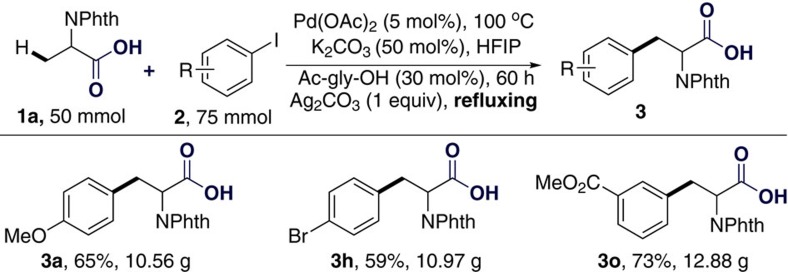
Ten gram scale reaction in one step. Arylation of *N*-phthaloyl-phenylalanine (**1a**) on a 10 g scale.

Considering the importance of the generated non-natural amino acids, if this newly developed carboxylate-assisted directed C(*sp*^3^)-H arylation could be performed on a 10 g scale, it would provide a very attractive and convenient approach for the synthesis of phenylalanine derivatives in the laboratory. To our delight, 10 g scale reactions proceeded well with a range of aryl iodides (Fig. 4, **3a**, 10.56 g; **3h**, 10.97 g; **3o**, 12.88 g) by employing a slightly longer reaction time, even under reflux conditions.

**Figure 5:**
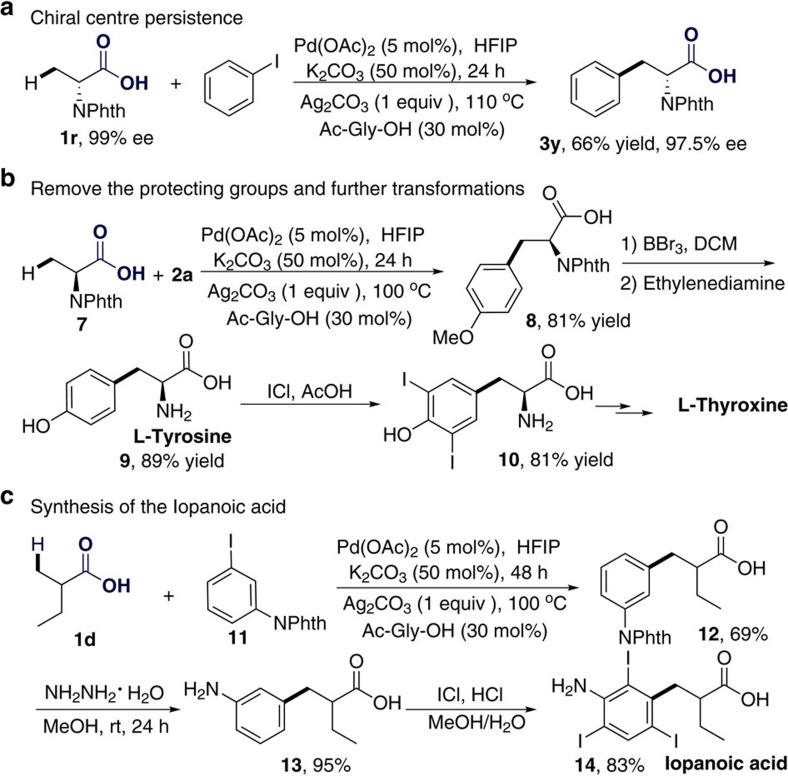
Synthesis of biologically active compounds. (**a**) Arylation of chiral substrate **1r**. (**b**) Remove the protecting groups and further transformations. (**c**) Synthesis of iopanoic acid.

When the chiral substrate **1r** was tested under the standard reaction conditions, the product **3y** was obtained in 66% yield. There was no significant racemization at the chiral centre, as determined by high-performance liquid chromatography (Fig. 5a). The methoxyl and phthalimide groups were easily removed under the reported conditions^49^,^63^, affording L-tyrosine in an overall yield of 84%. The L-tyrosine could be easily transformed into 3,5-diodo-L-tyrosine (**10**) in 81% yield, which is a key intermediate in the synthesis of L-thyroxine^64^ (Fig. 5b). Iopanoic acid, an iodine-containing radiocontrast medium used in cholecystography, could be easily prepared from the simple carboxylic acid **1d** in three steps in an overall yield of 47% (ref. 65) (Fig. 5c), highlighting the synthetic importance of the newly developed method^66^,^67^.

## Discussion

In conclusion, we have developed an efficient route for the palladium-catalysed direct β-C(*sp*^3^)-H arylation of carboxylic acids using the carboxyl group itself as a coordination centre. In this way, various non-natural amino acids have been directly prepared from phthaloylalanine in good to excellent yields. A series of aliphatic acids, including the challenging propionic acid and amino acids, have been shown to be compatible with this transformation, highlighting its potential synthetic utility. The important compounds 3,5-diodo-L-tyrosine and iopanoic acid have been readily prepared from simple carboxylic acids in good yields. This discovery of ligand-enabled carboxyl-promoted C(*sp*^3^)–H bond activation offers a convenient approach for the functionalization of C(*sp*^3^)–H bonds in aliphatic acids.

## Methods

### Typical procedure for Pd-catalysed C-H arylation of aliphatic acids

To a 15 ml sealed glass vial was added phthaloylalanine (**1a**, 0.2 mmol, 43.8 mg), 4-iodoanisole (0.2 mmol, 70.2 mg), Pd(OAc)_2_ (5 mol%, 4.5 mg), Ag_2_CO_3_ (0.2 mmol, 55.2 mg, 1 equiv), K_2_CO_3_ (0.1 mmol, 13.8 mg, 0.5 equiv), Ac-Gly-OH (0.06 mmol, 7.0 mg, 0.3 equiv). The tube was then sealed and the reaction mixture was stirred at 100 °C for 24 h. The reaction mixture was cooled to room temperature, and diluted with ethyl acetate and filtered through celite. The filtrate was concentrated in vacuo and purified by column chromatography on silica gel to provide the desired arylated product **3a** as a white solid (54.0 mg; yield 83%).

### Data availability

The authors declare that all relevant data supporting the findings of this study are available within the article and its Supplementary Information files.

## Additional information

**How to cite this article:** Zhu, Y. *et al*. Pd-catalysed ligand-enabled carboxylate-directed highly regioselective arylation of aliphatic acids. *Nat. Commun.* **8,** 14904 doi: 10.1038/ncomms14904 (2017).

**Publisher's note**: Springer Nature remains neutral with regard to jurisdictional claims in published maps and institutional affiliations.

## Supplementary Material

Supplementary Information Supplementary figures, supplementary tables, supplementary methods and supplementary references.

## Figures and Tables

**Figure 1:**
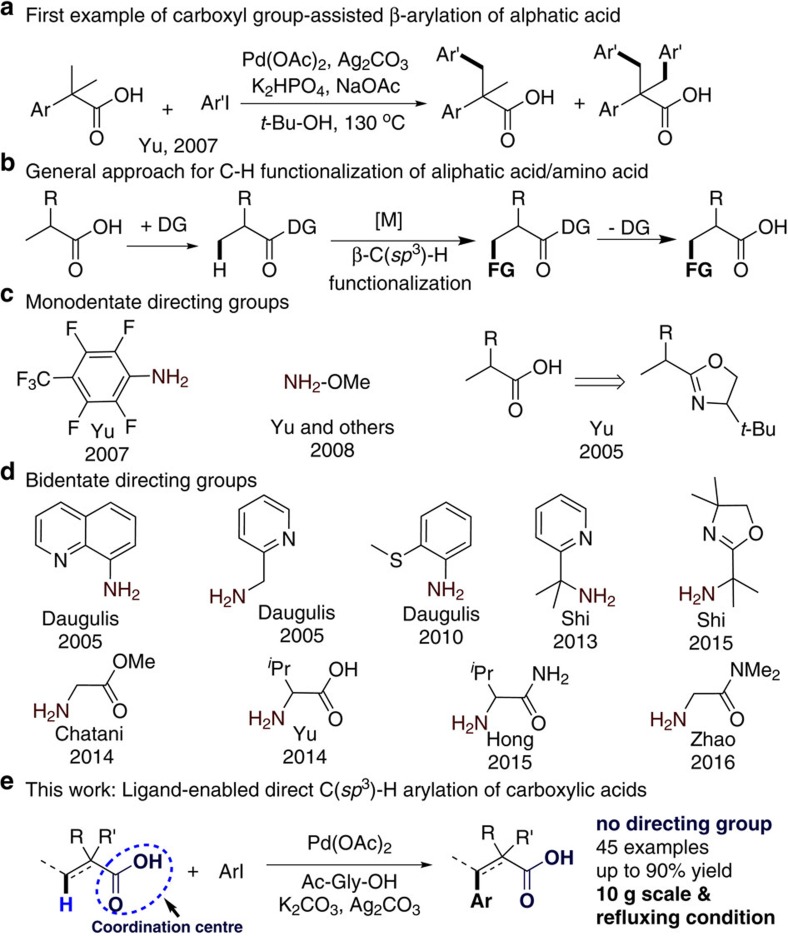
Directing group strategies for C–H activation. (**a**) First example of carboxyl-group-assisted β-arylation of an aliphatic acid. (**b**) General approach for C–H functionalization of aliphatic acids/amino acids. (**c**) Monodentate directing groups for C–H arylation. (**d**) Bidentate directing groups for C–H arylation. (**e**) Our work on ligand-enabled directed C(*sp*^3^)-H arylation of carboxylic acids.

**Table 1:** Optimization for direct C(*sp* ^3^)–H bond activation.

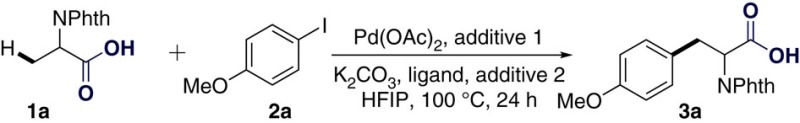					
**entry**	**ligand**	**additive 1**	**additive 2**	**recovery of 1a (%)**	**yield of 3a (%)**
1	none	Ag_2_CO_3_	(*n*-BuO)_2_PO_2_H	86	8
2	PPh_3_	Ag_2_CO_3_	(*n*-BuO)_2_PO_2_H	80	10
3	dppf	Ag_2_CO_3_	(*n*-BuO)_2_PO_2_H	93	Trace
4	Pyridine	Ag_2_CO_3_	(*n*-BuO)_2_PO_2_H	96	0
5	1,10-phen	Ag_2_CO_3_	(*n*-BuO)_2_PO_2_H	94	Trace
6	Ac-Leu-OH	Ag_2_CO_3_	None	24	65
7	Ac-Val-OH	Ag_2_CO_3_	None	18	70
8	Ac-isoLeu-OH	Ag_2_CO_3_	None	40	54
9	Ac-Gly-OH	Ag_2_CO_3_	None	5	86
10	Boc-Gly-OH	Ag_2_CO_3_	None	89	5
11	Cbz-Gly-OH	Ag_2_CO_3_	None	92	4
12	Ac-Gly-OH	Ag_2_CO_3_	(*n*-BuO)_2_PO_2_H	48	40
13	Ac-Gly-OH	Ag_2_CO_3_	1-AdCO_2_H	65	26
14	Ac-Gly-OH	Cu(OAc)_2_	None	88	5
15	Ac-Gly-OH	BQ	None	90	Trace
16	Ac-Gly-OH	Cu(OAc)_2_/O_2_	None	92	Trace

Reaction conditions: **1a** (0.1 mmol), **2a** (0.15 mmol), Pd(OAc)_2_ (5 mol%), additive 1 (0.1 mmol), K_2_CO_3_ (0.05 mmol), ligand (0.03 mmol), additive 2 (0.02 mmol), HFIP (1 ml), 100 °C, 24 h. Yields were based on LC-MS analysis using acetyl benzene as an internal standard.
