# Supplementary material for: Pd-catalysed ligand-enabled carboxylate-directed highly regioselective arylation of aliphatic acids
Source: Nat Commun. 2017 Apr 6;8:14904. doi: 10.1038/ncomms14904 (PMC5384235; doi:10.1038/ncomms14904)
Supplement: Supplementary Information — Supplementary figures, supplementary tables, supplementary methods and supplementary references. [file ncomms14904-s1.pdf]

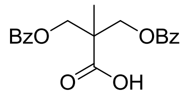

1i

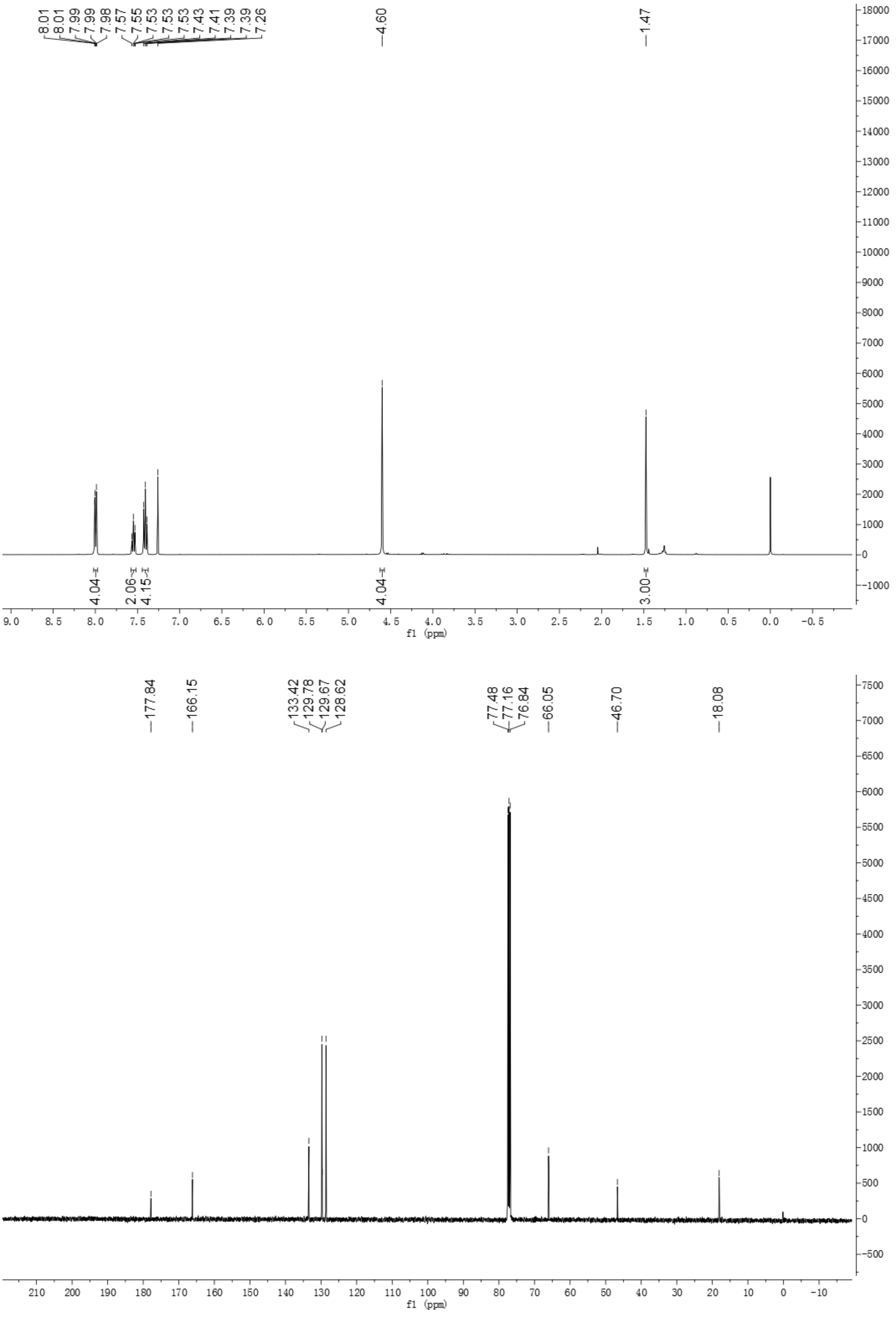

Supplementary Figure 1. ^{1}**H and** ^{13}**C NMR spectra for 1i**

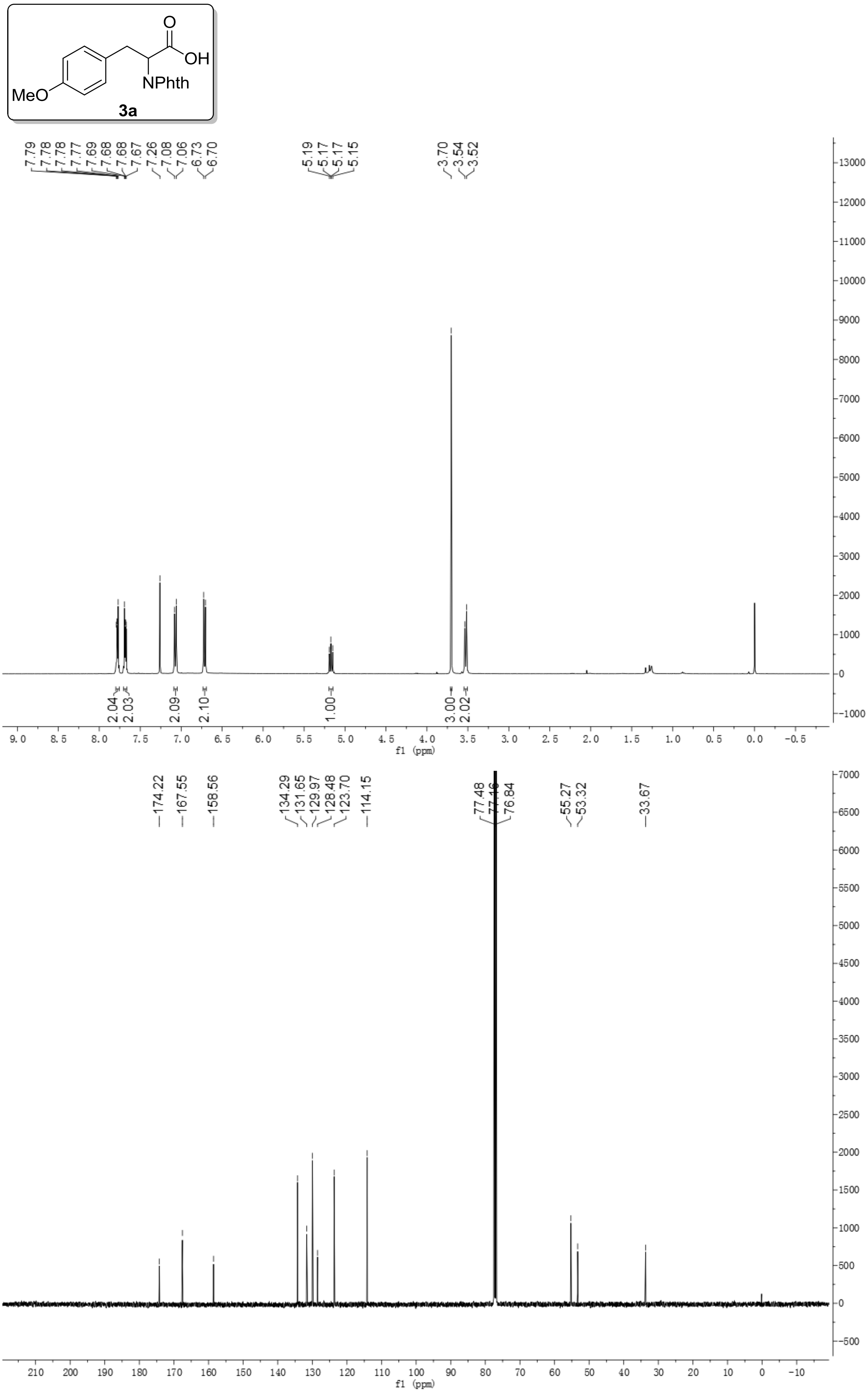

Supplementary Figure 2. ^{1}H and ^{13}C NMR spectra for 3a

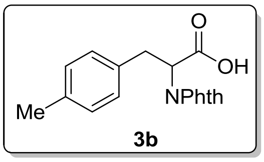

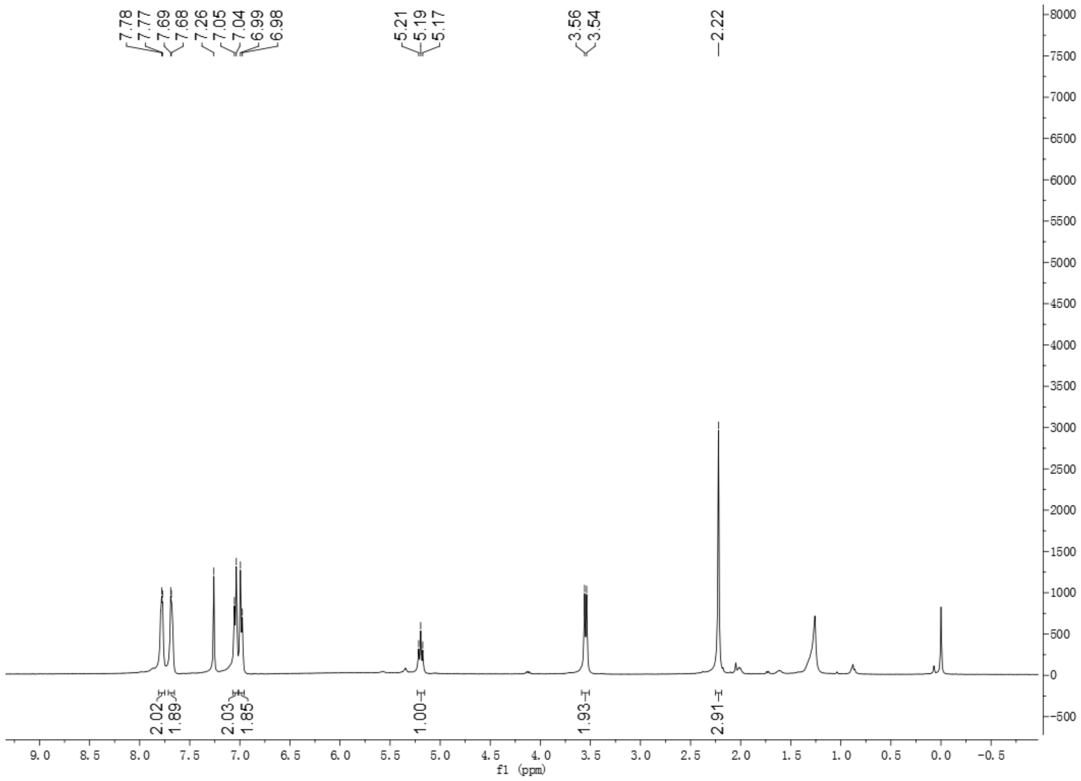

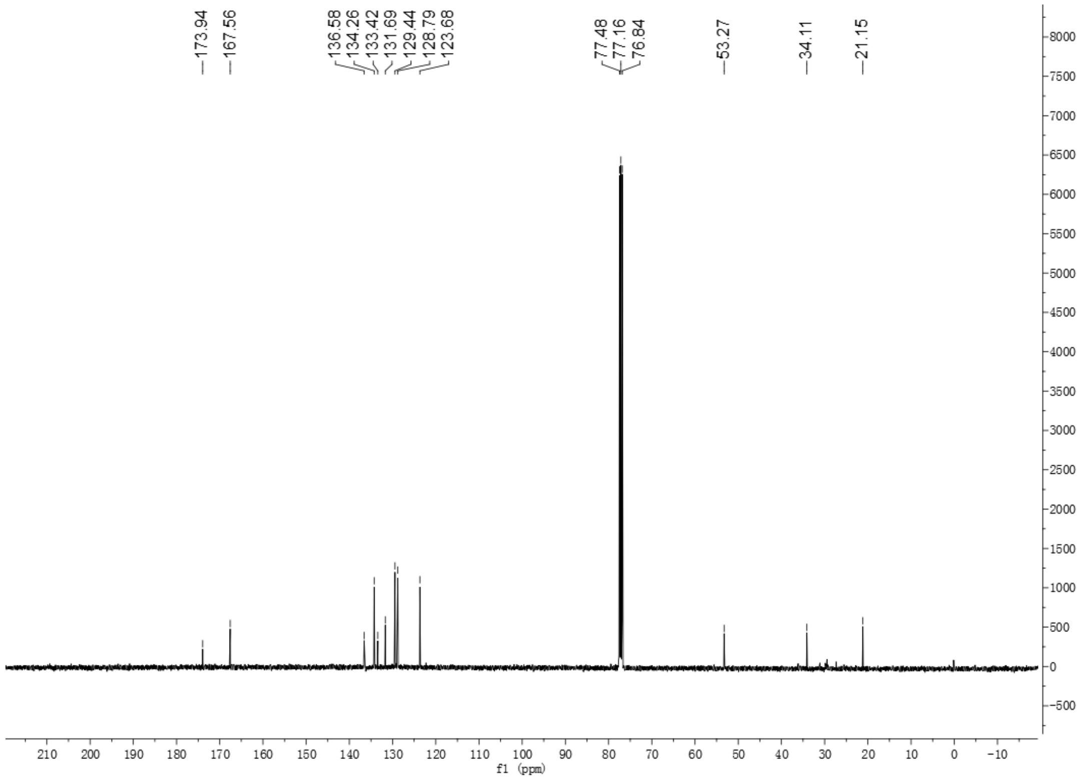

Supplementary Figure 3. ^{1}H and ^{13}C NMR spectra for 3b

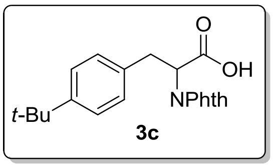

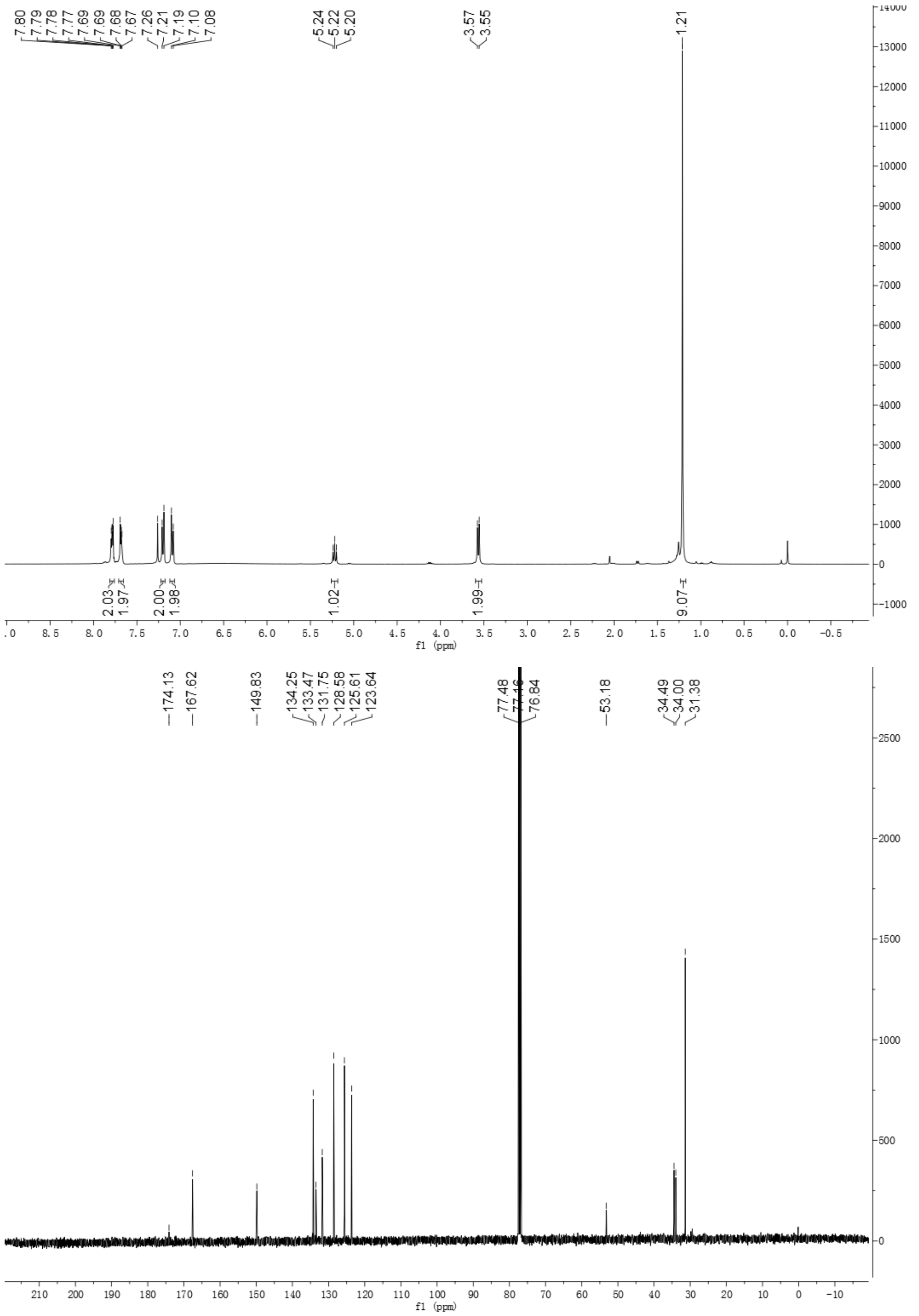

Supplementary Figure 4. ^{1}H and ^{13}C NMR spectra for 3c

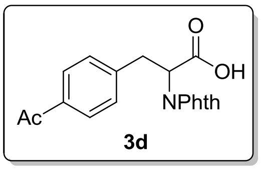

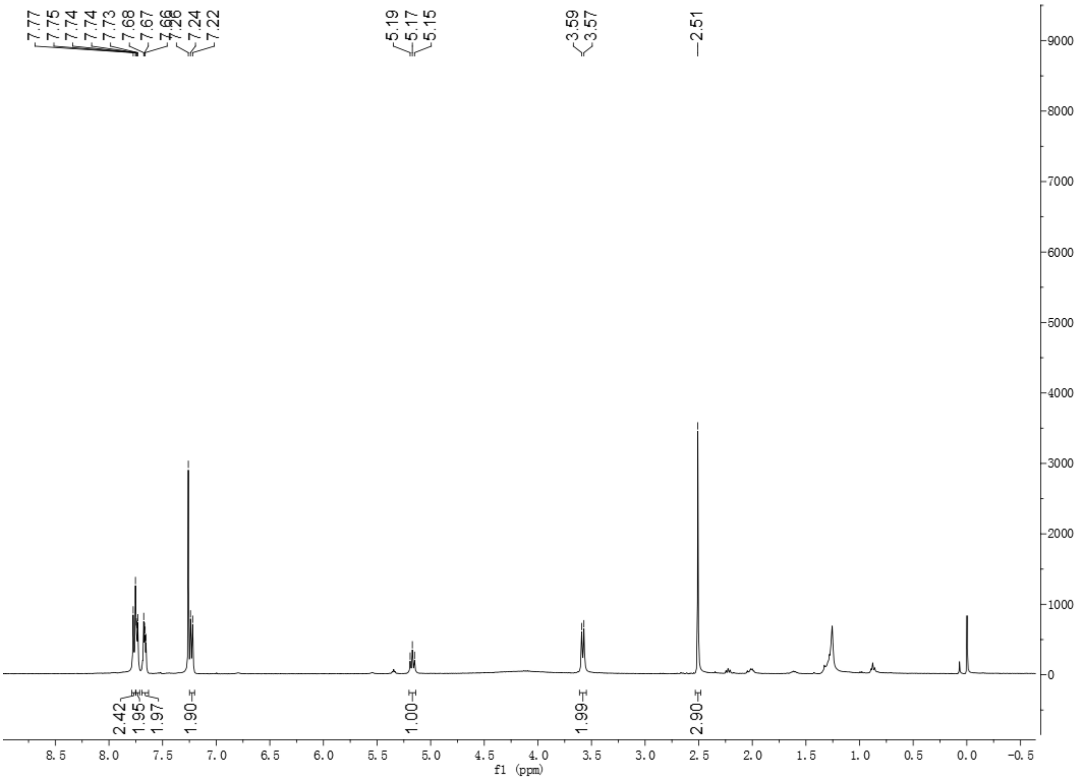

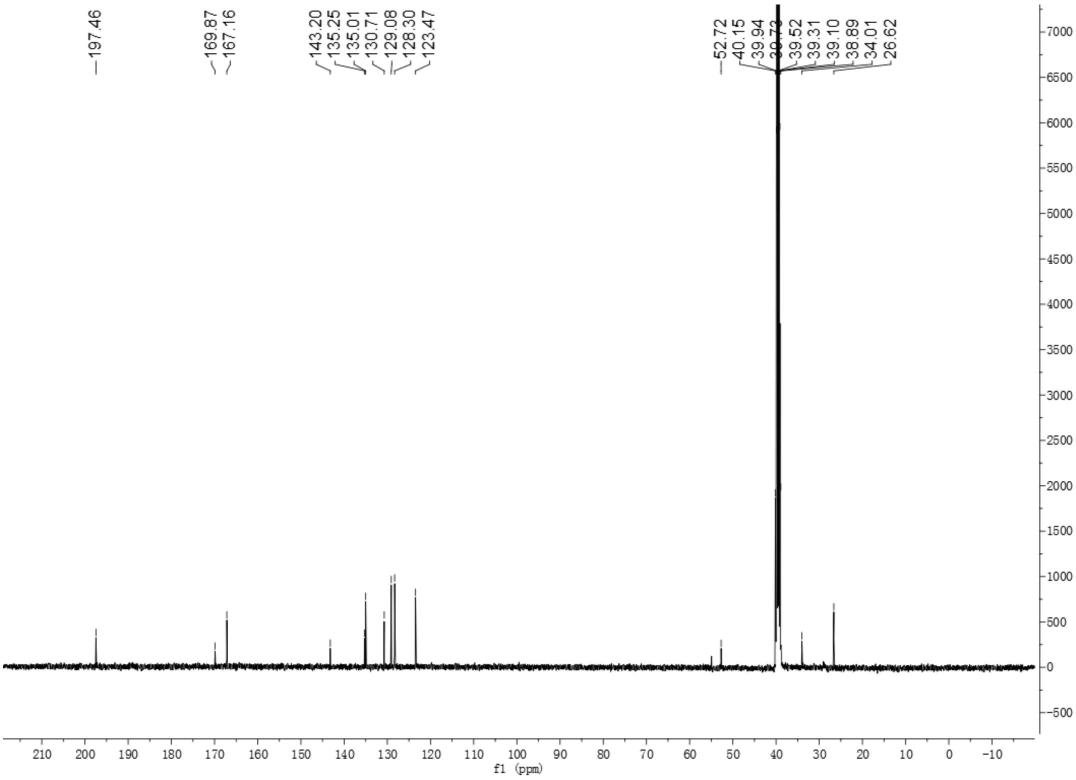

Supplementary Figure 5. ^{1}H and ^{13}C NMR spectra for 3d

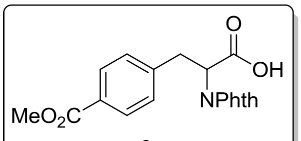

3e

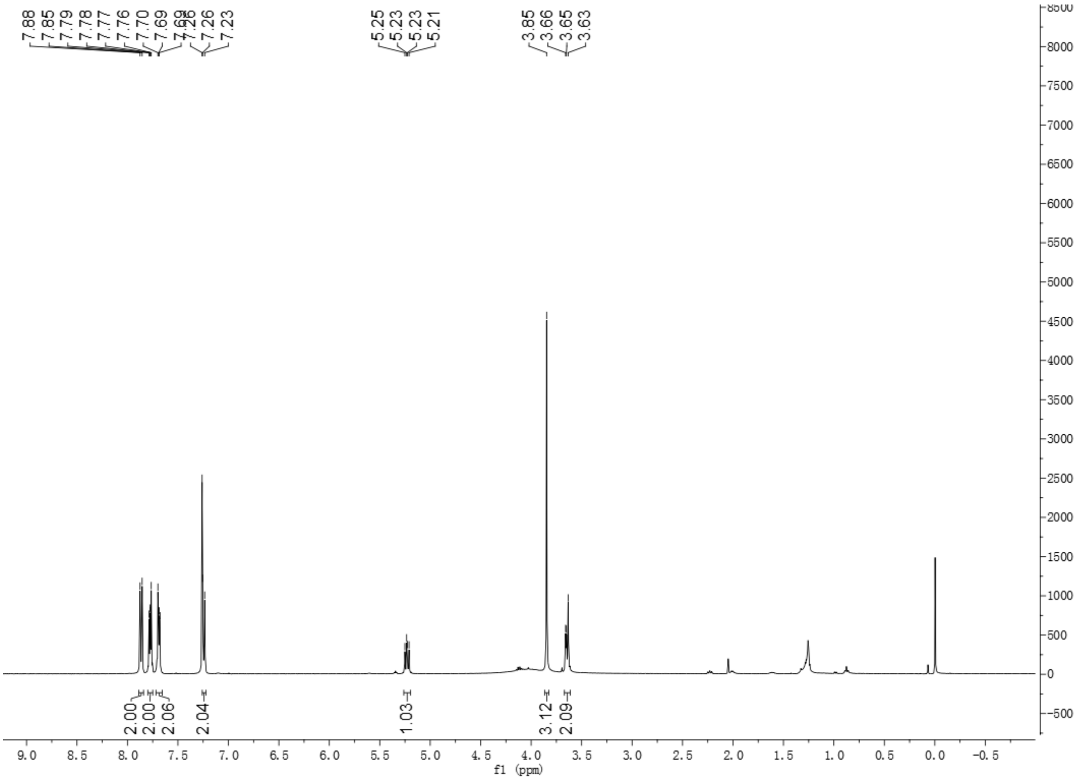

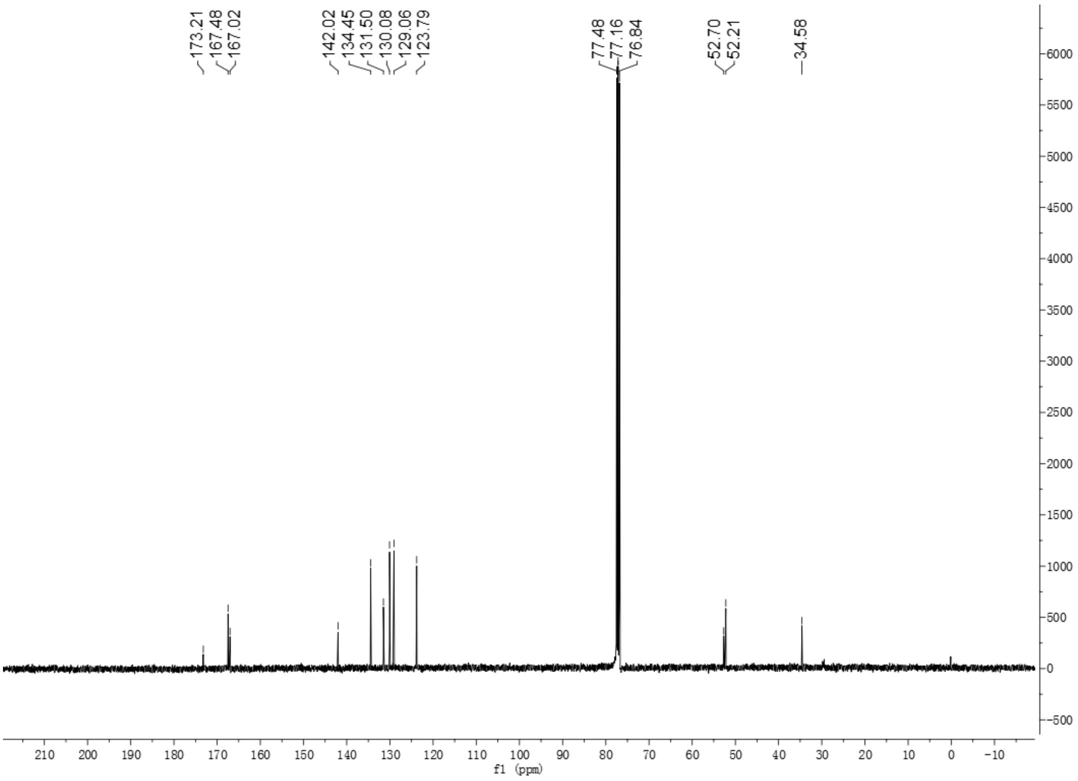

Supplementary Figure 6. ^{1}H and ^{13}C NMR spectra for 3e

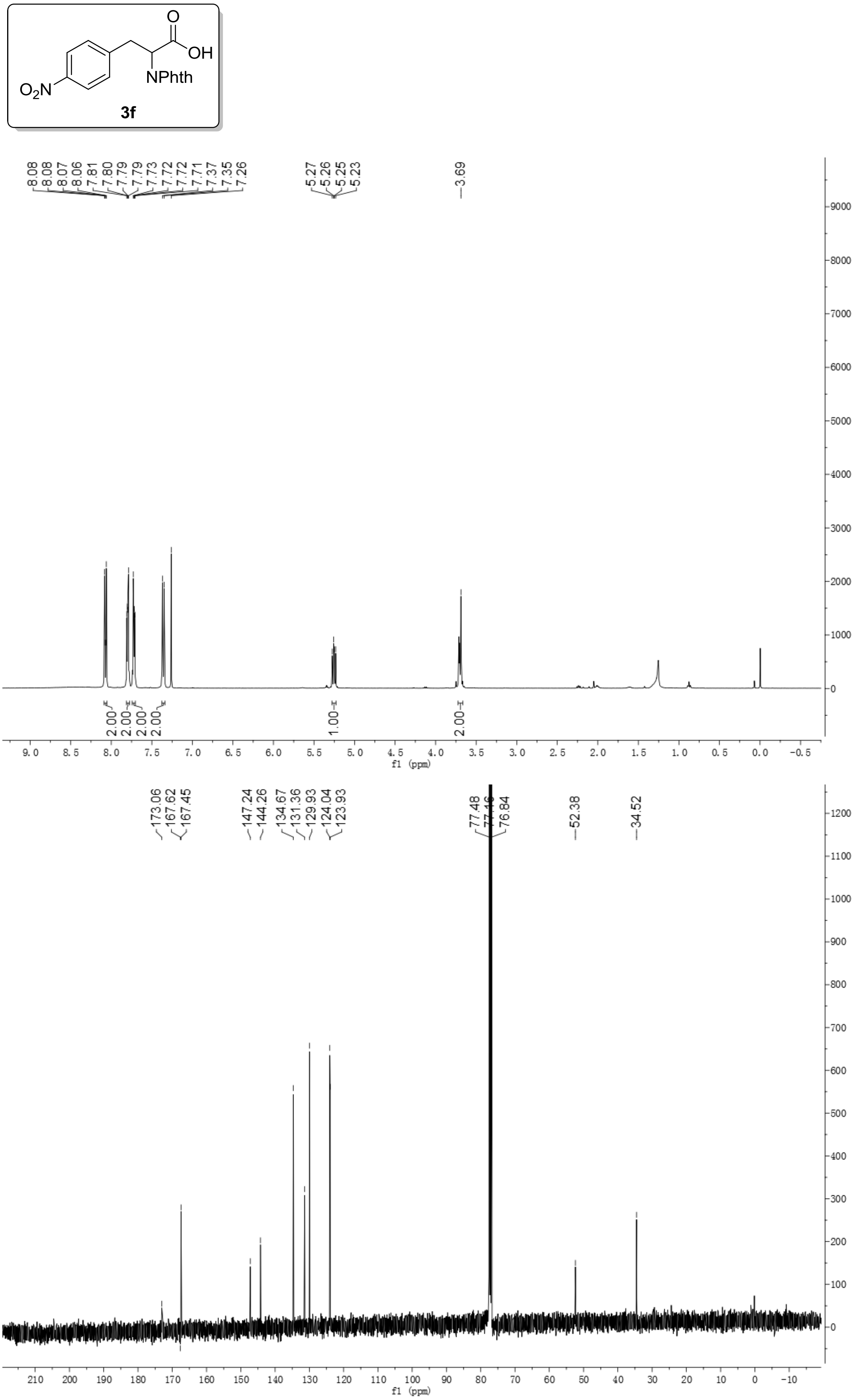

Supplementary Figure 7. ^{1}H and ^{13}C NMR spectra for 3f

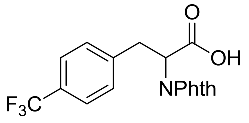
O
OH
NPhth
F3C

3g

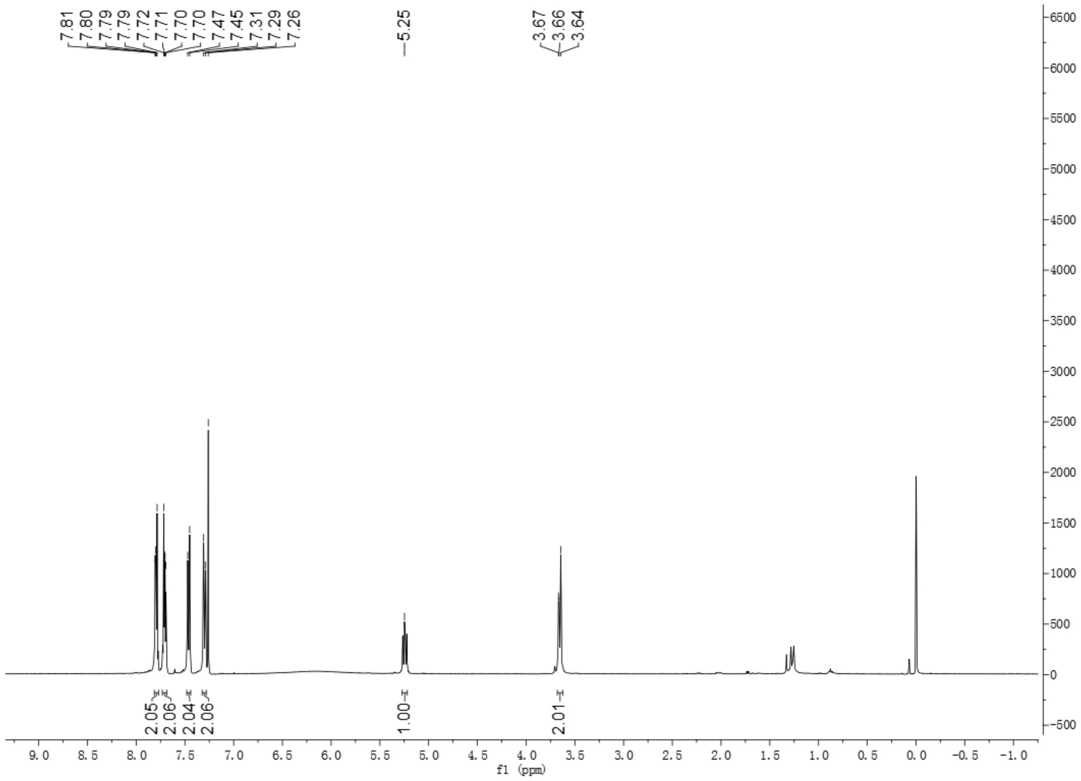
7.81
7.80
7.79
7.79
7.72
7.71
7.70
7.70
7.47
7.45
7.31
7.29
7.26
5.25
3.67
3.66
3.64
2.05
2.06
2.04
2.06
1.00
2.01
f1 (ppm)

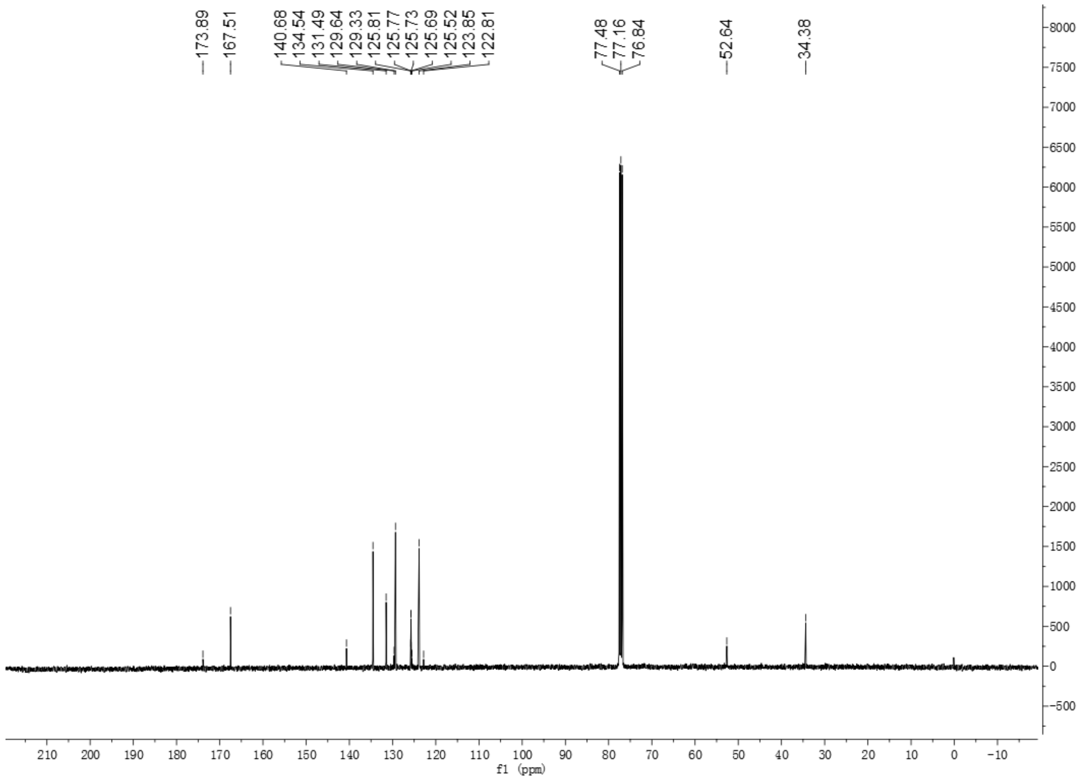
173.89
167.51
140.68
134.54
131.49
129.64
129.33
125.81
125.77
125.73
125.69
125.52
123.85
122.81
77.48
77.16
76.84
52.64
34.38
f1 (ppm)

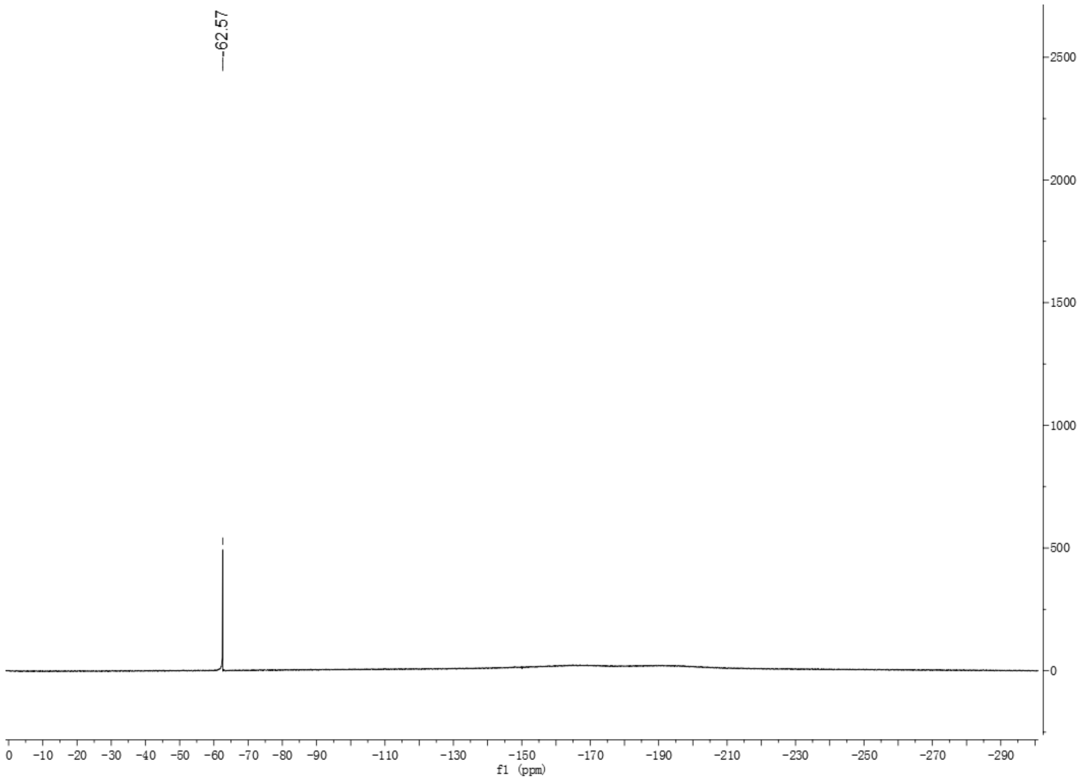

Supplementary Figure 8. ^{1}H ,^{19}F and ^{13}C NMR spectra for 3g

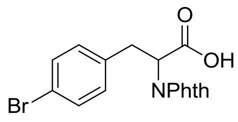

3h

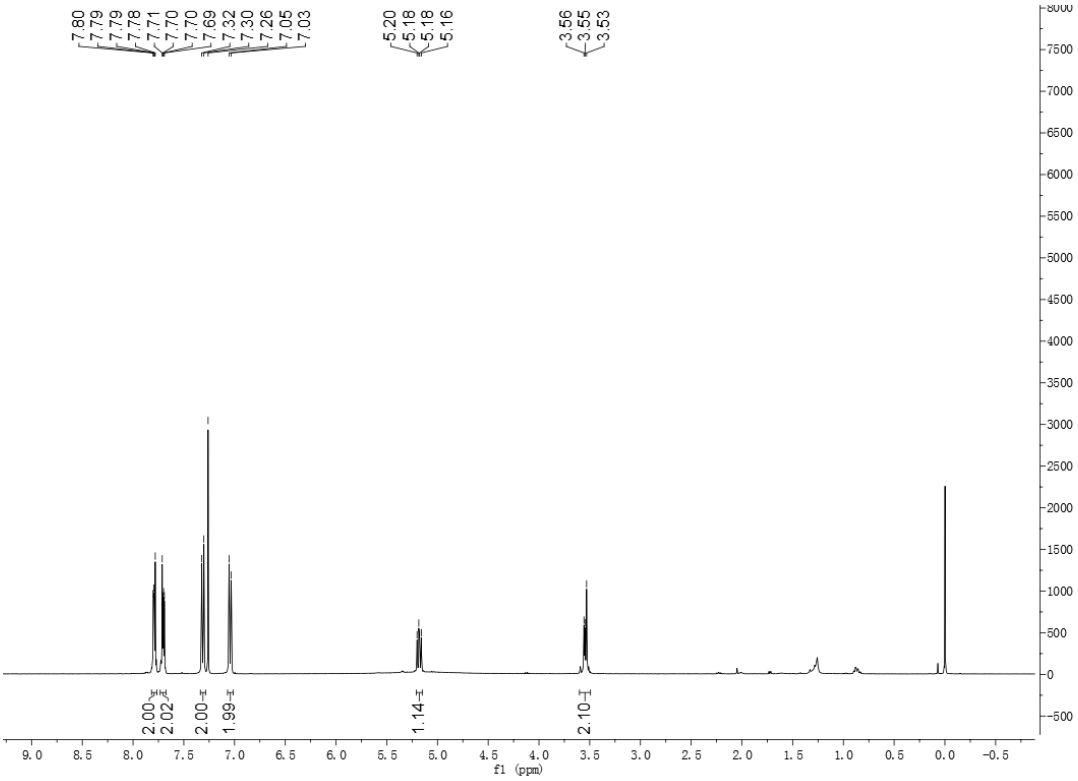

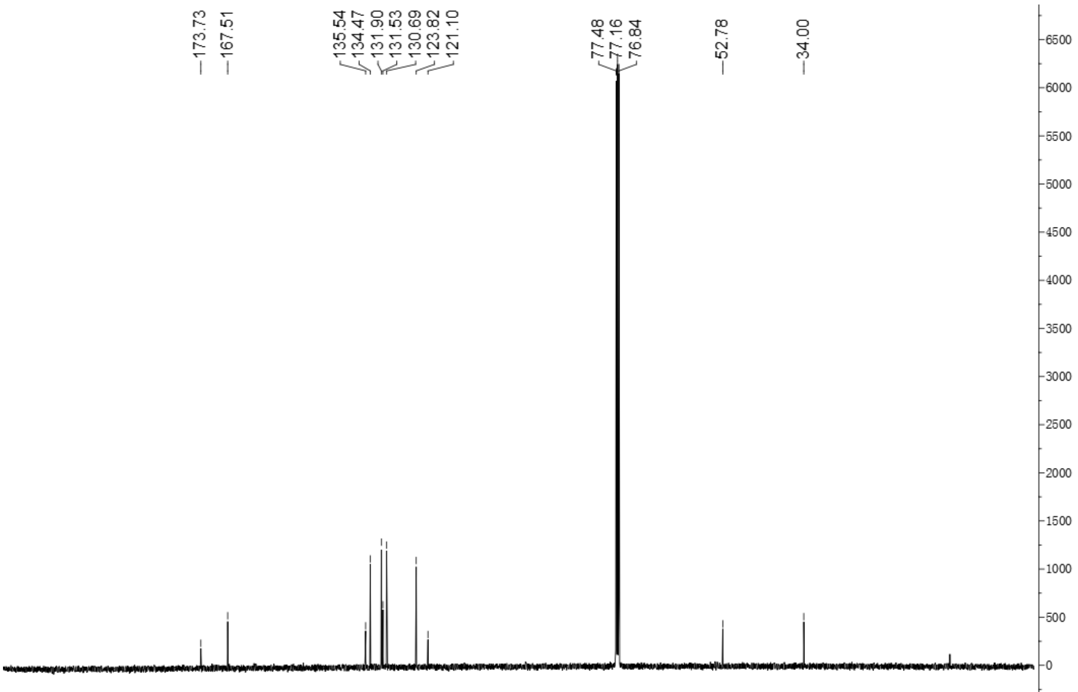

Supplementary Figure 9. ^{1}H and ^{13}C NMR spectra for 3h

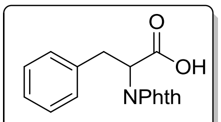

3i

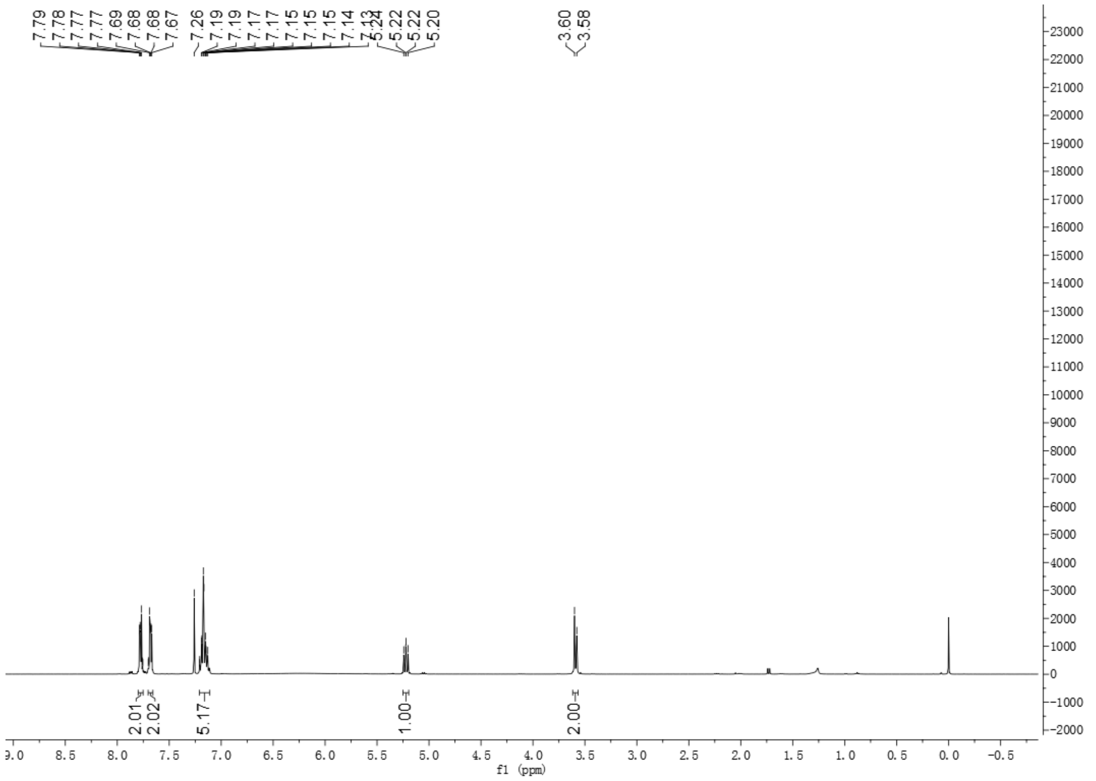

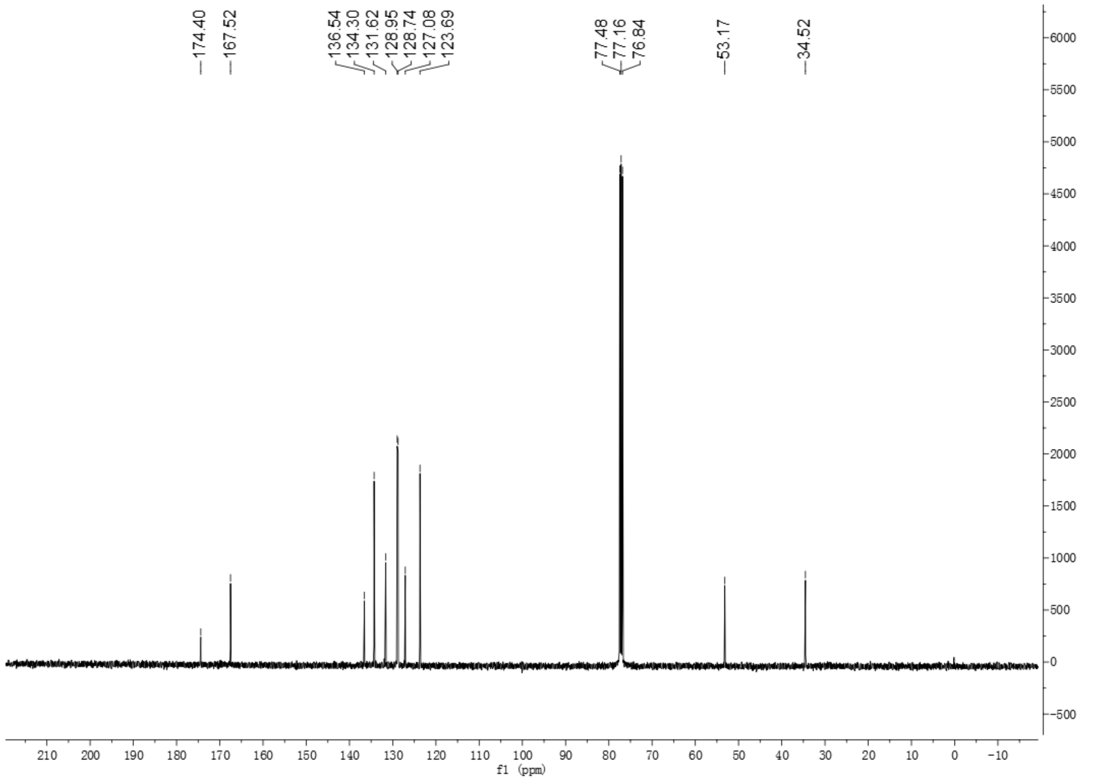

Supplementary Figure 10. ^{1}H and ^{13}C NMR spectra for 3i

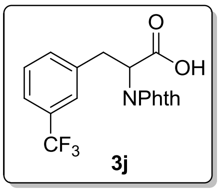

3j

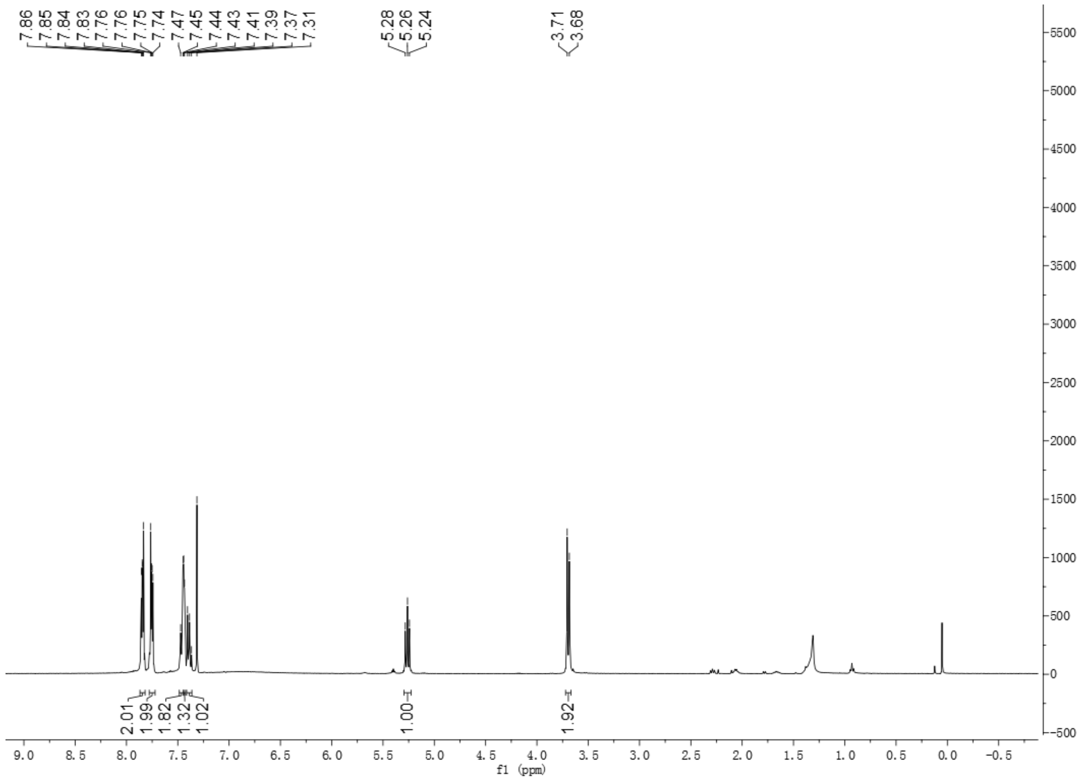

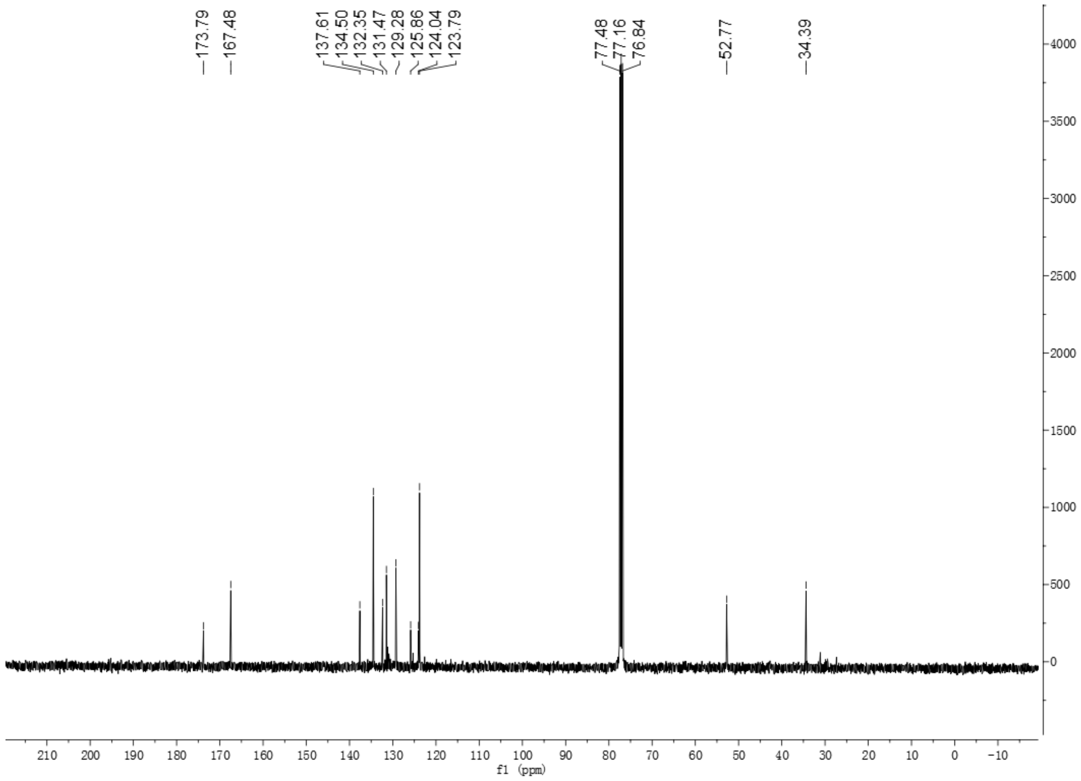

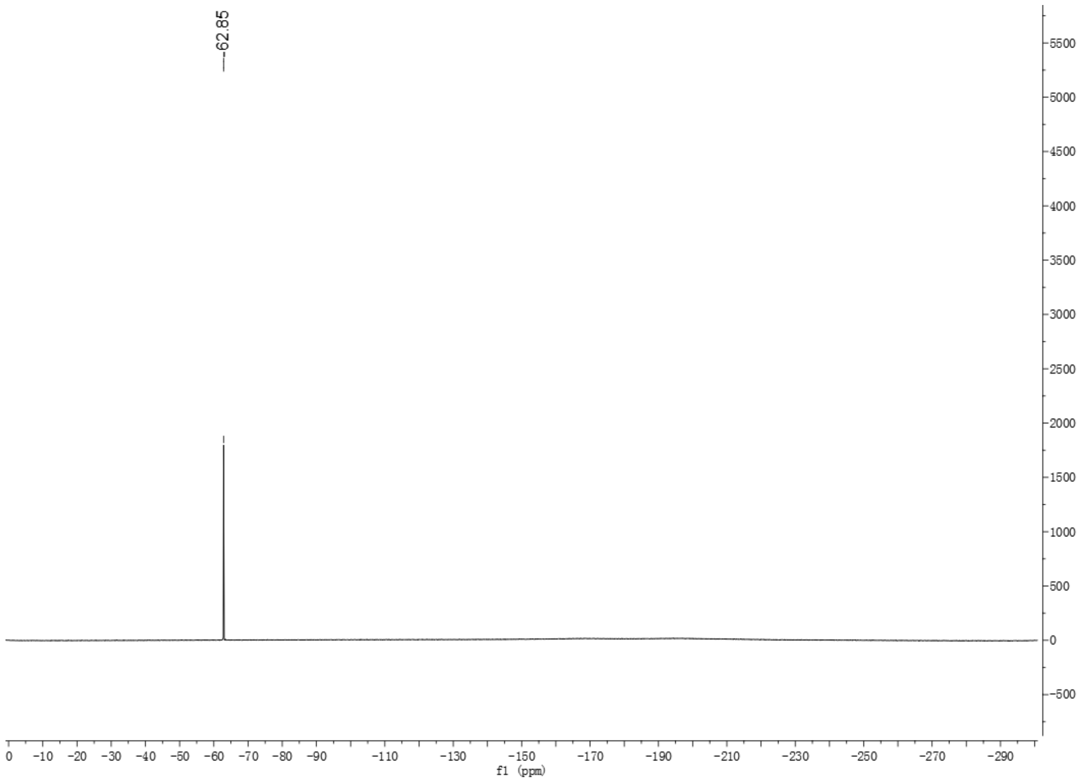

Supplementary Figure 11. ^{1}H, ^{19}F and ^{13}C NMR spectra for 3j

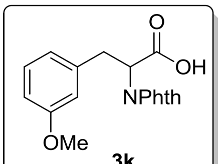

3k

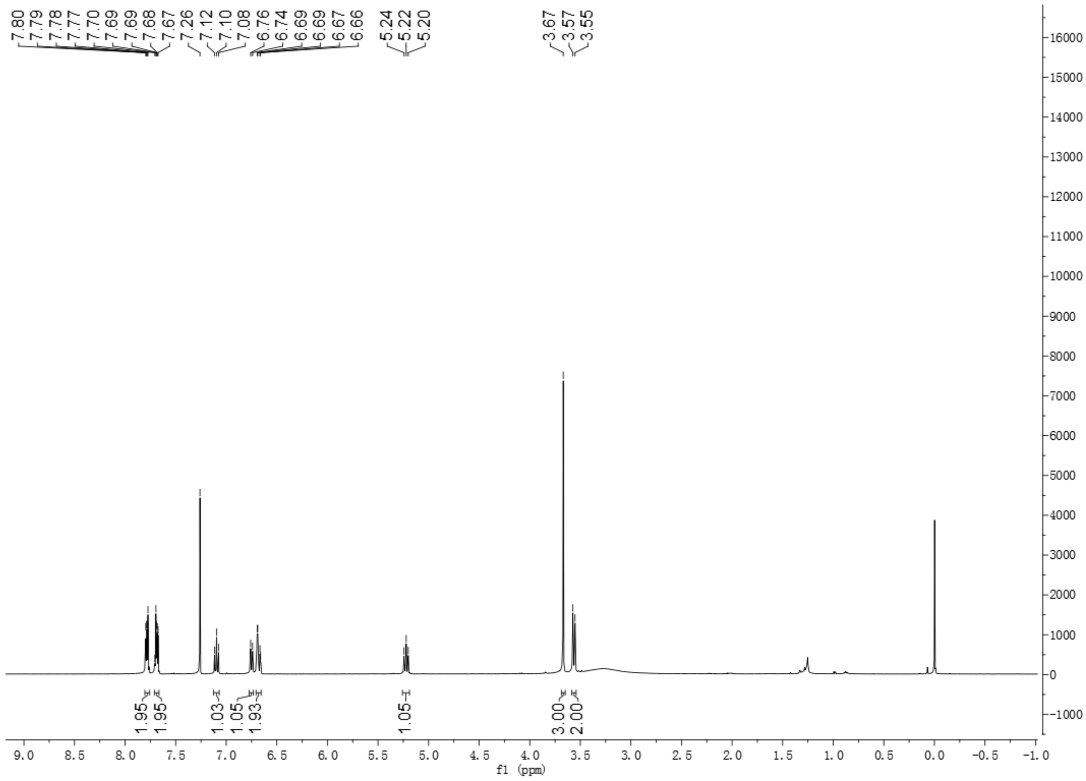

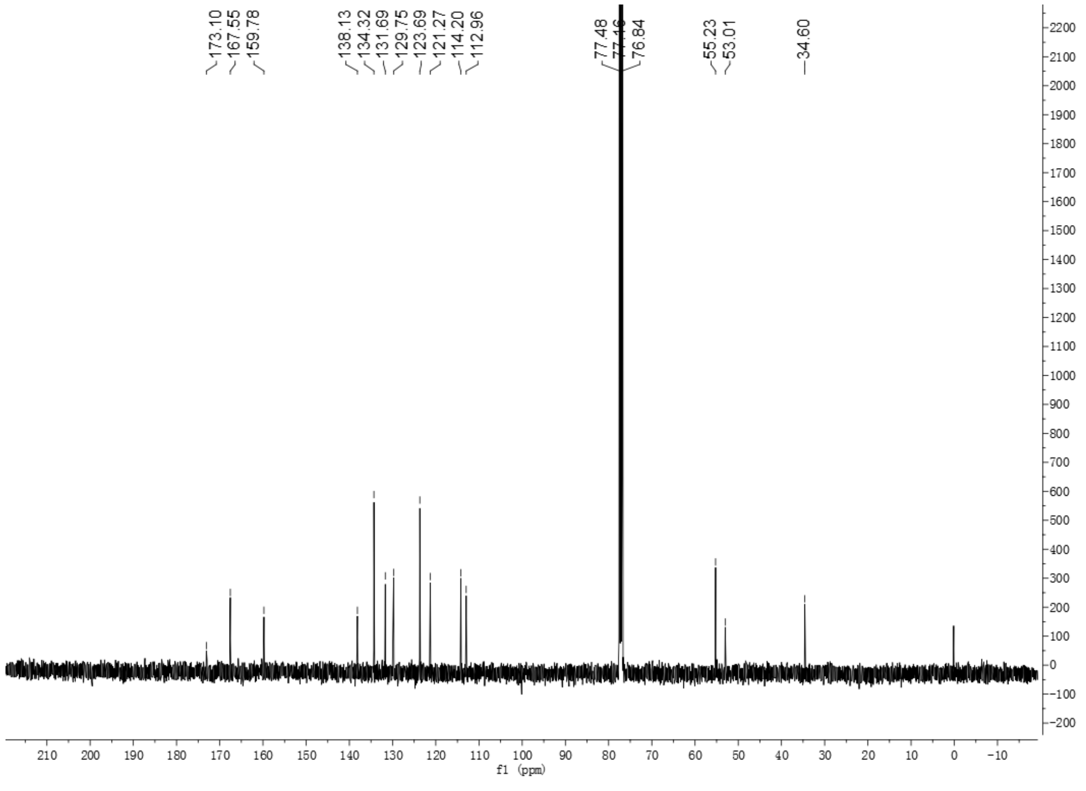

Supplementary Figure 12. ^{1}H and ^{13}C NMR spectra for 3k

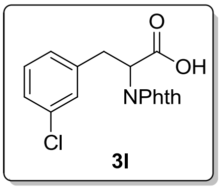

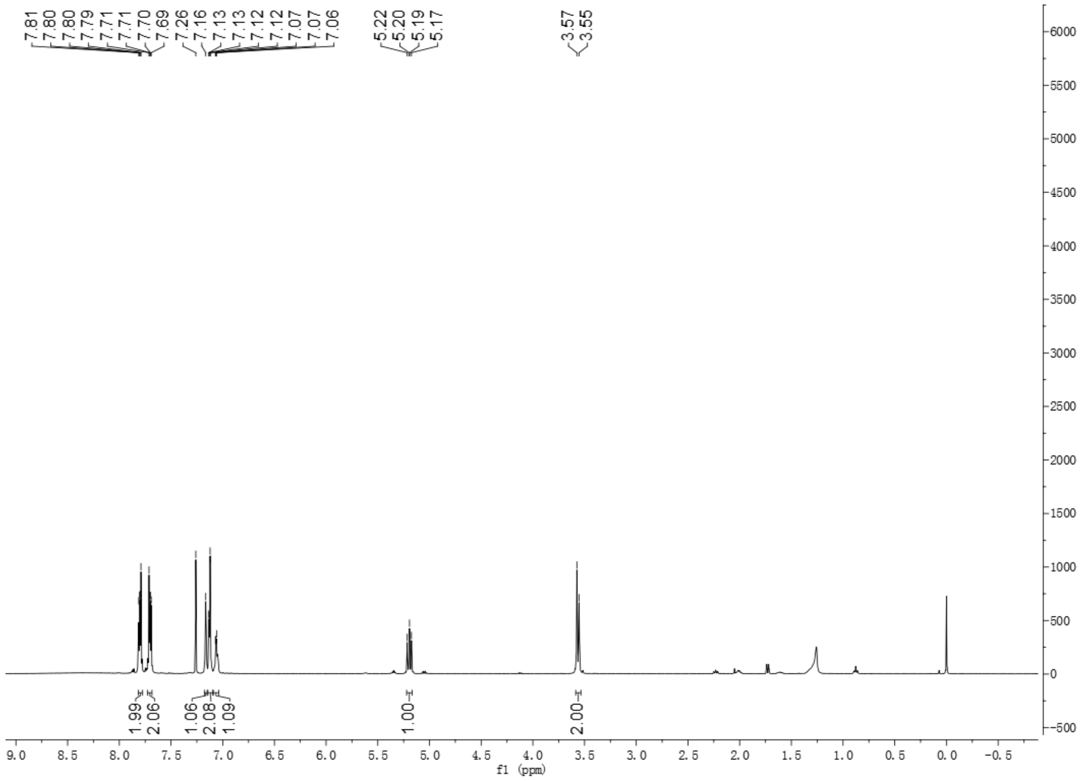

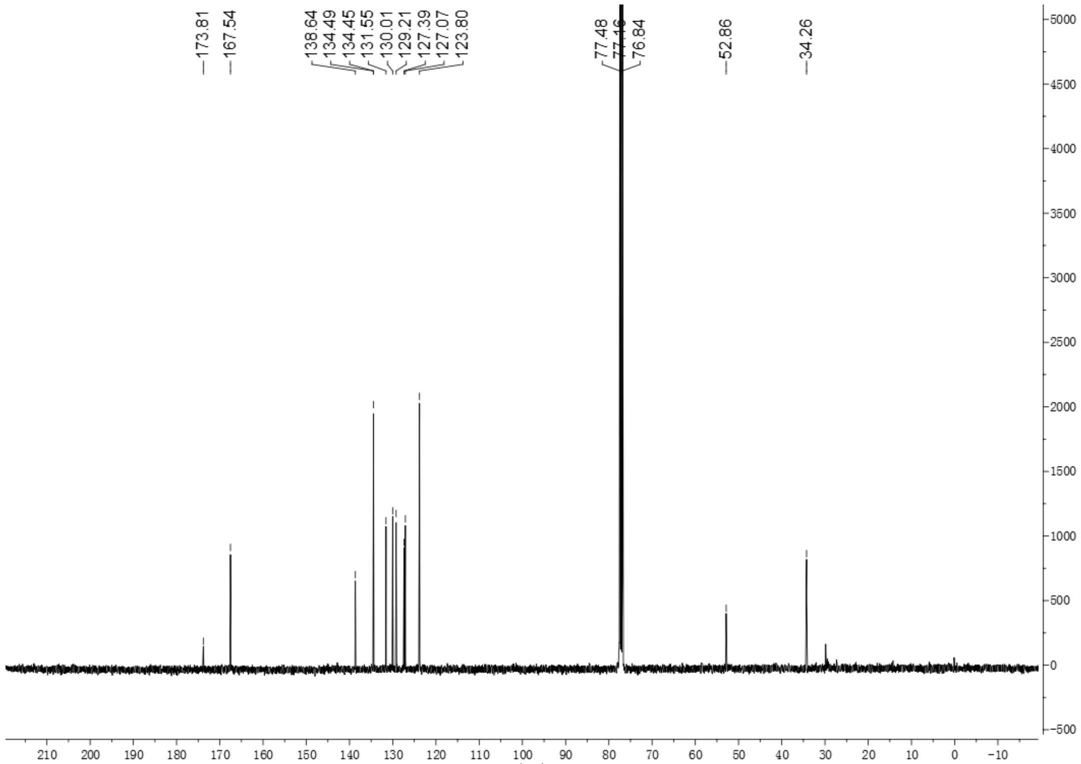

Supplementary Figure 13. ^{1}H and ^{13}C NMR spectra for 3l

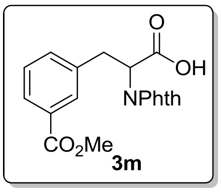

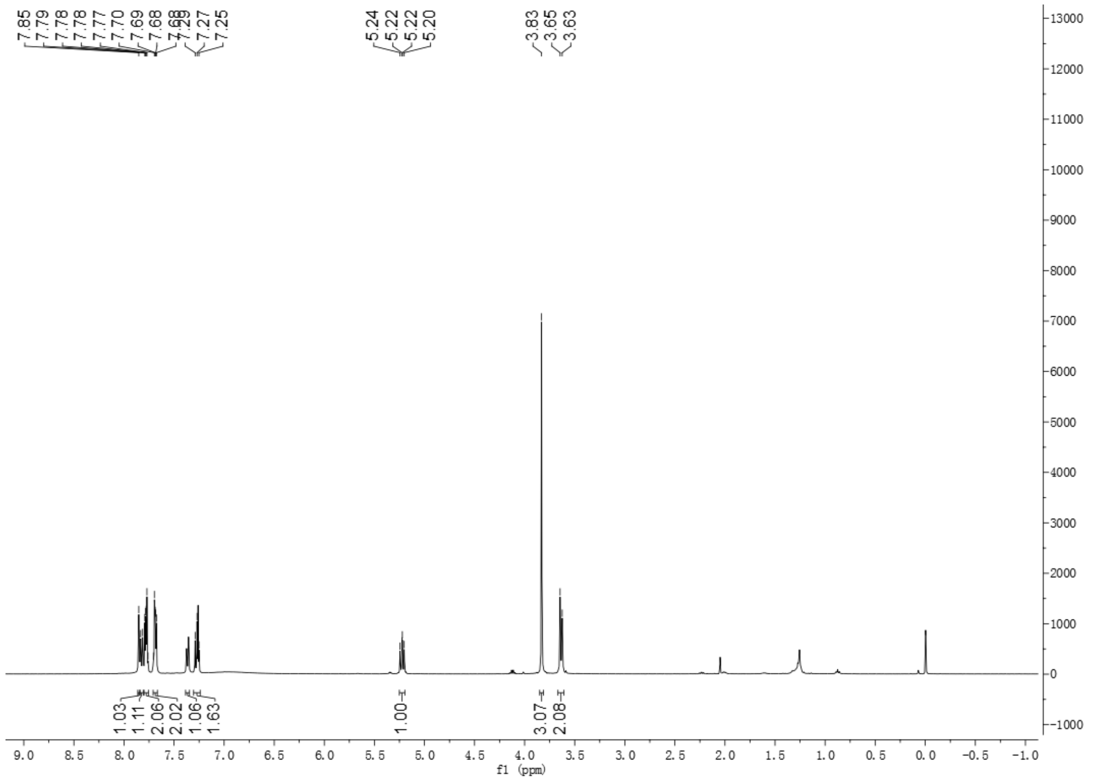

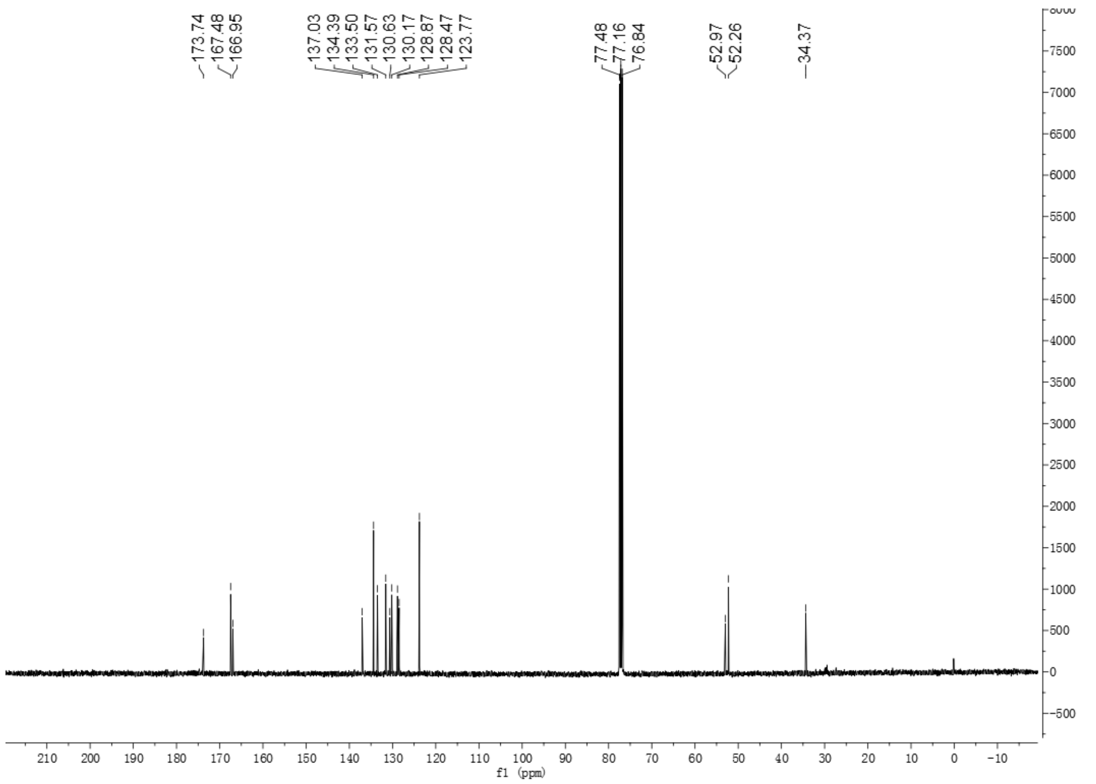

Supplementary Figure 14. ^{1}H and ^{13}C NMR spectra for 3m

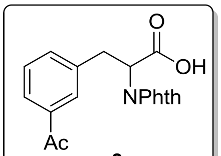

3n

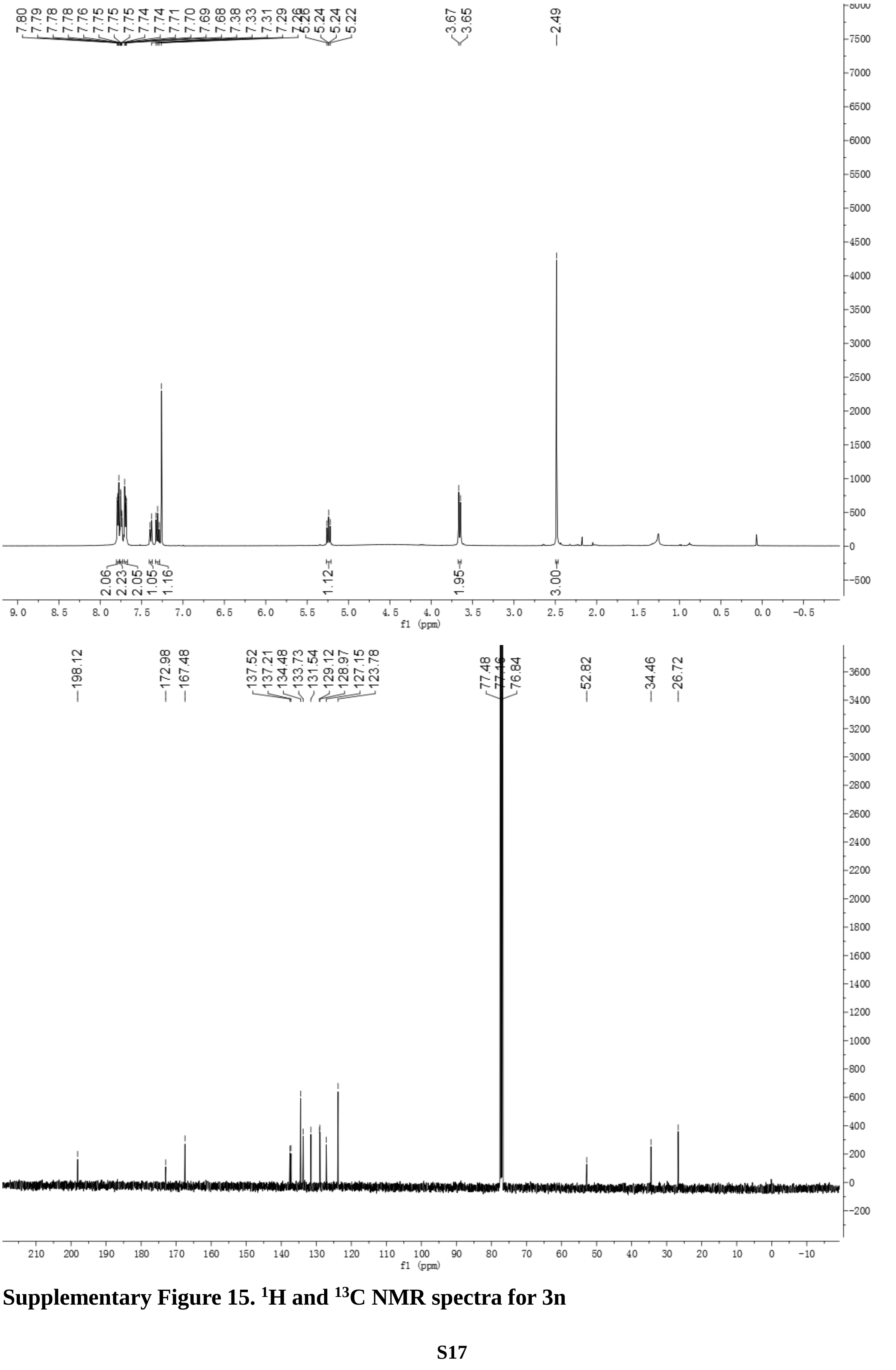

Supplementary Figure 15. ^{1}H and ^{13}C NMR spectra for 3n

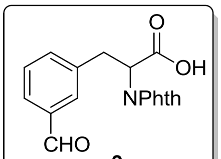

3o

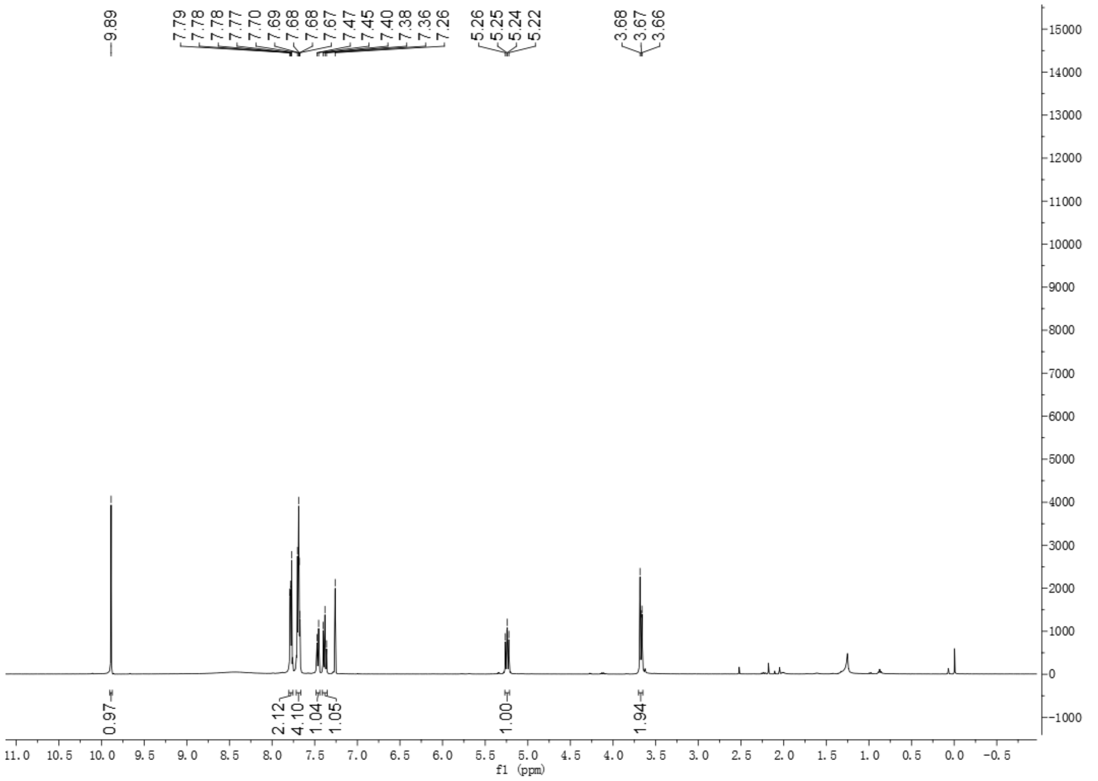

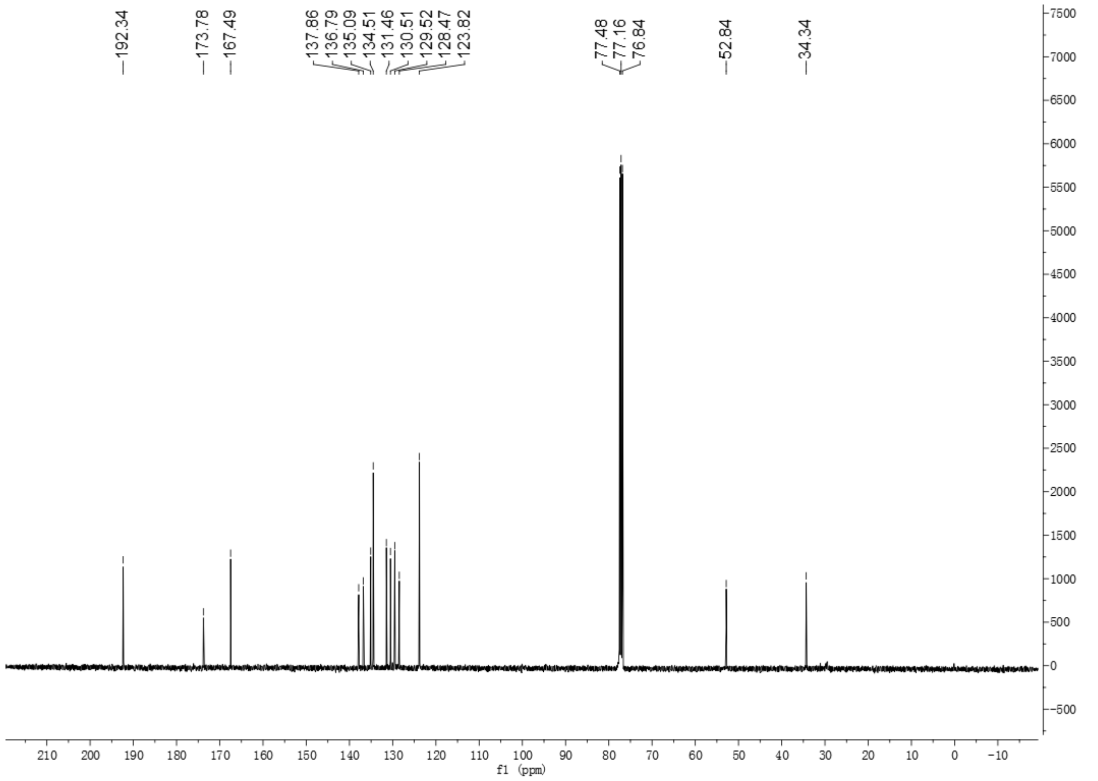

Supplementary Figure 16. ^{1}H and ^{13}C NMR spectra for 3o

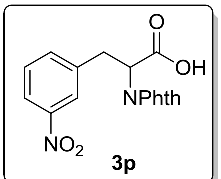

3p

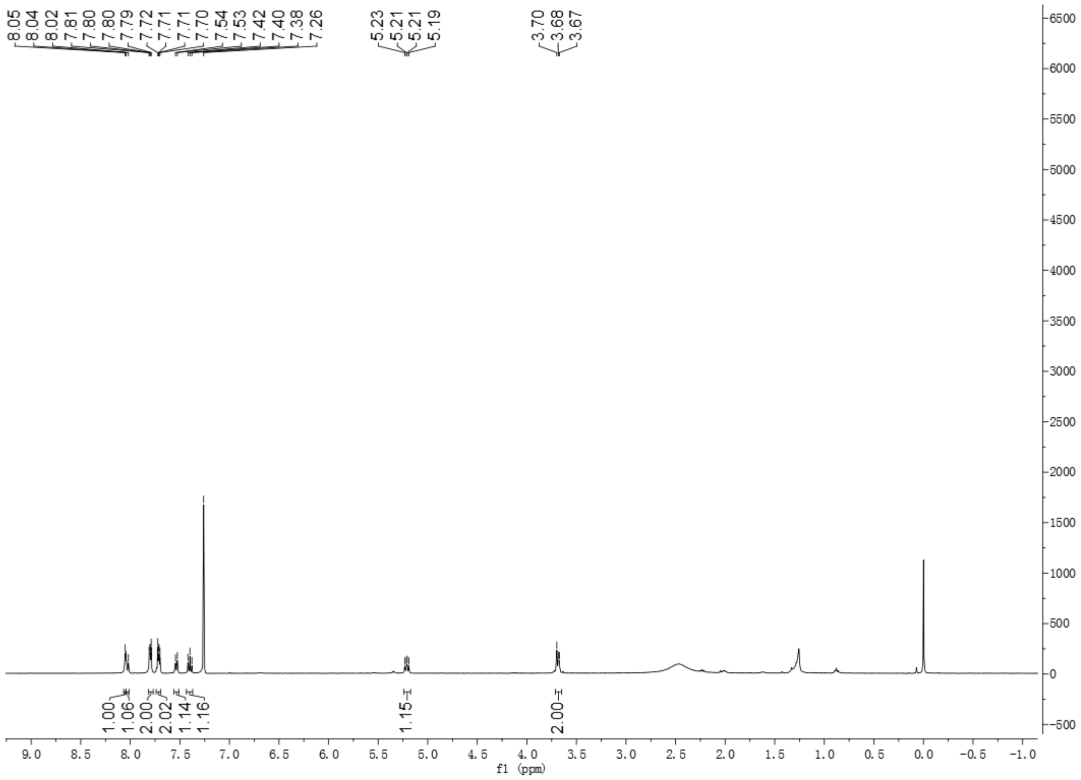

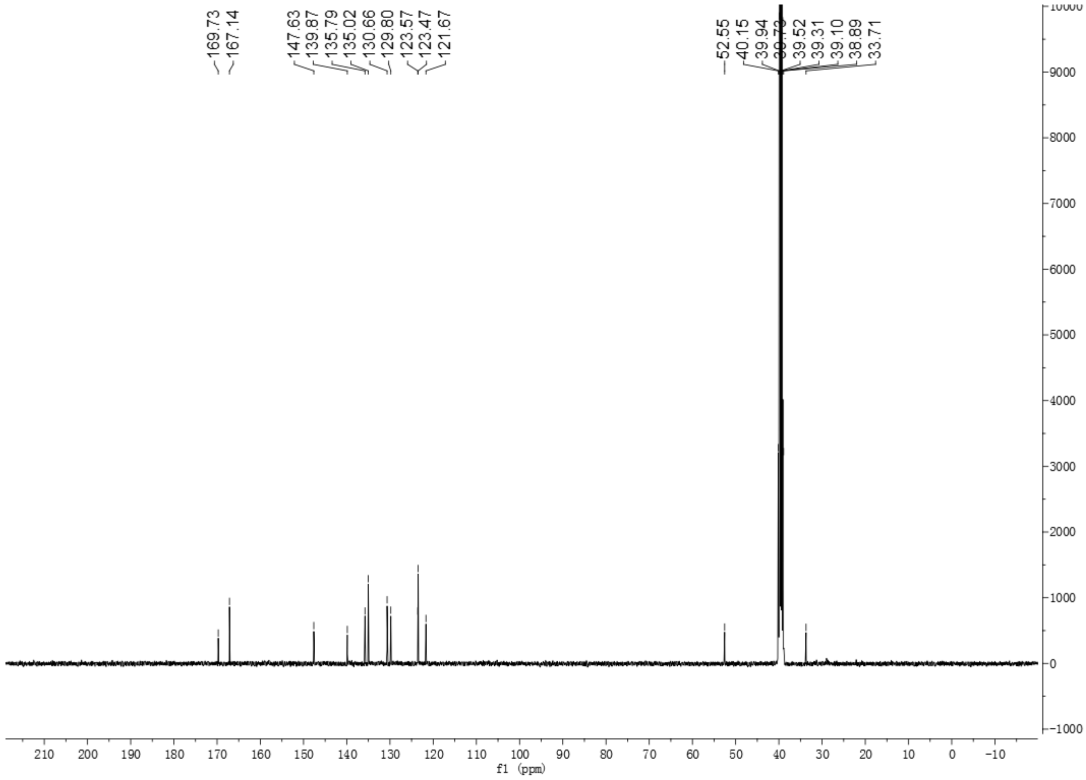

Supplementary Figure 17. ^{1}H and ^{13}C NMR spectra for 3p

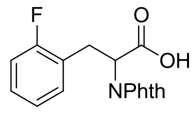
F
O
OH
NPhth

3q

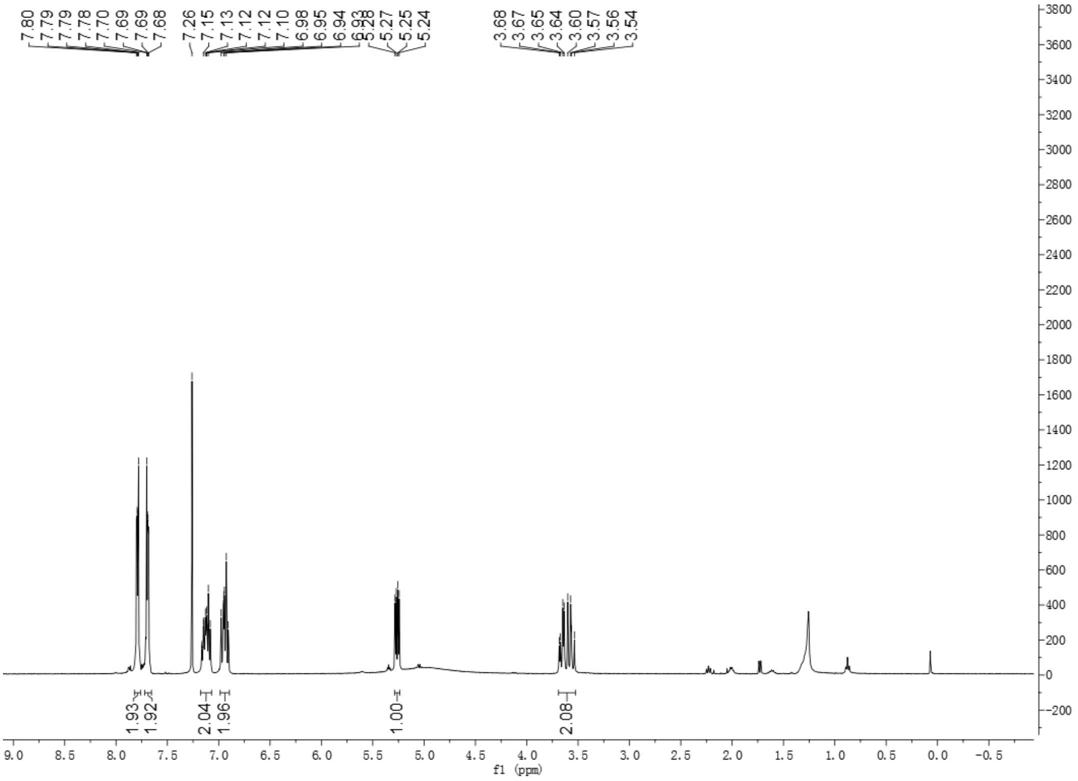
7.80
7.79
7.79
7.78
7.70
7.69
7.69
7.68
7.26
7.15
7.13
7.12
7.12
7.10
6.98
6.95
6.94
6.93
5.28
5.27
5.25
5.24
3.68
3.67
3.65
3.64
3.60
3.57
3.56
3.54
1.93
1.92
2.04
1.96
1.00
2.08
f1 (ppm)

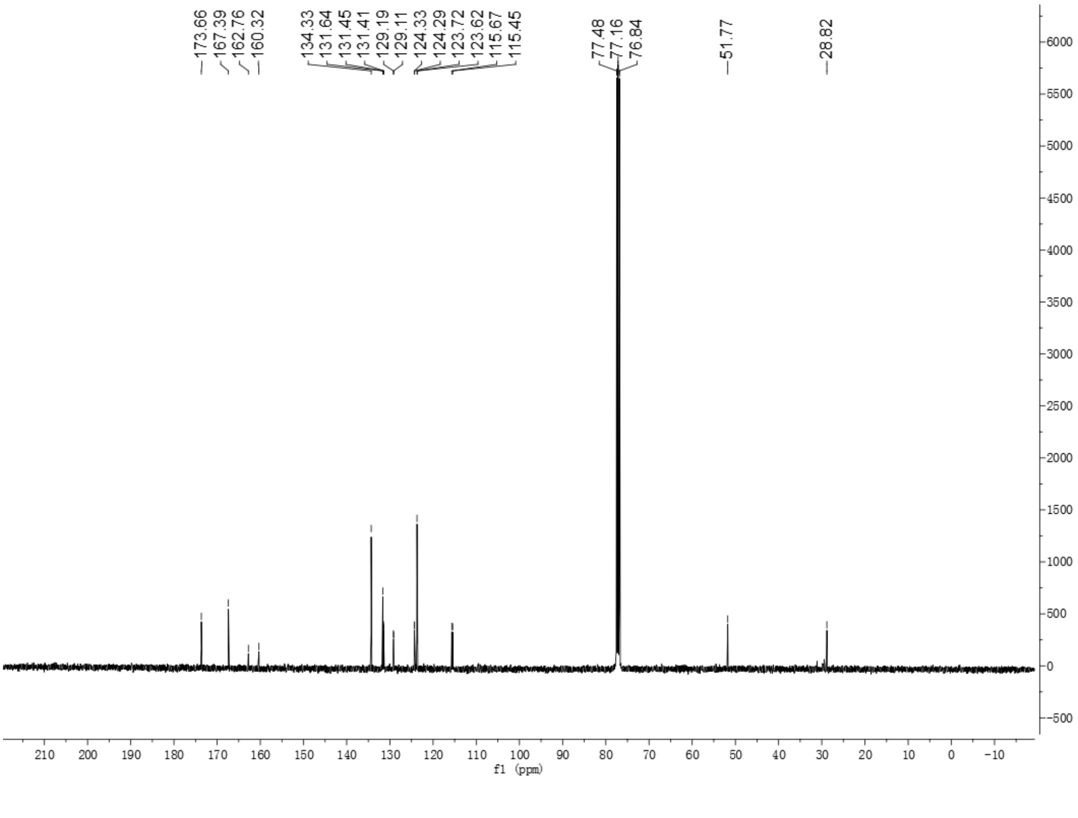
173.66
167.39
162.76
160.32
134.33
131.64
131.45
131.41
129.19
129.11
124.33
124.29
123.72
123.62
115.67
115.45
77.48
77.16
76.84
51.77
28.82
f1 (ppm)

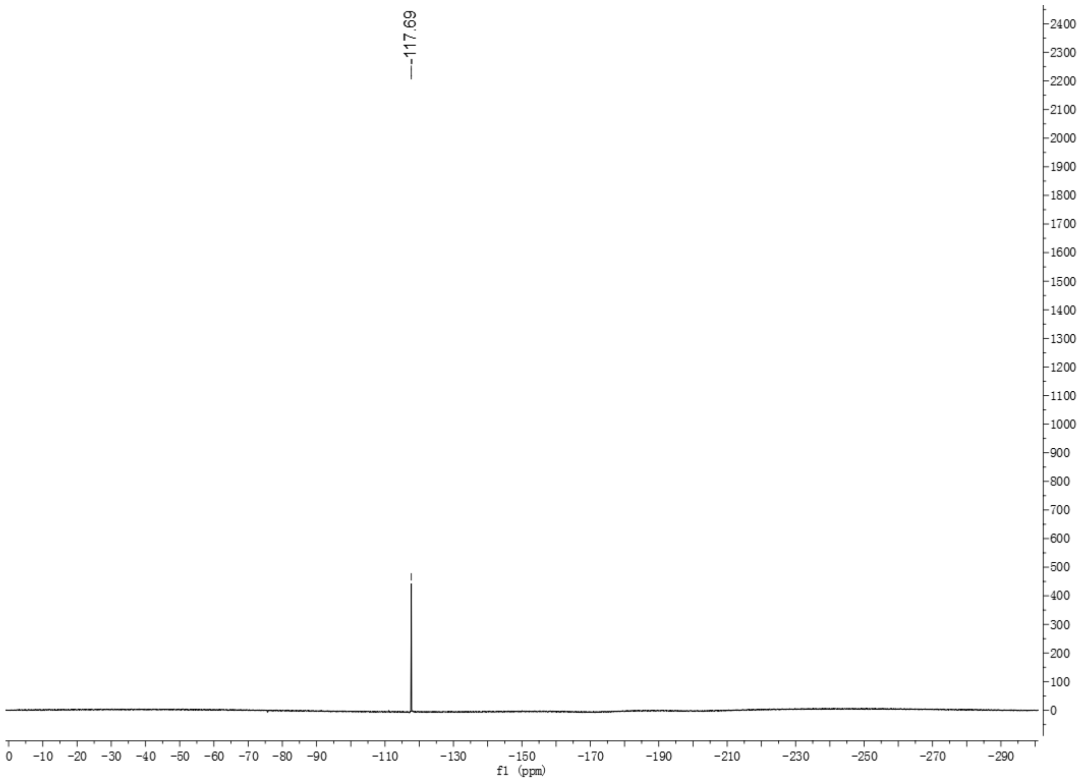

Supplementary Figure 18. ^{1}H, ^{19}F and ^{13}C NMR spectra for 3q

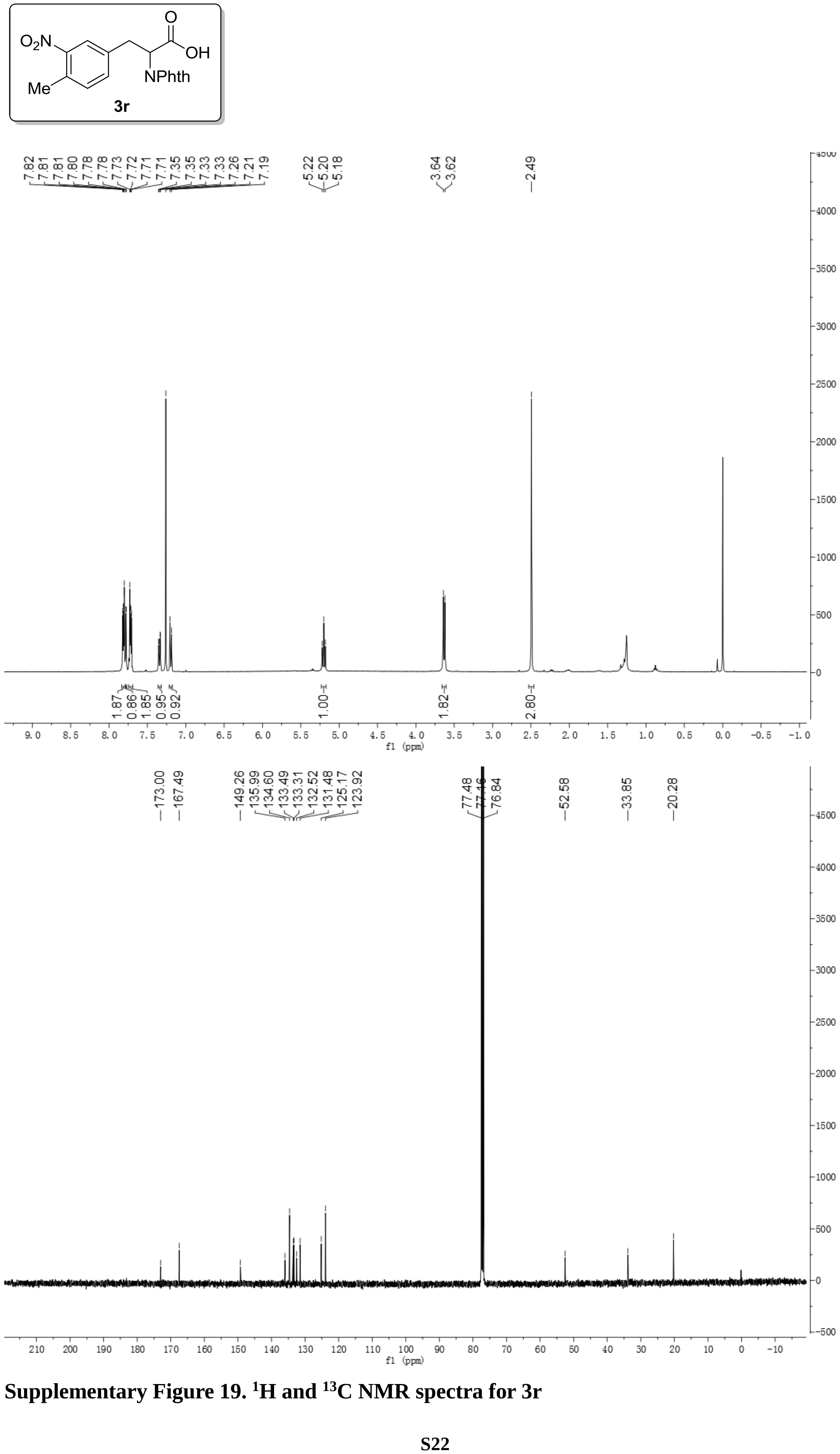

Supplementary Figure 19. ^{1}H **and** ^{13}C **NMR spectra for 3r**

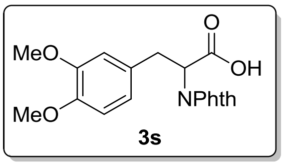

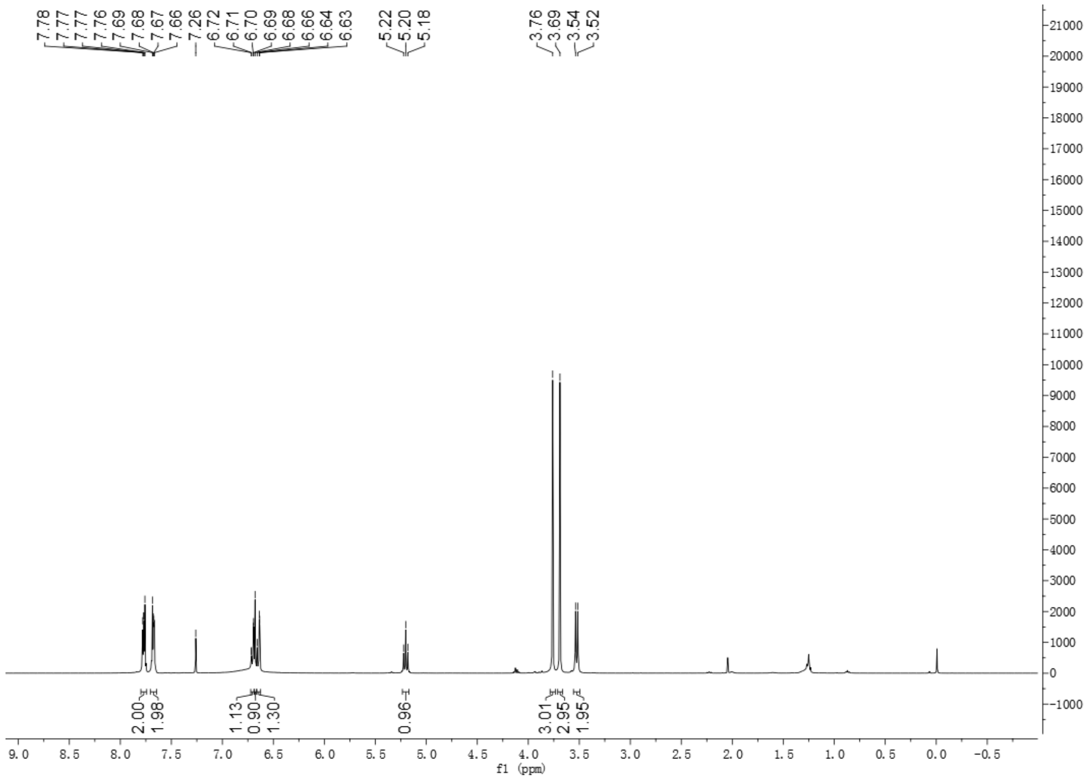

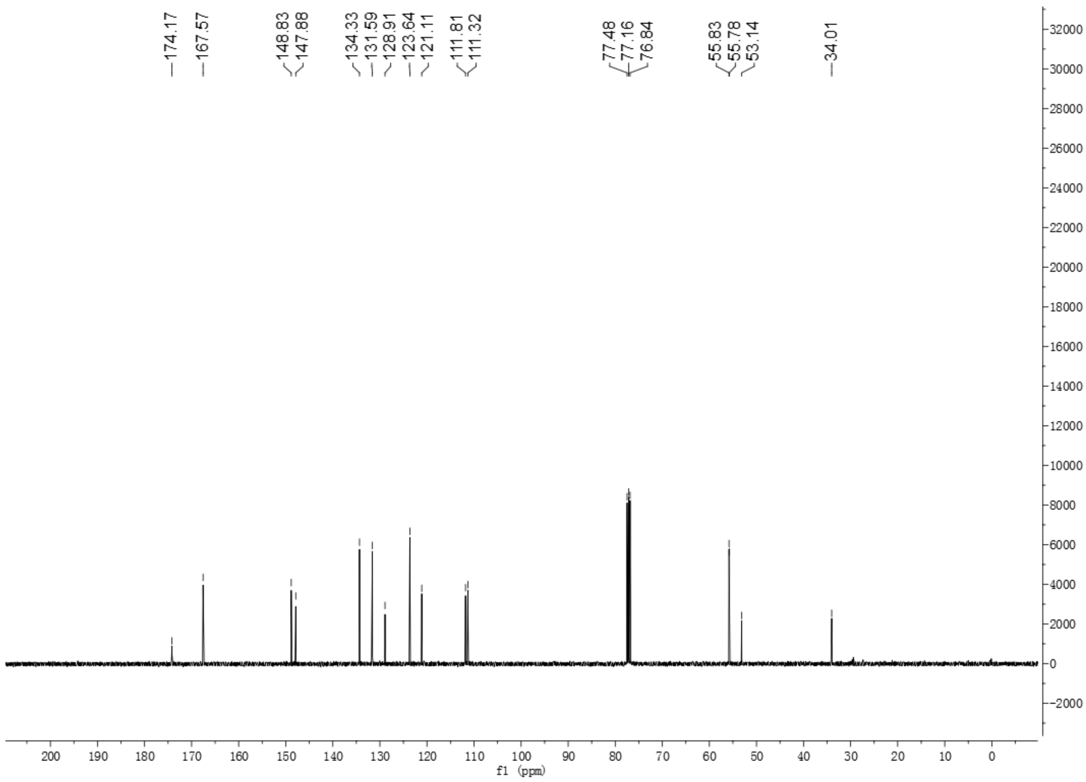

Supplementary Figure 20. ¹H and ¹³C NMR spectra for 3s

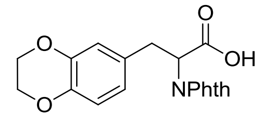

3t

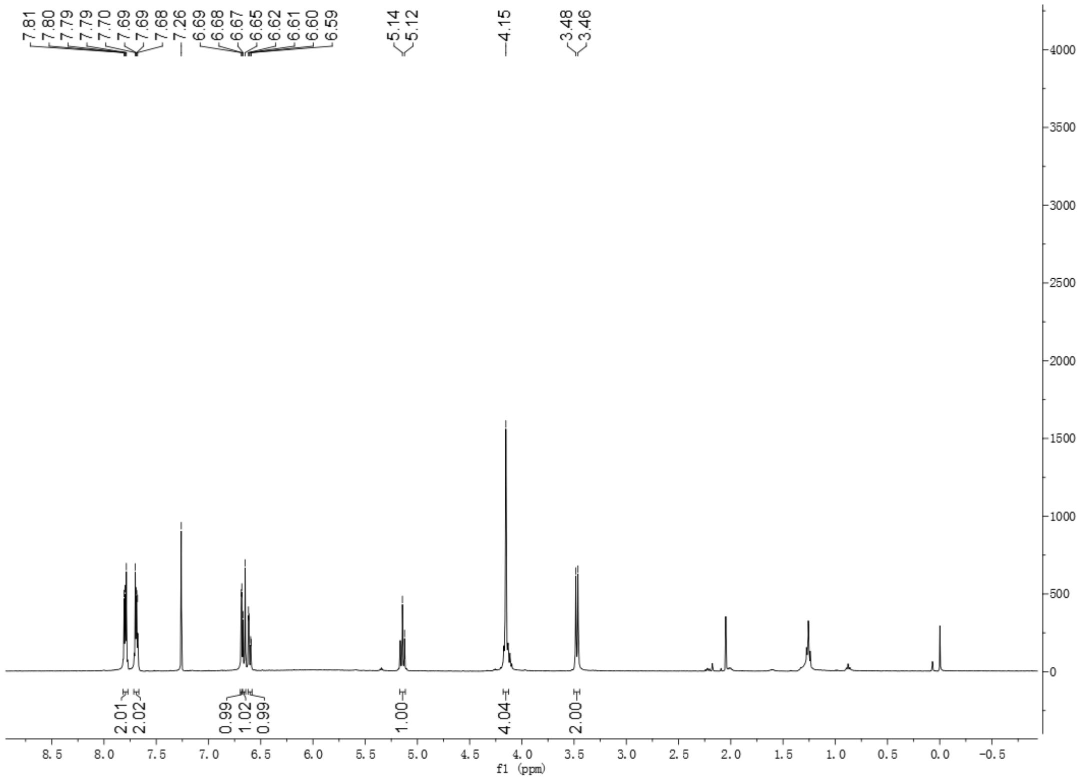

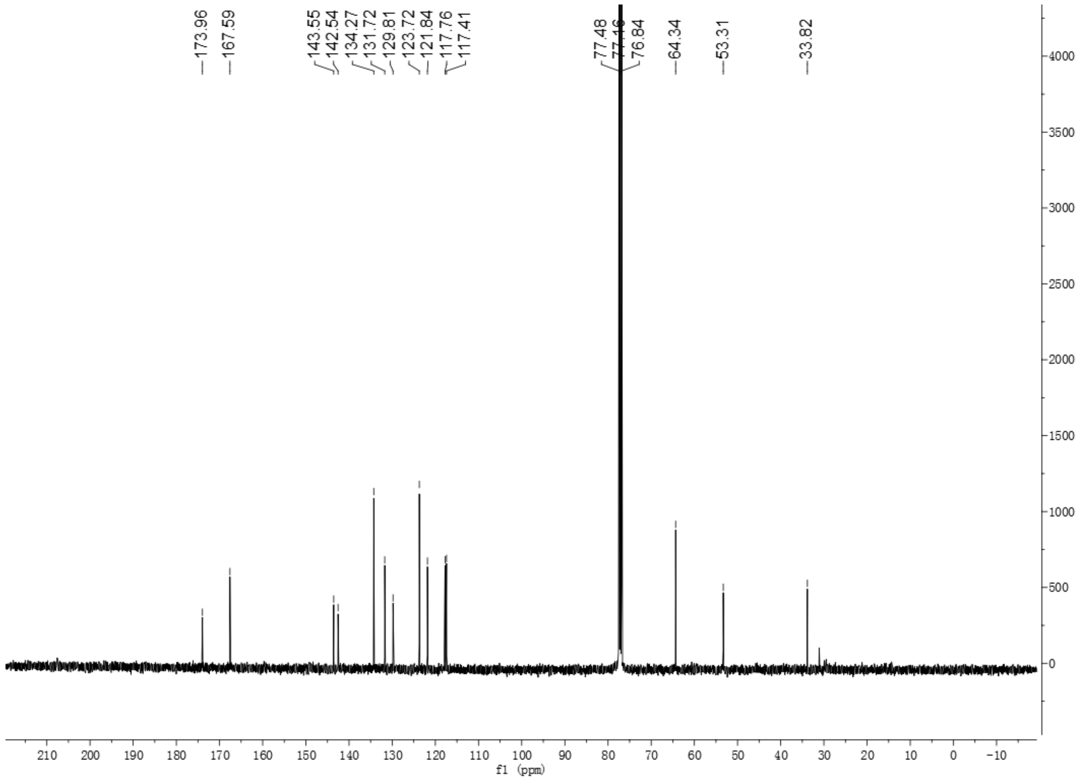

Supplementary Figure 21. ^{1}H and ^{13}C NMR spectra for 3t

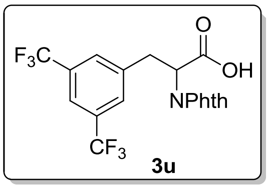

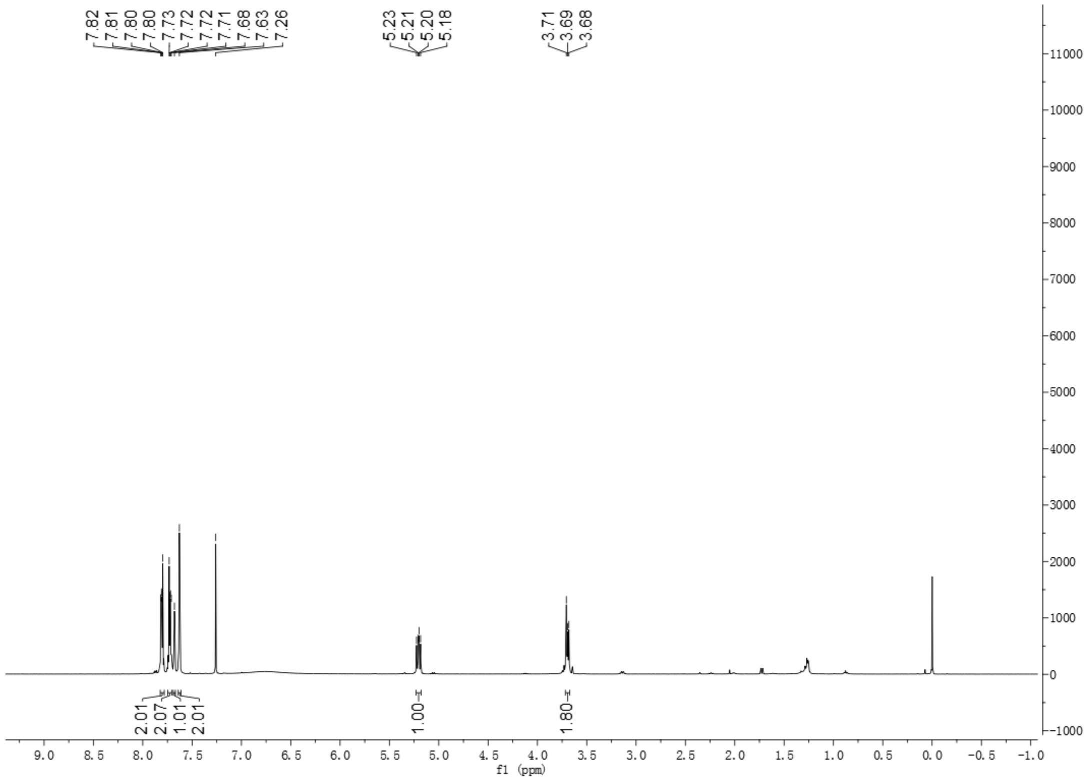

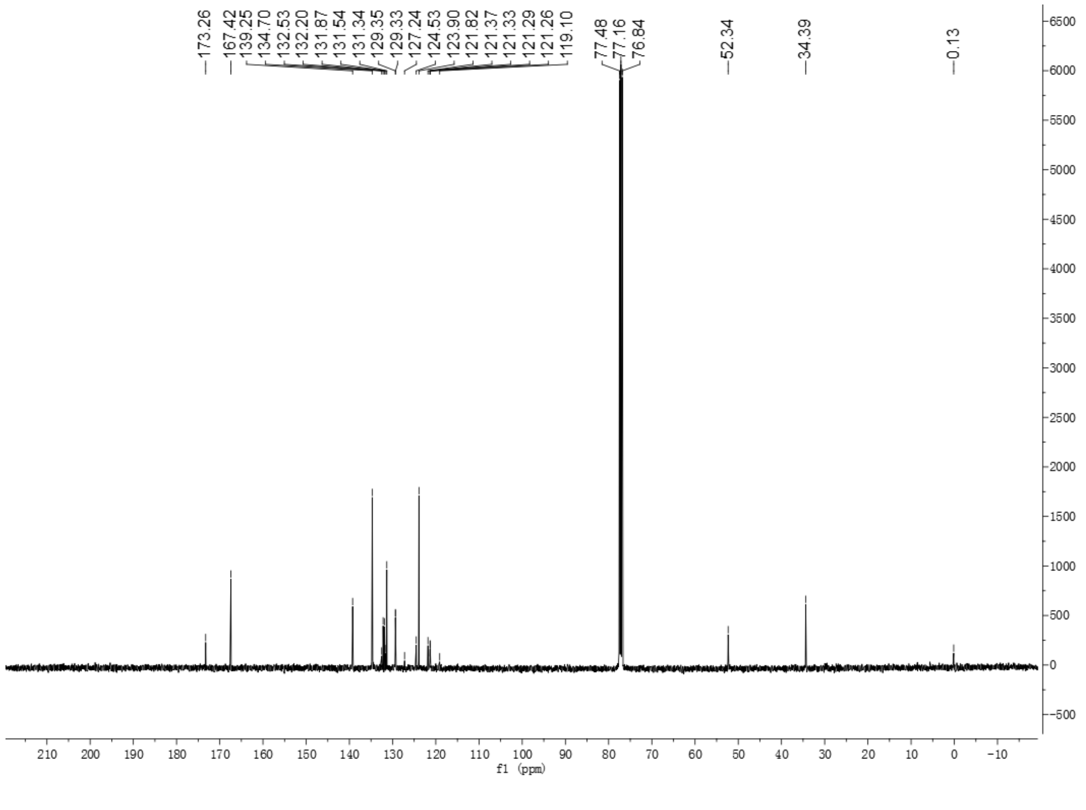

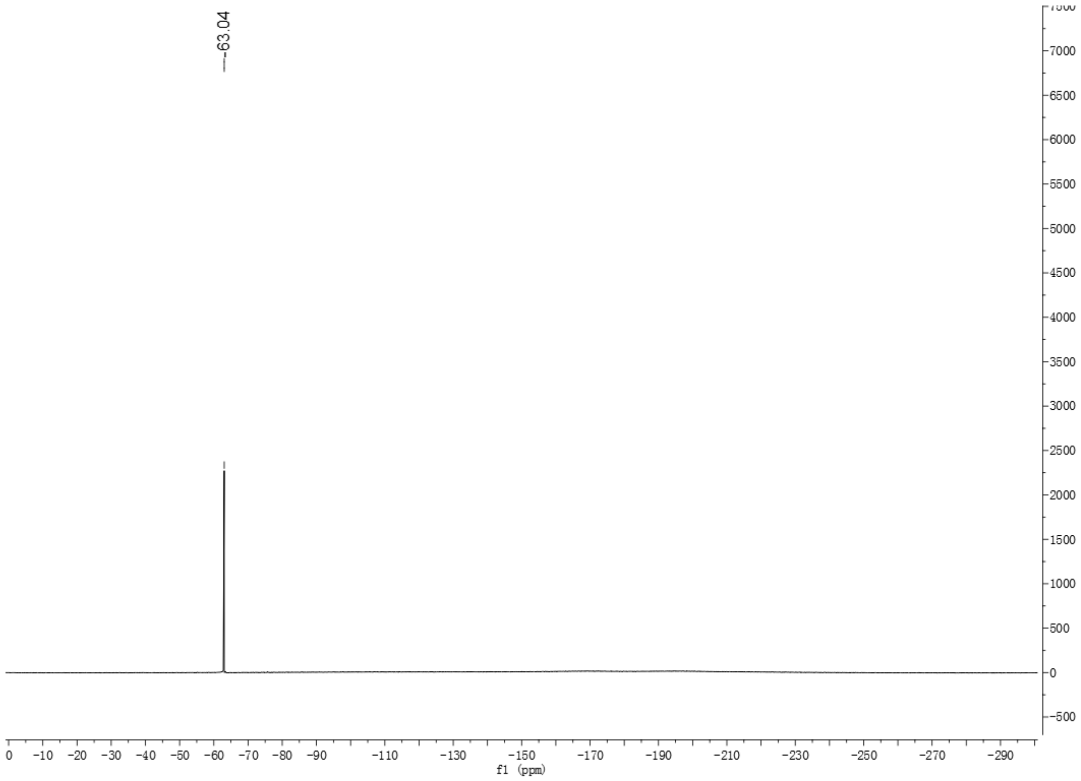

Supplementary Figure 22. ^{1}H, ^{19}F and ^{13}C NMR spectra for 3u

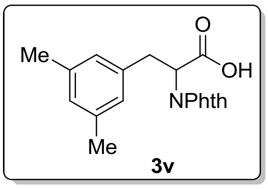

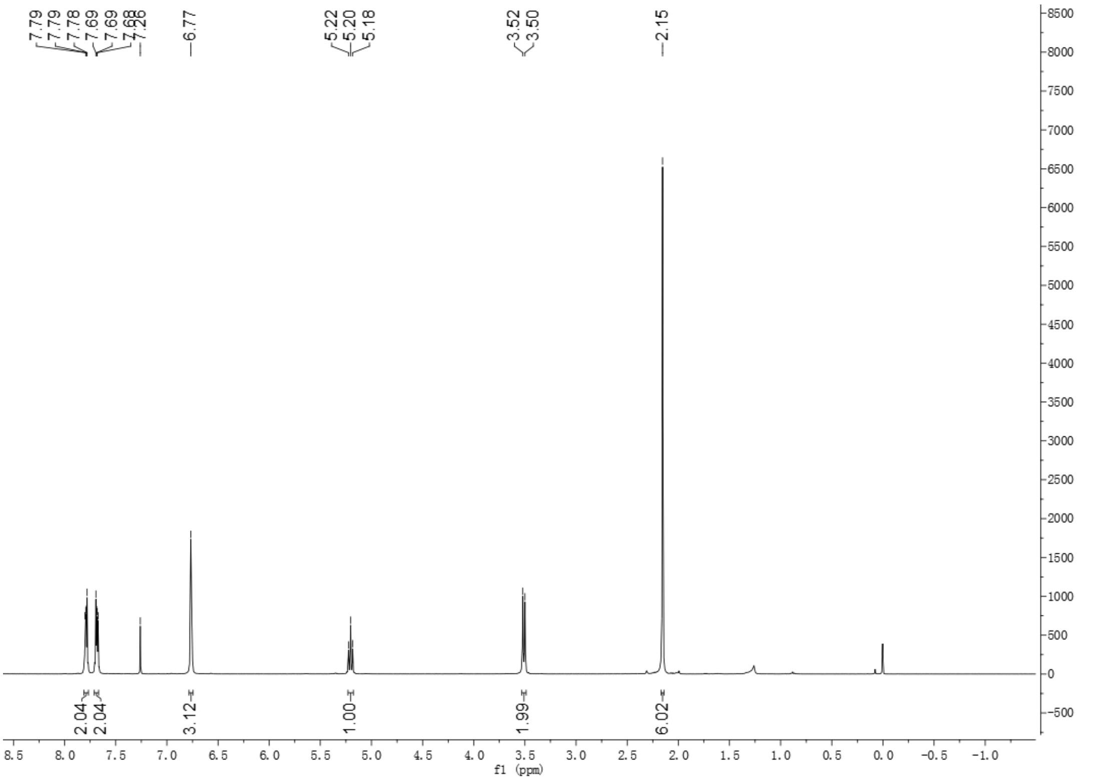

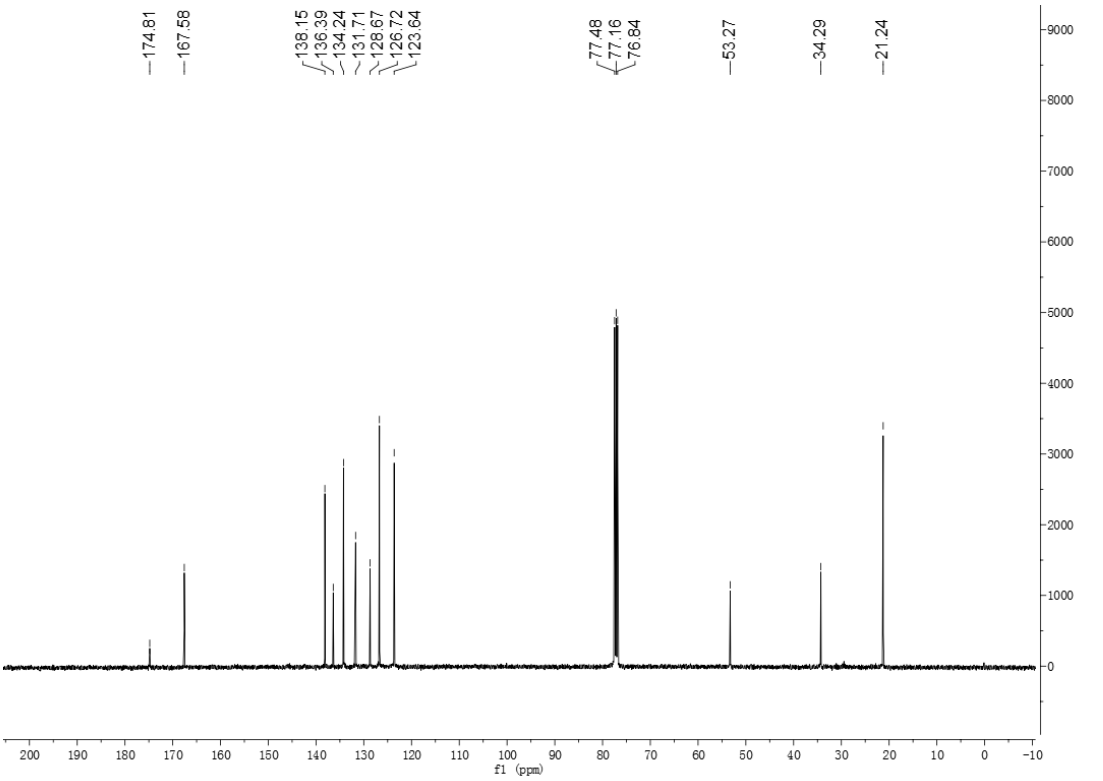

Supplementary Figure 23. ^{1}H and ^{13}C NMR spectra for 3v

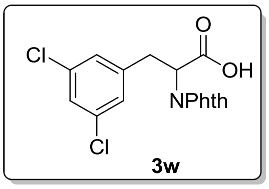

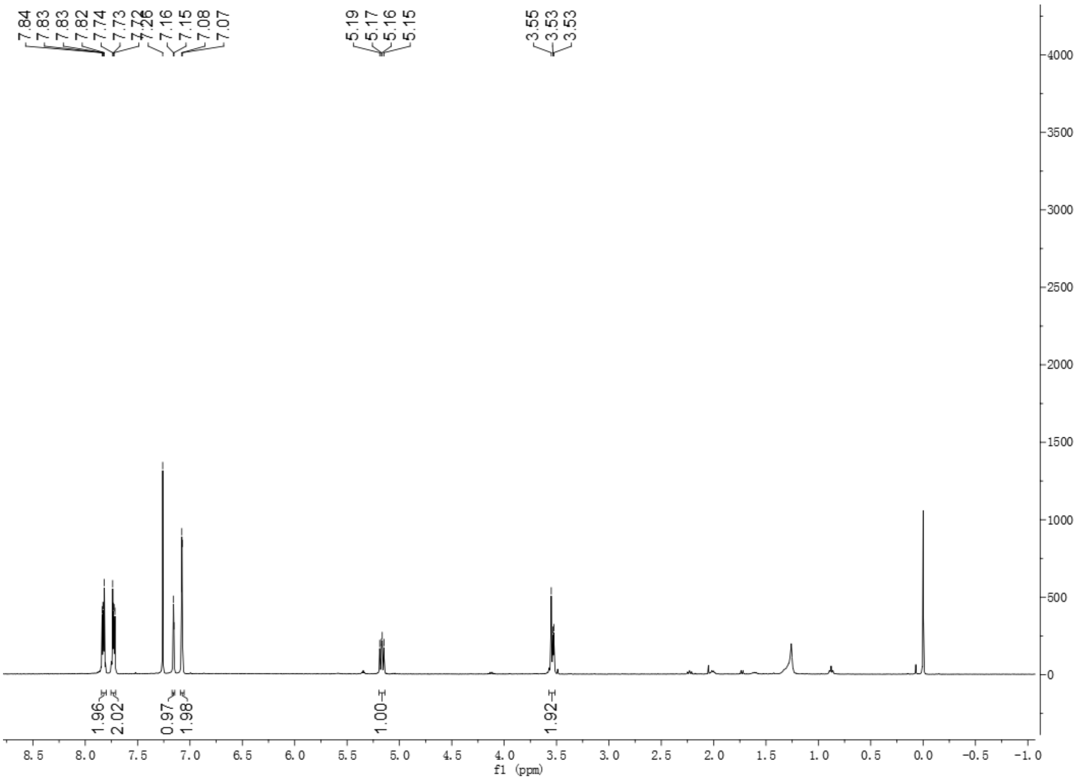

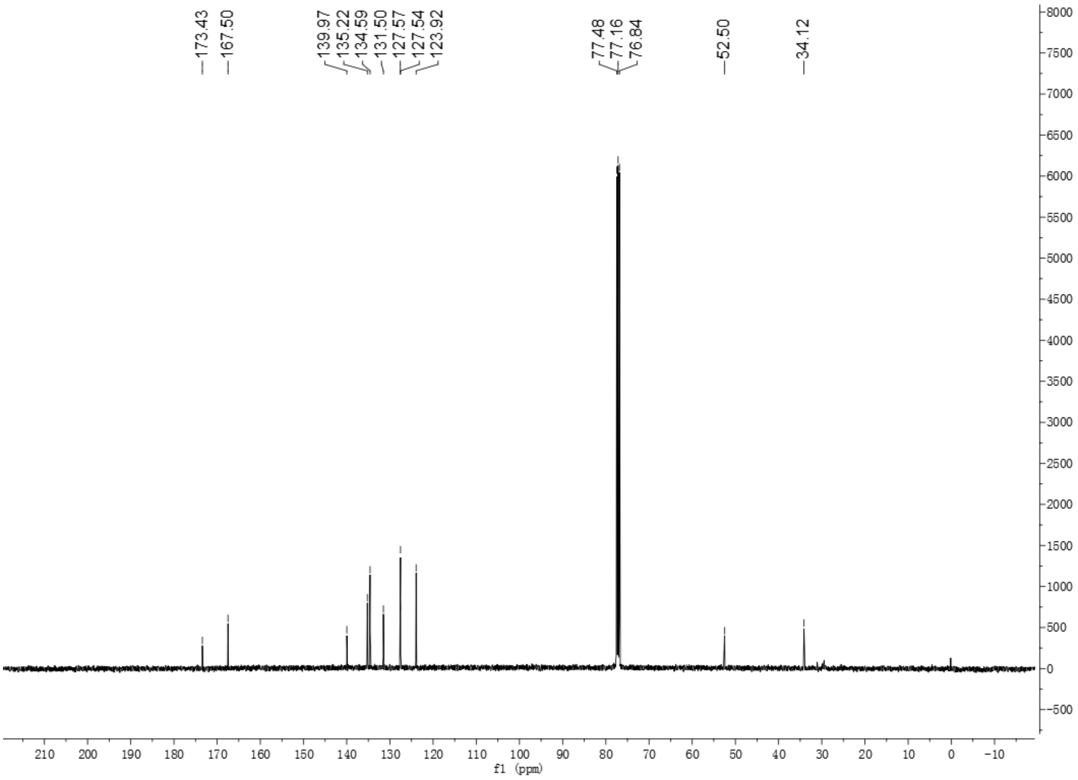

Supplementary Figure 24. ^{1}H and ^{13}C NMR spectra for 3w

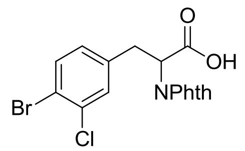

3x

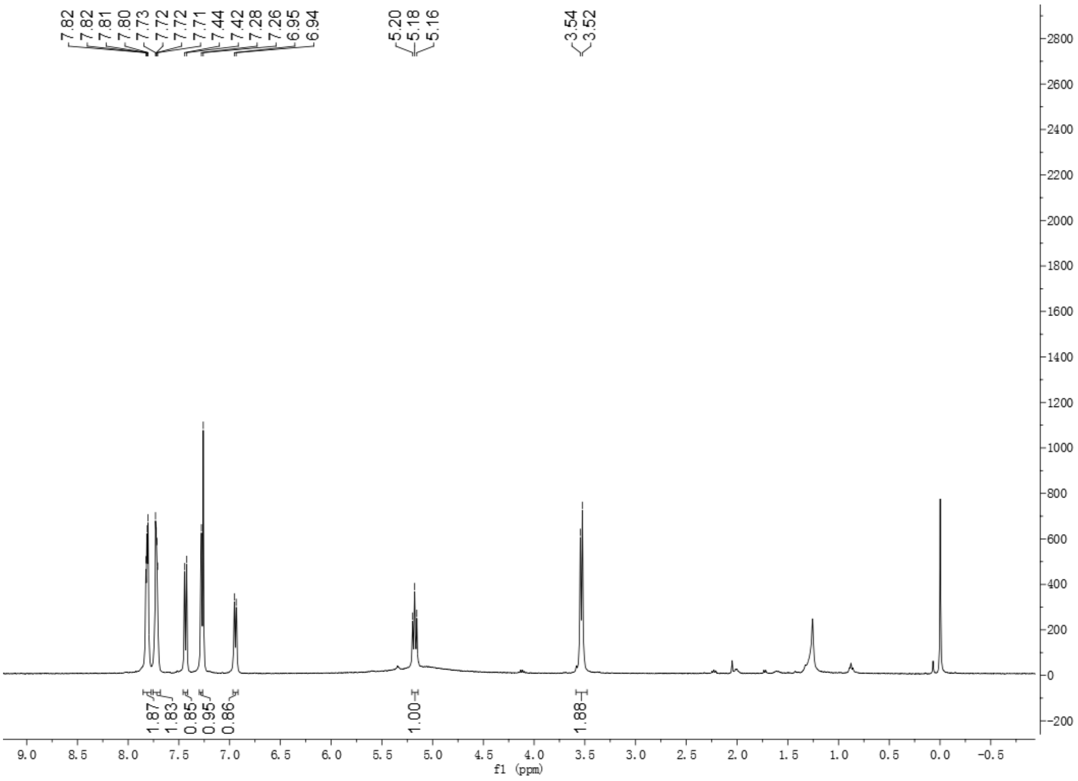

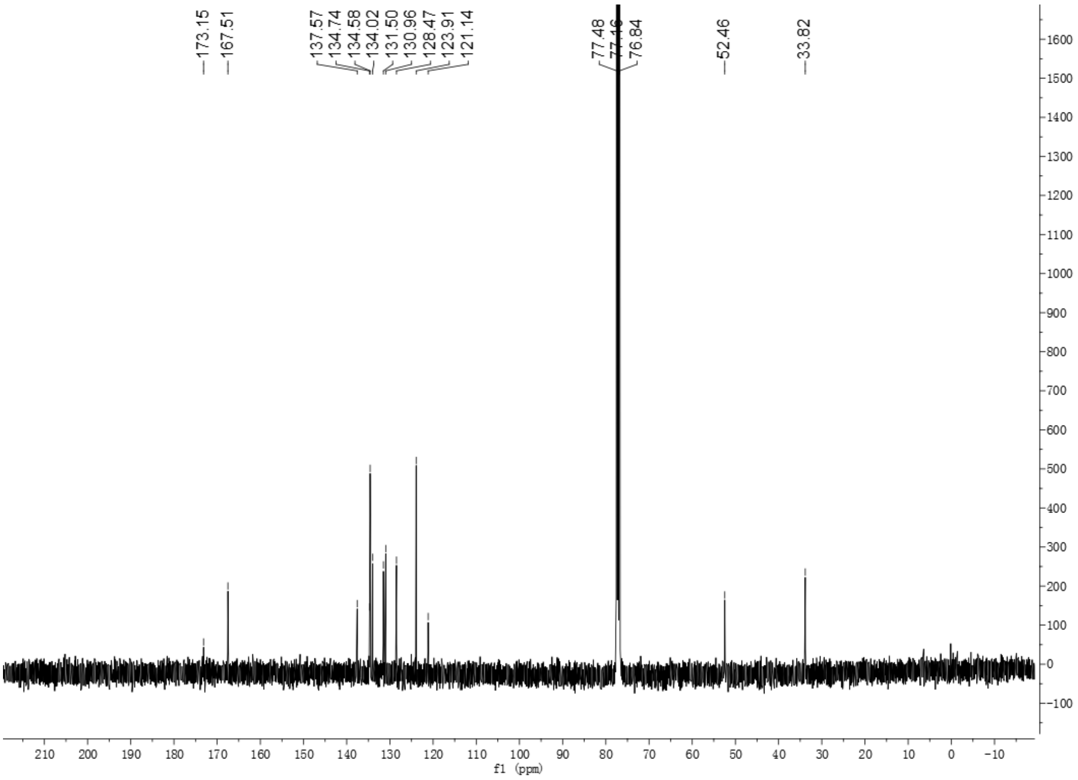

Supplementary Figure 25. ^{1}H and ^{13}C NMR spectra for 3x

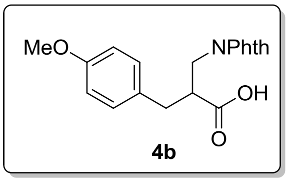

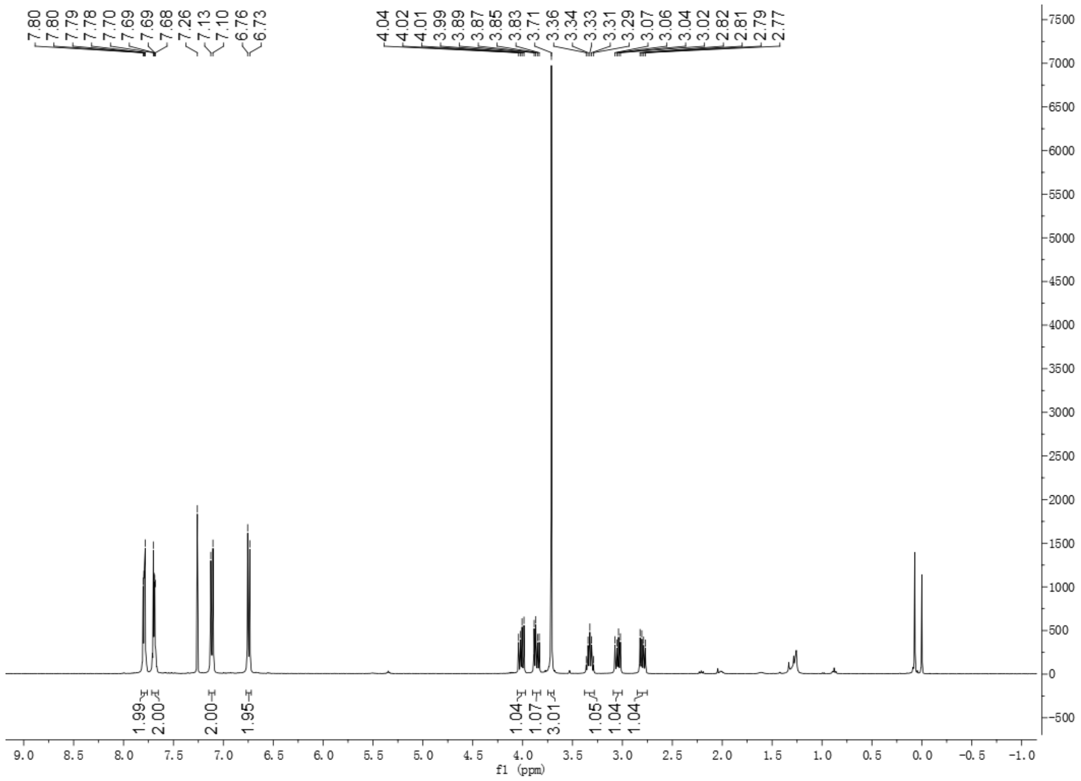

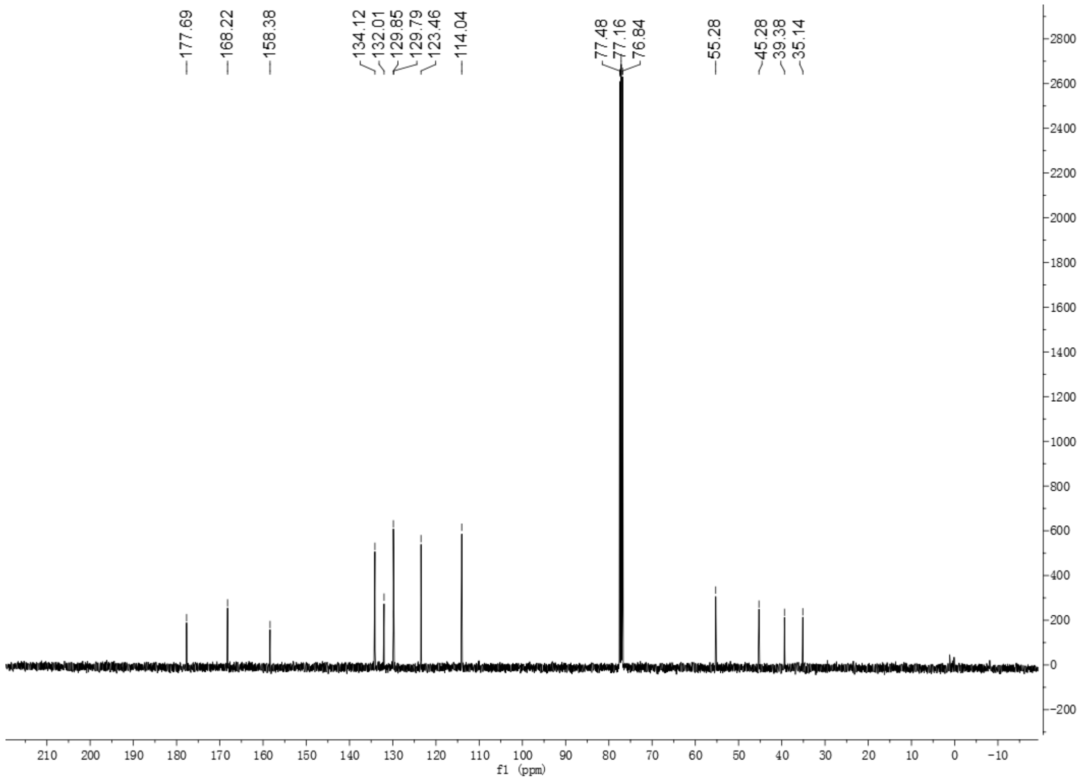

Supplementary Figure 26. ^{1}H and ^{13}C NMR spectra for 4b

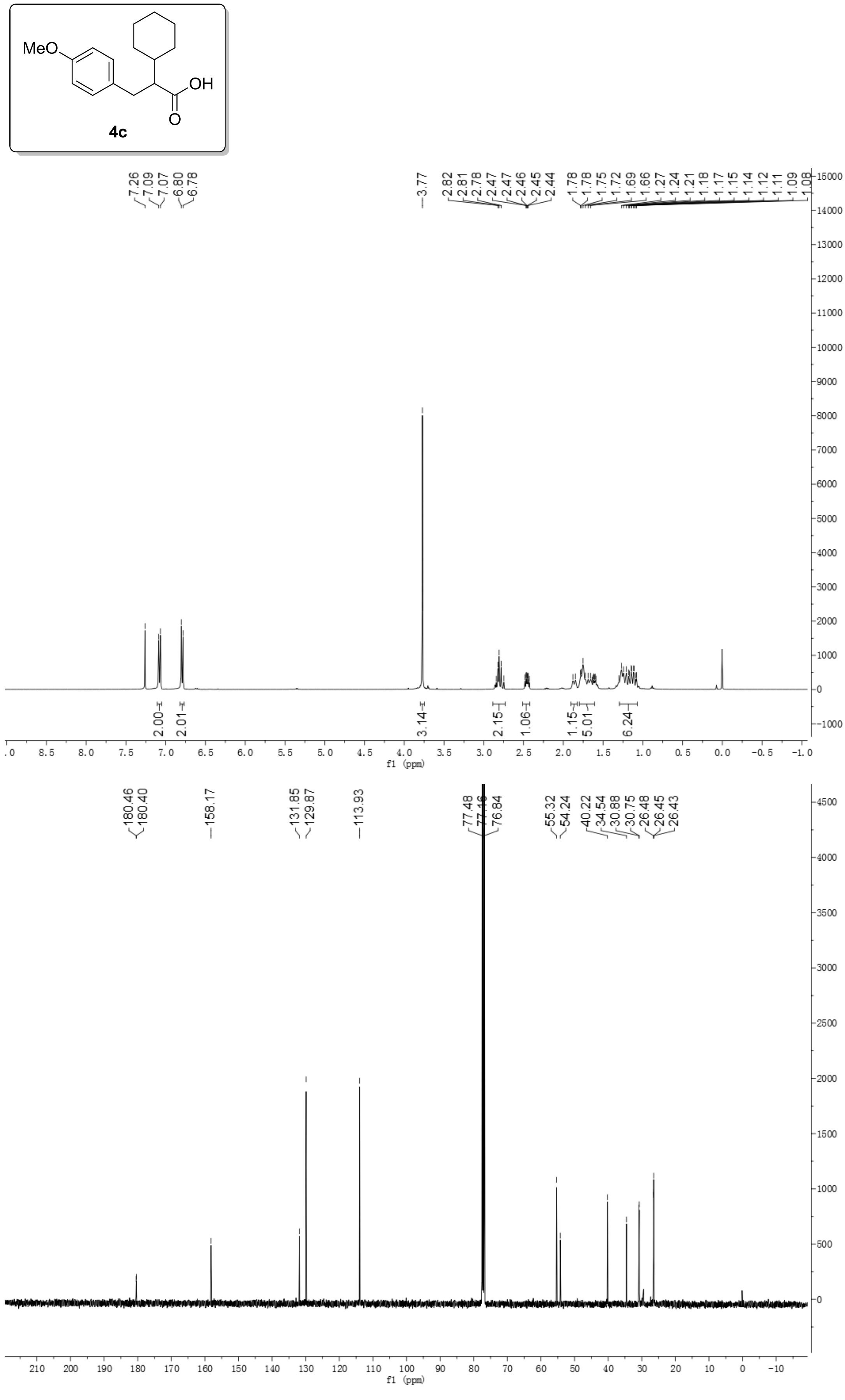

Supplementary Figure 27. ^{1}H and ^{13}C NMR spectra for 4c

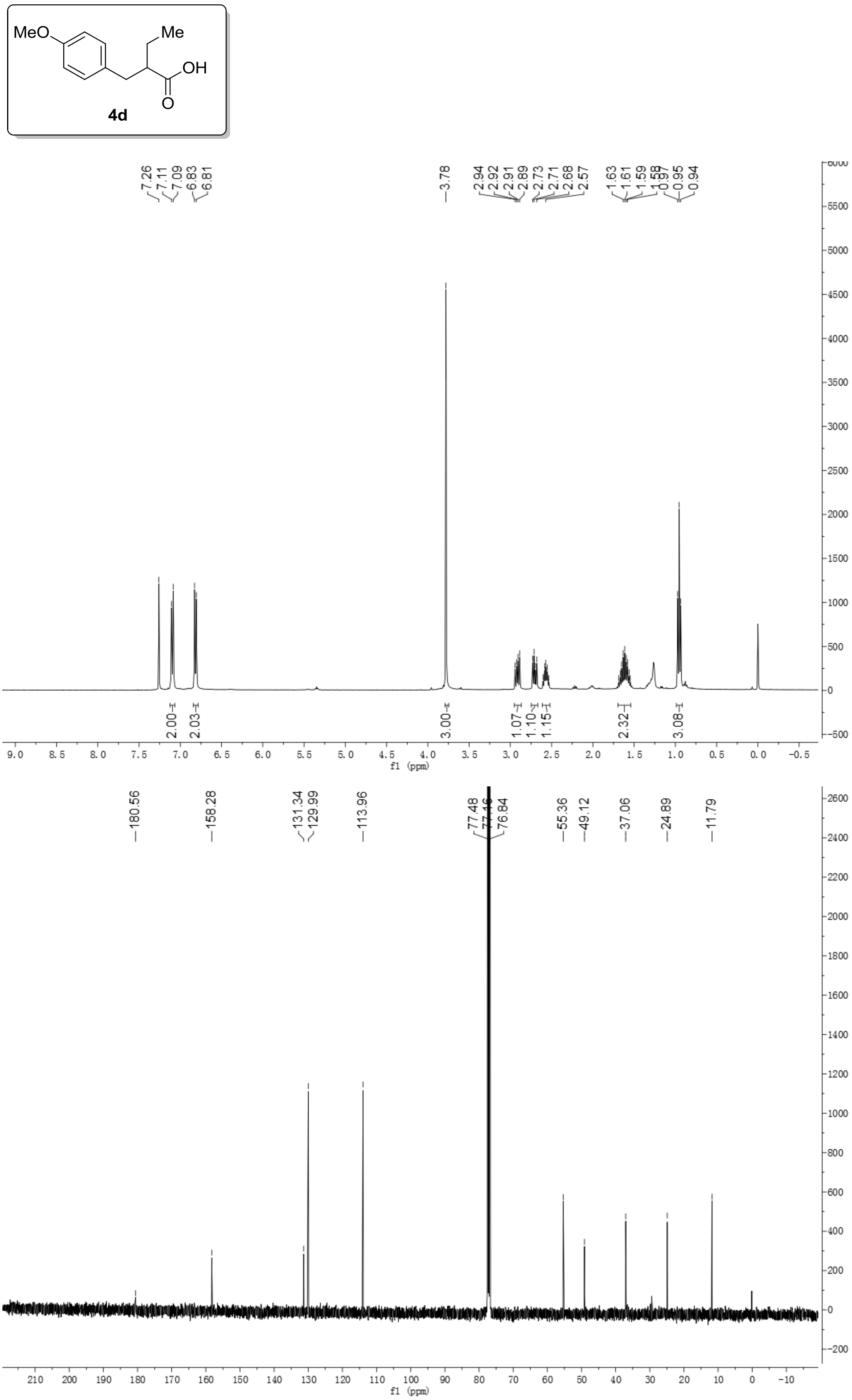

Supplementary Figure 28. ^{1}H and ^{13}C NMR spectra for 4d

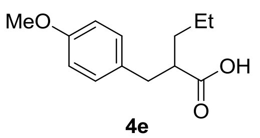

4e

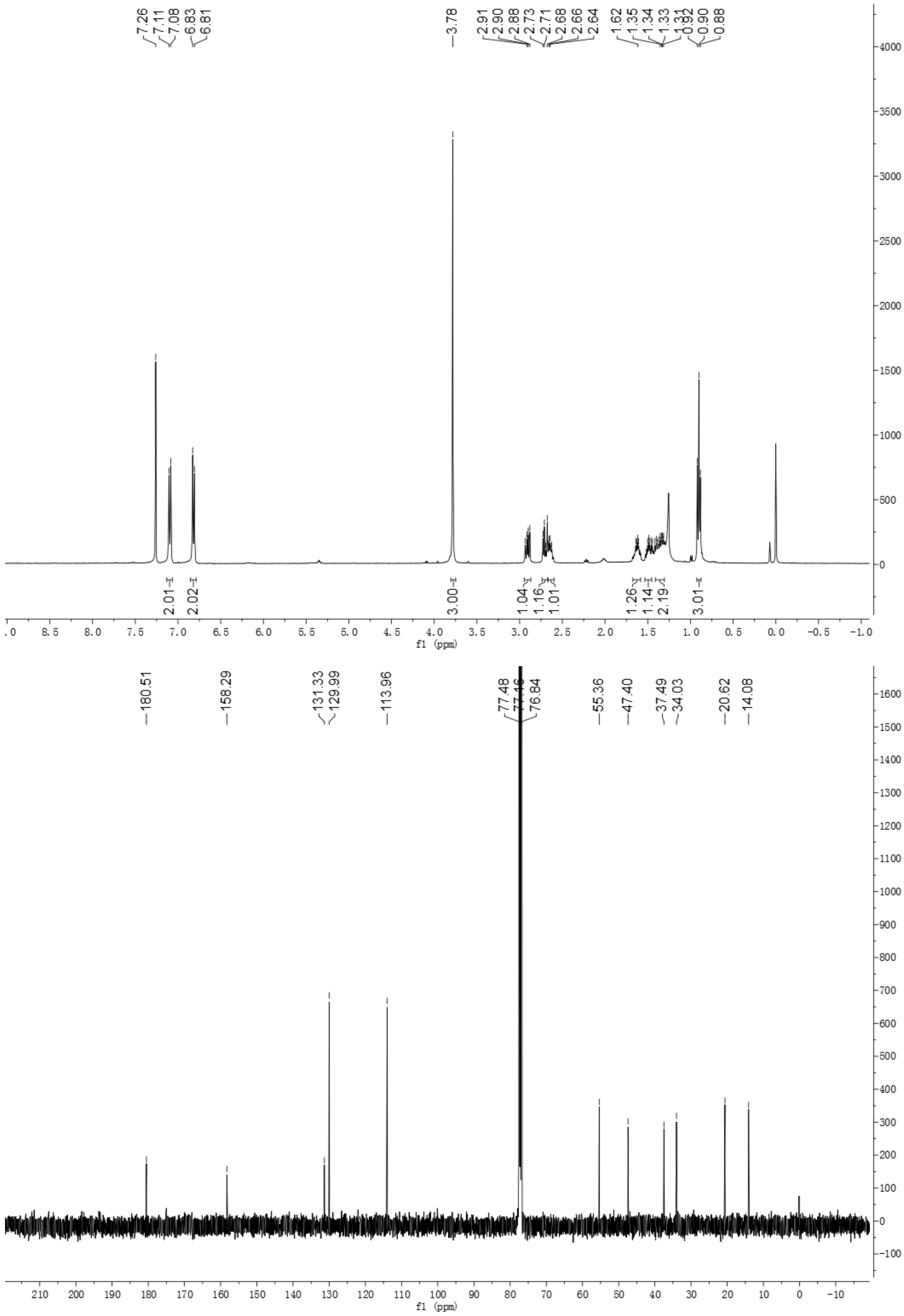

Supplementary Figure 29. ^{1}**H and** ^{13}**C NMR spectra for 4e**

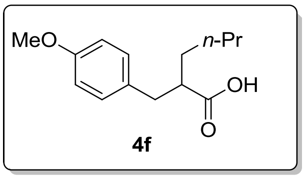

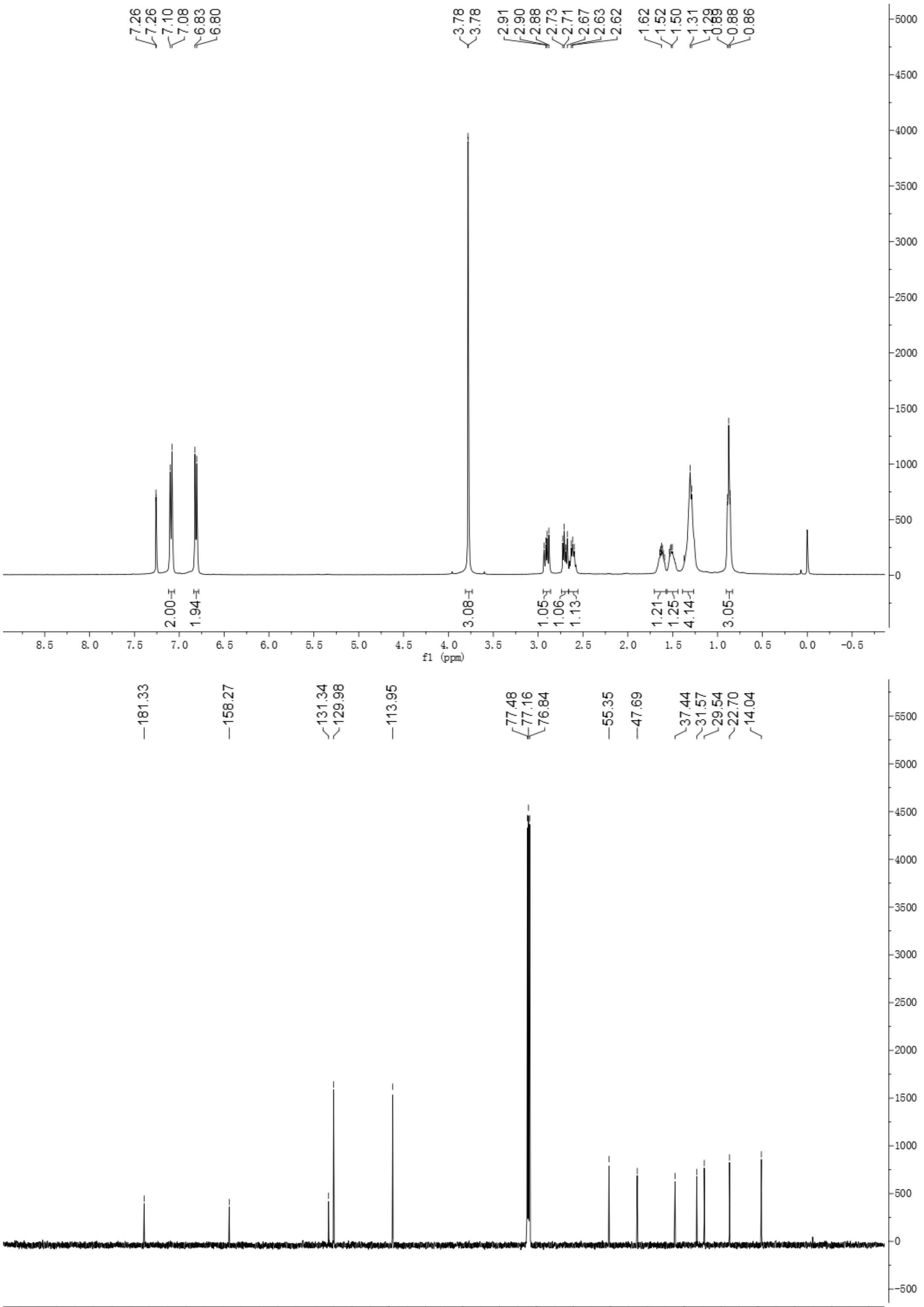

Supplementary Figure 30. ^{1}H and ^{13}C NMR spectra for 4f

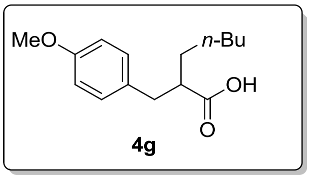

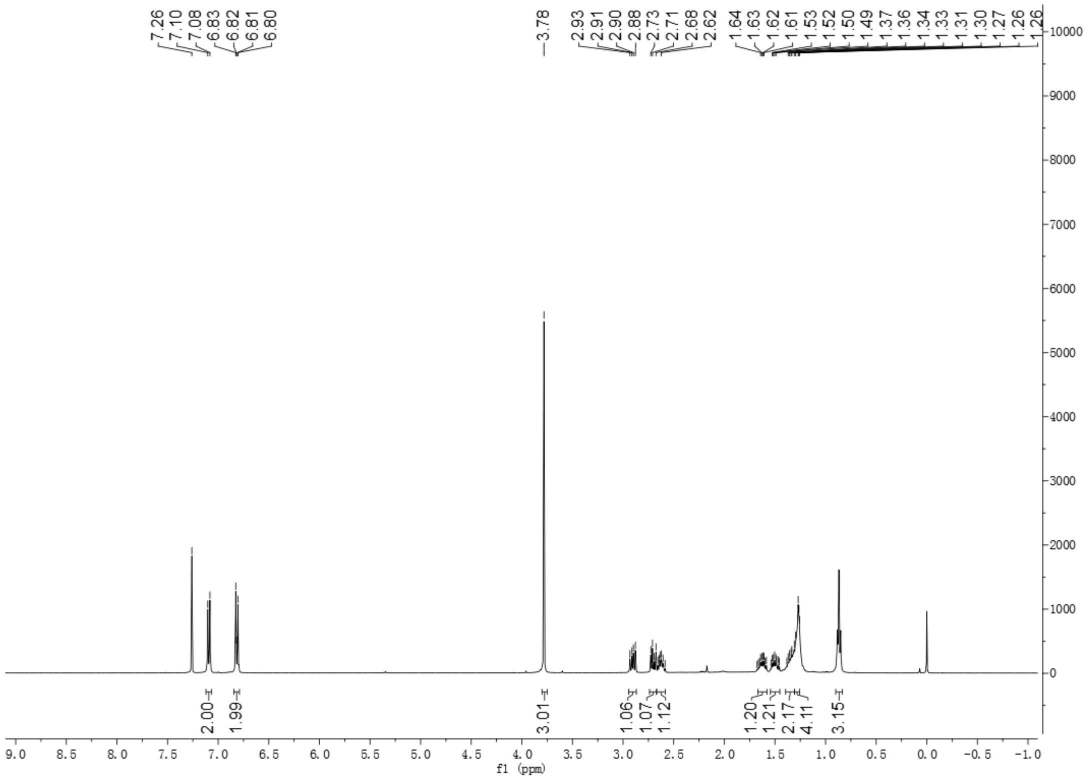

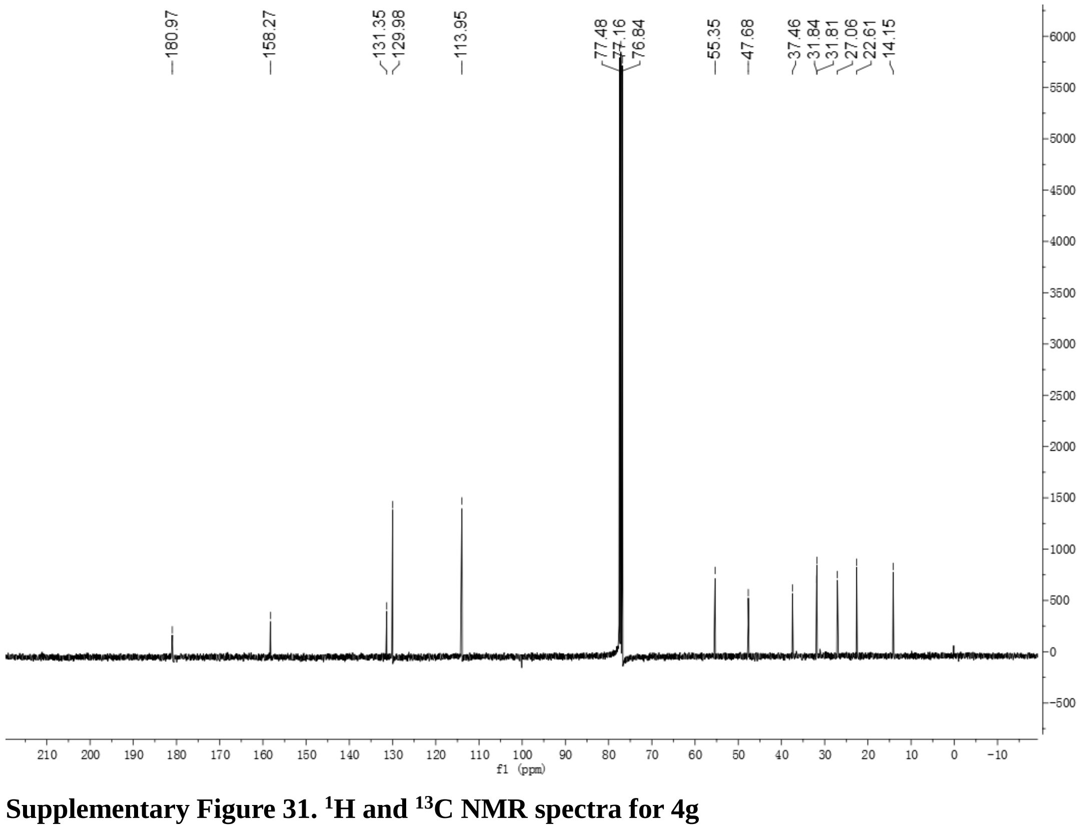

Supplementary Figure 31. ^{1}H and ^{13}C NMR spectra for 4g

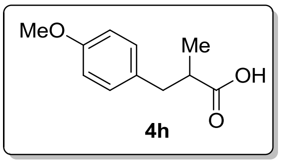

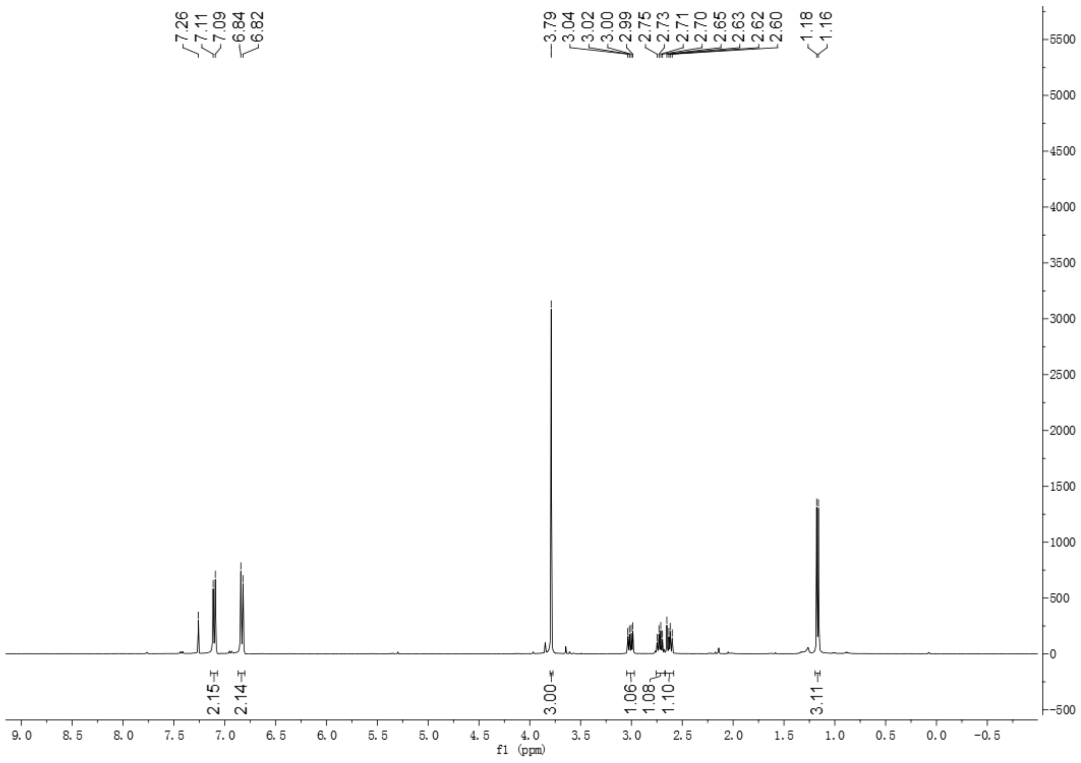

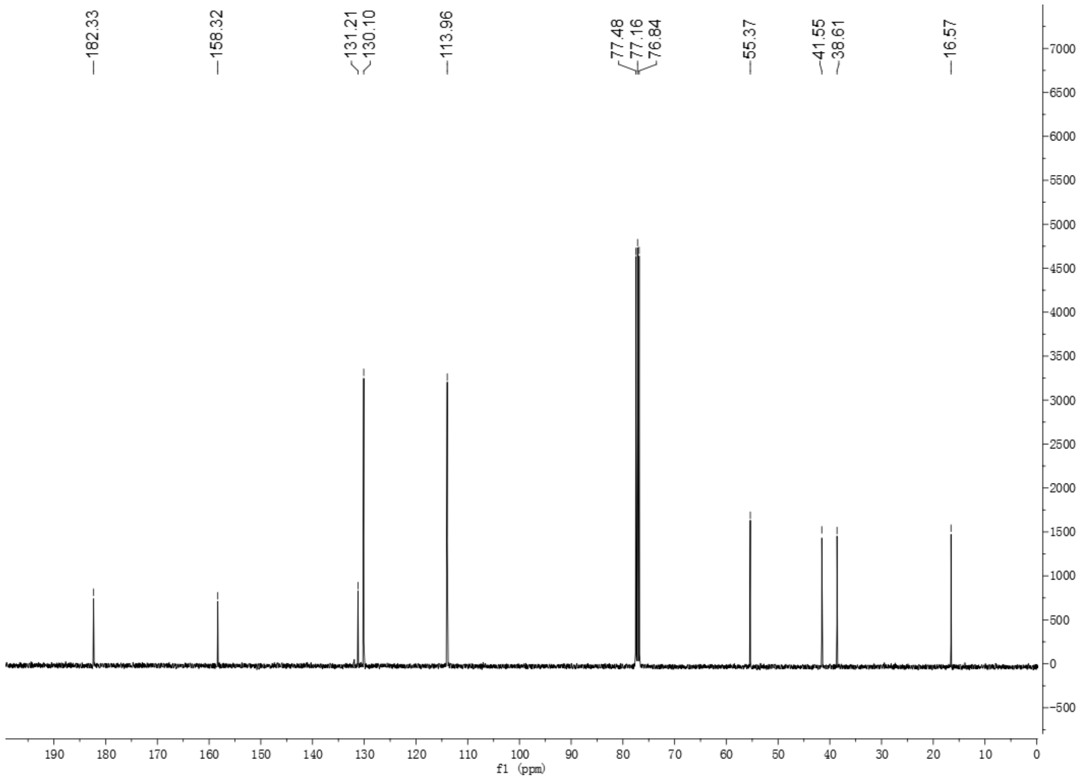

Supplementary Figure 32. ^{1}H and ^{13}C NMR spectra for 4h

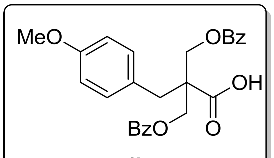

4i

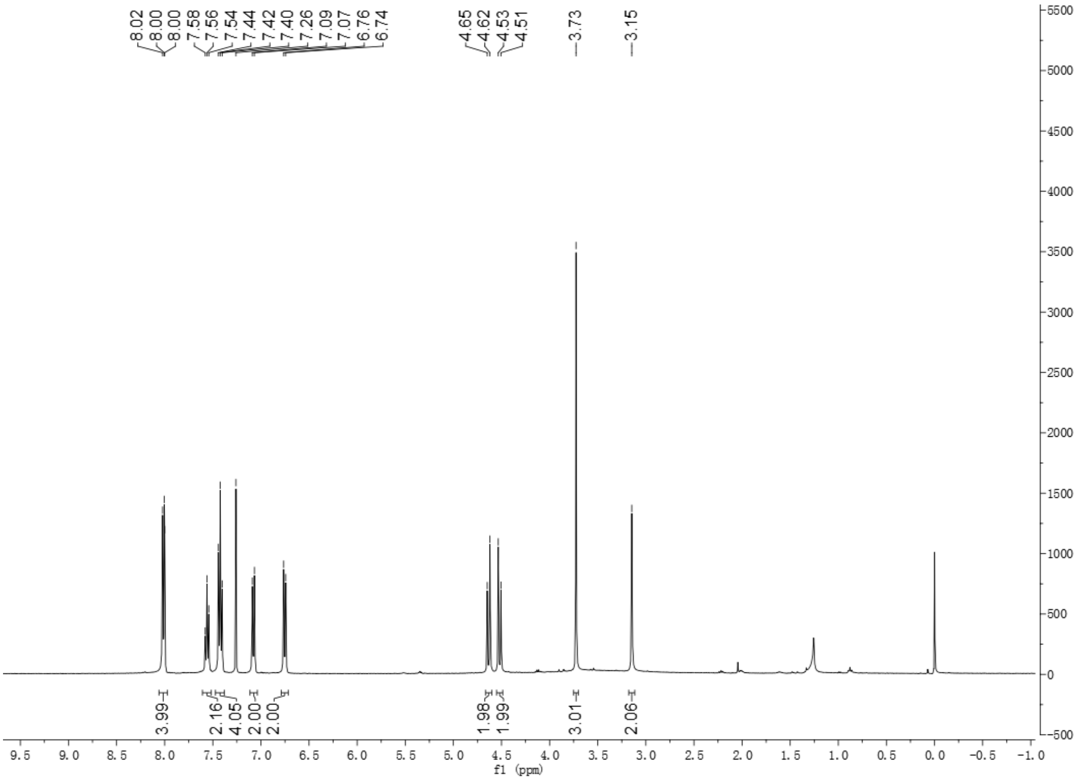

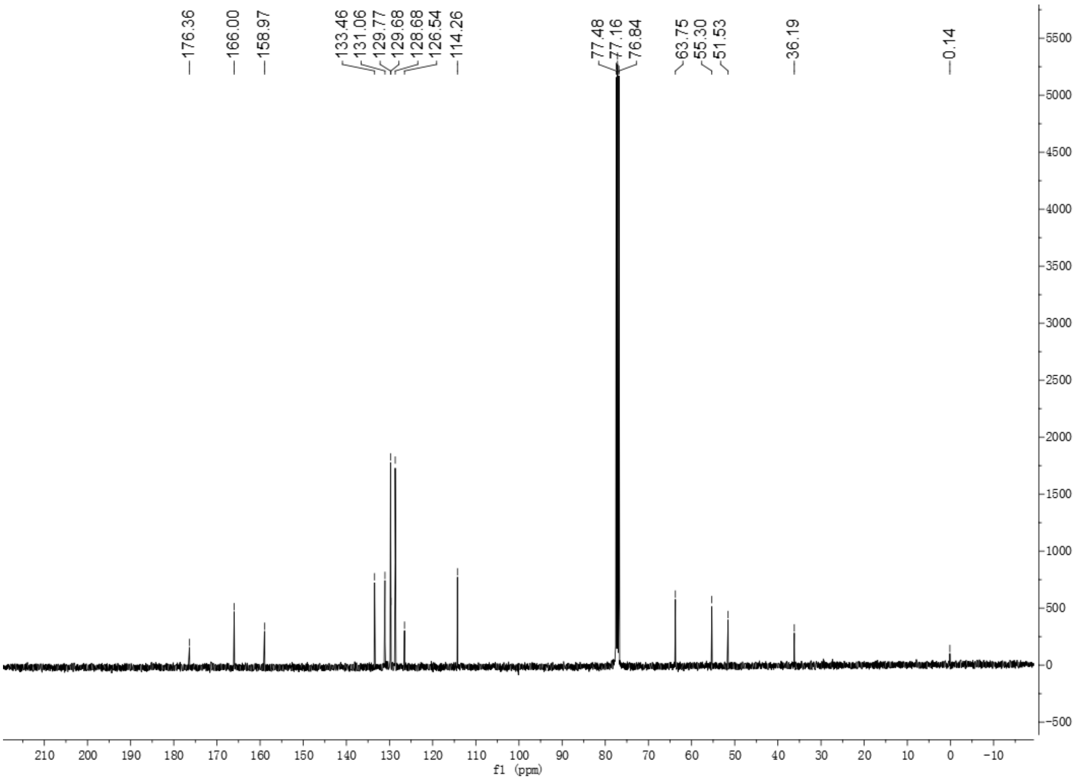

Supplementary Figure 33. ^{1}H and ^{13}C NMR spectra for 4i

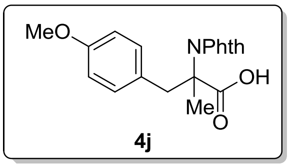

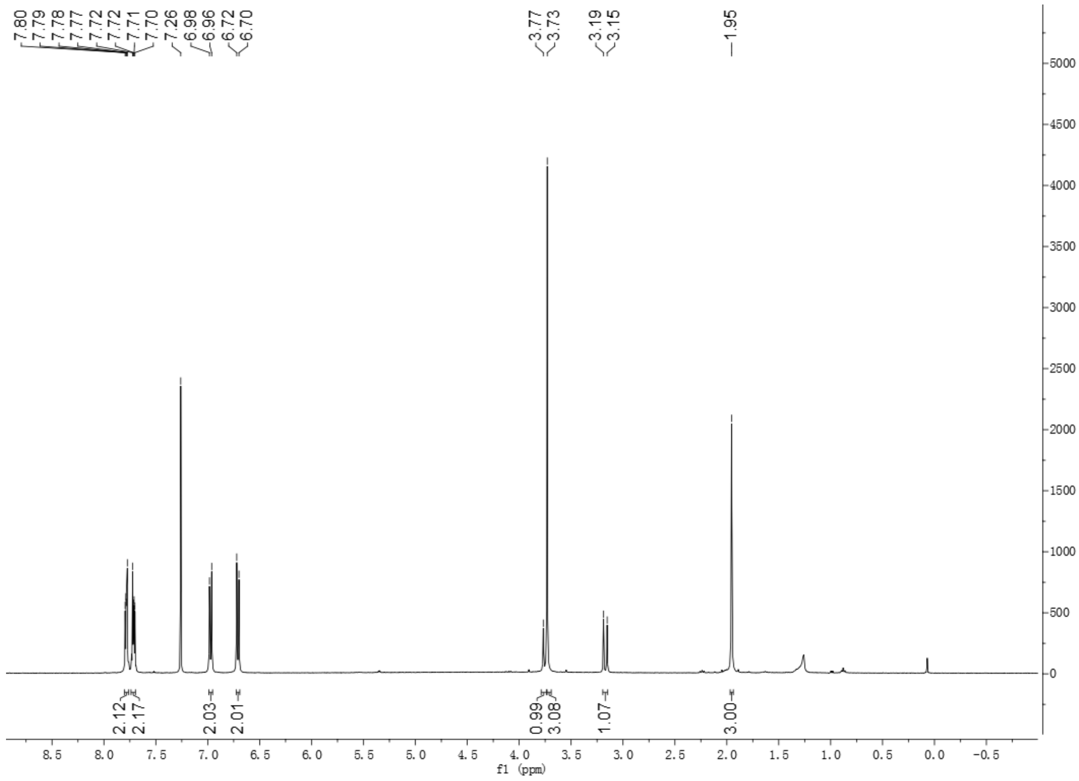

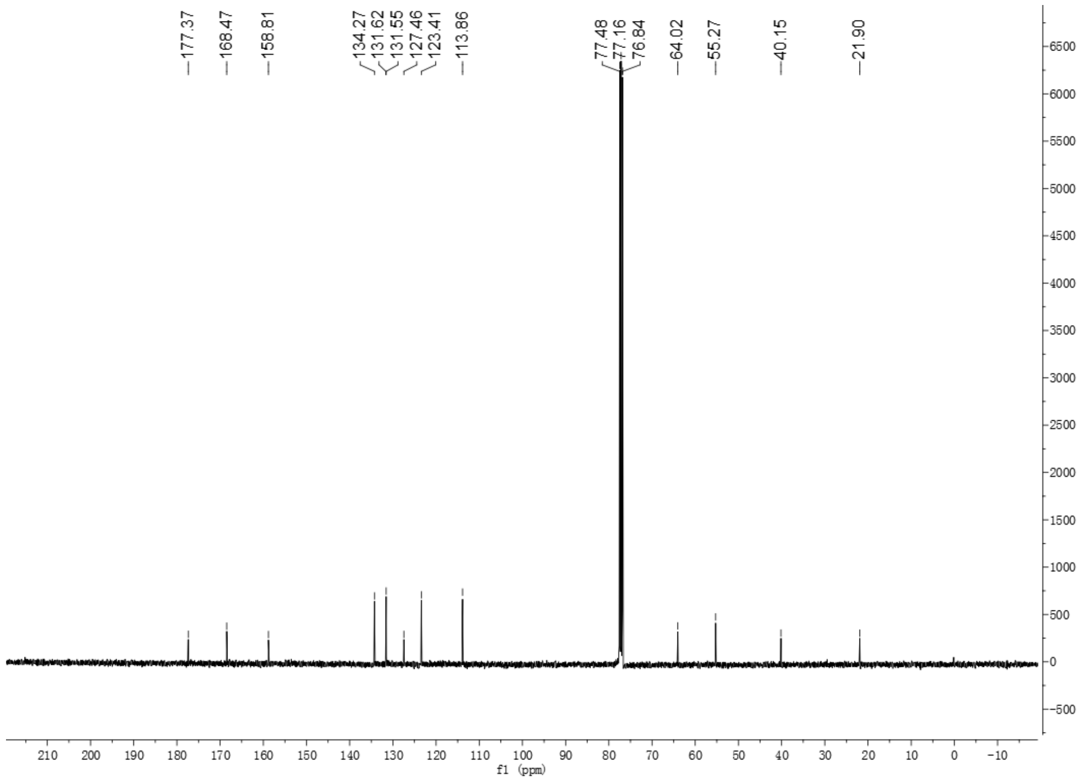

Supplementary Figure 34. ^{1}H and ^{13}C NMR spectra for 4j

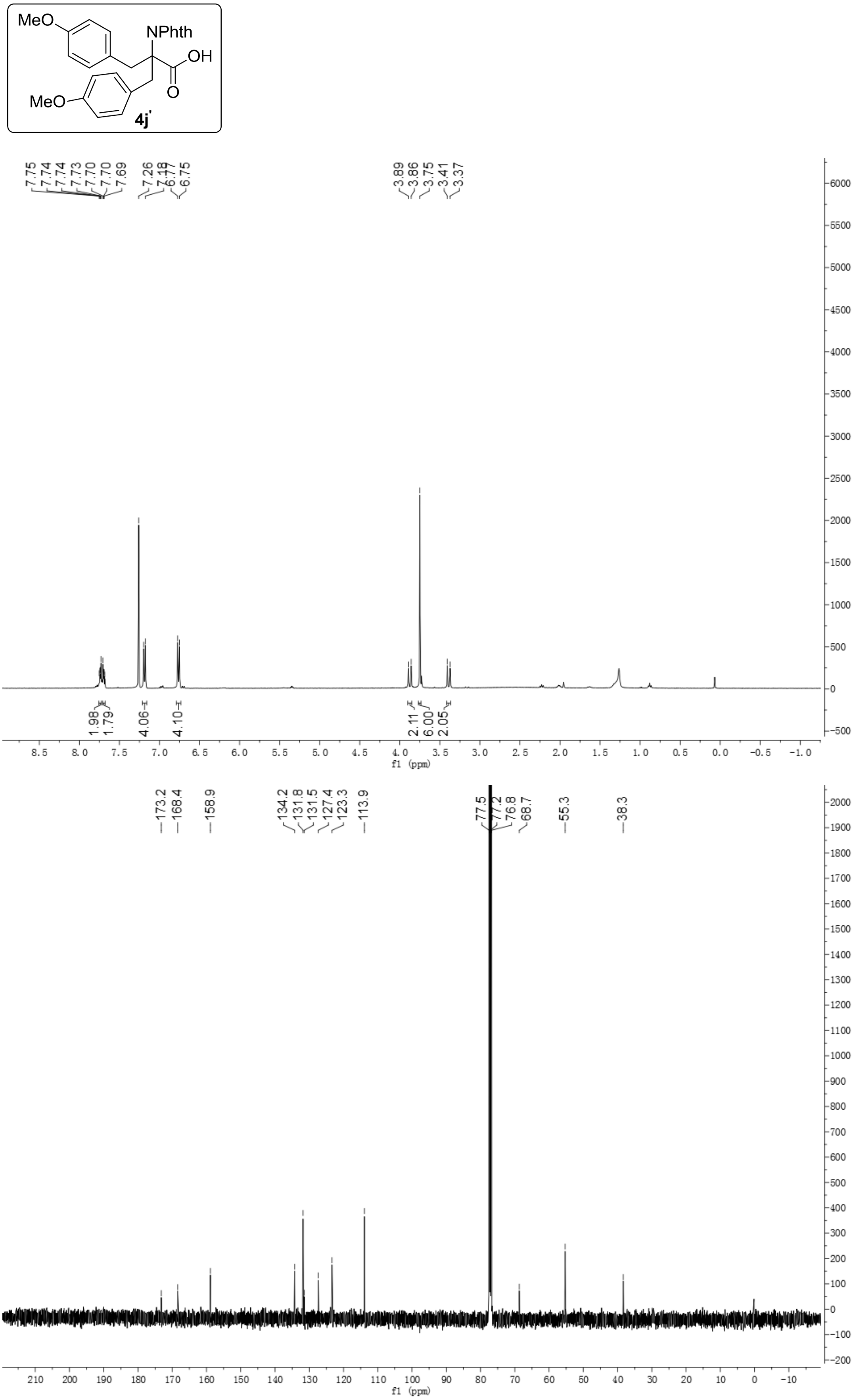

Supplementary Figure 35. ^{1}H and ^{13}C NMR spectra for 4j'

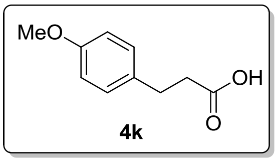

4k

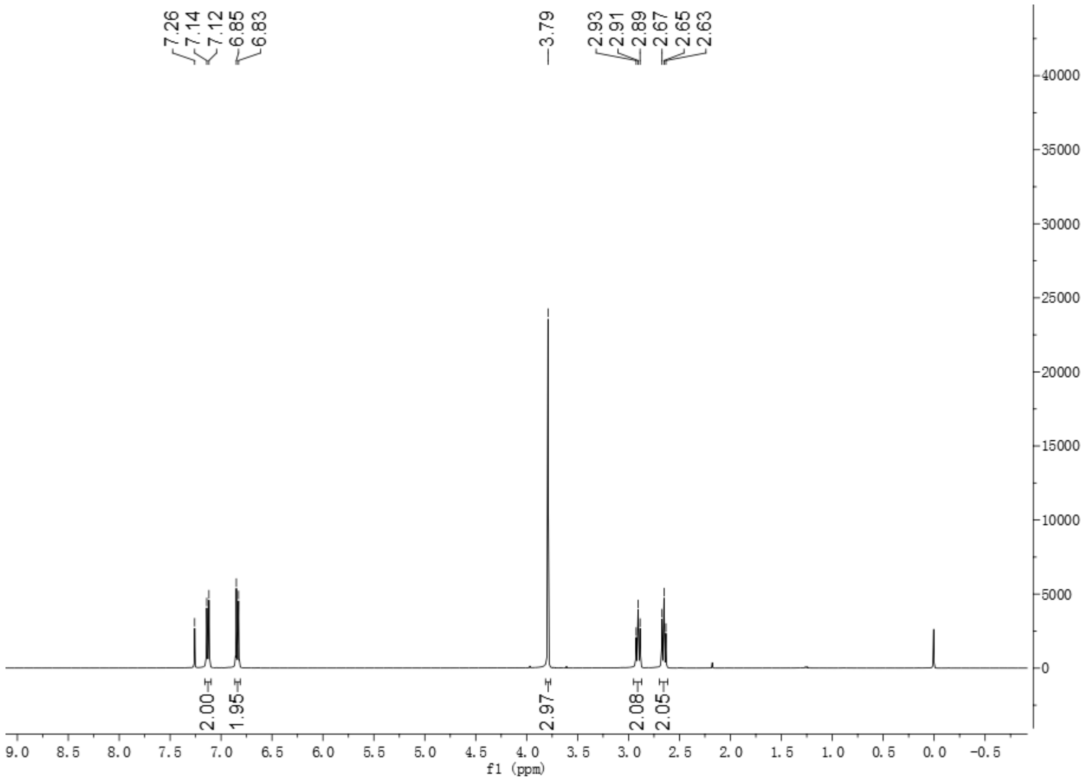

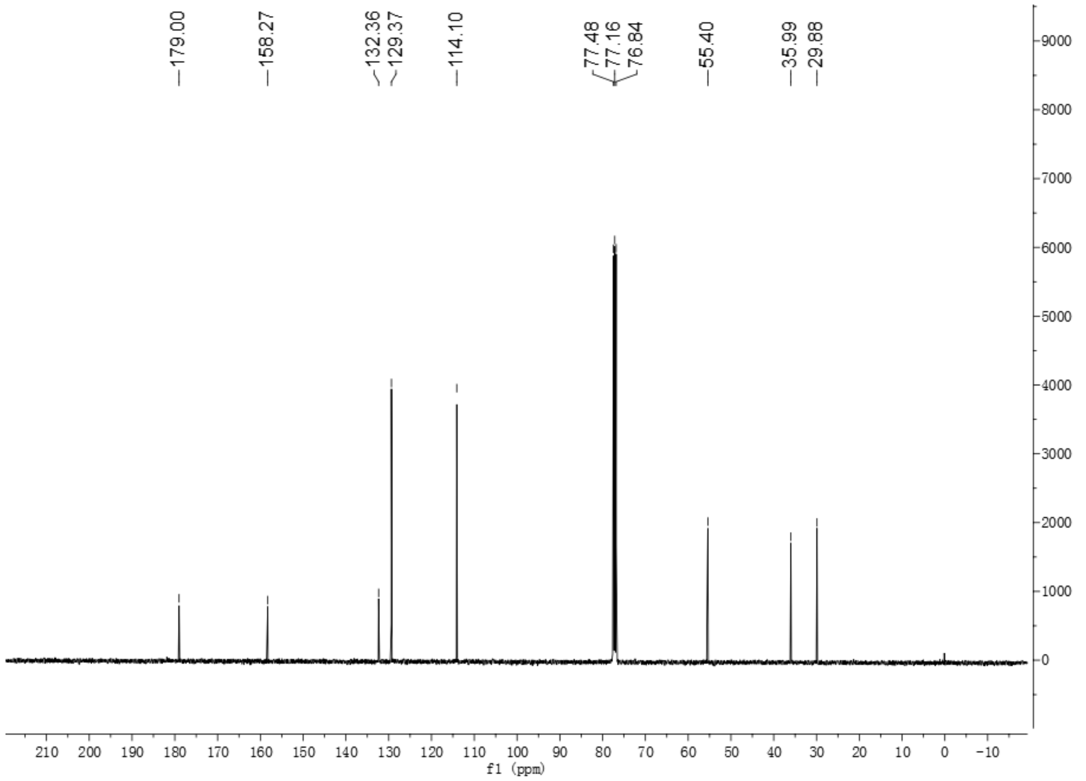

Supplementary Figure 36. ^{1}H and ^{13}C NMR spectra for 4k

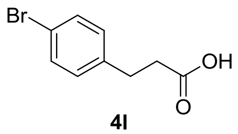

4l

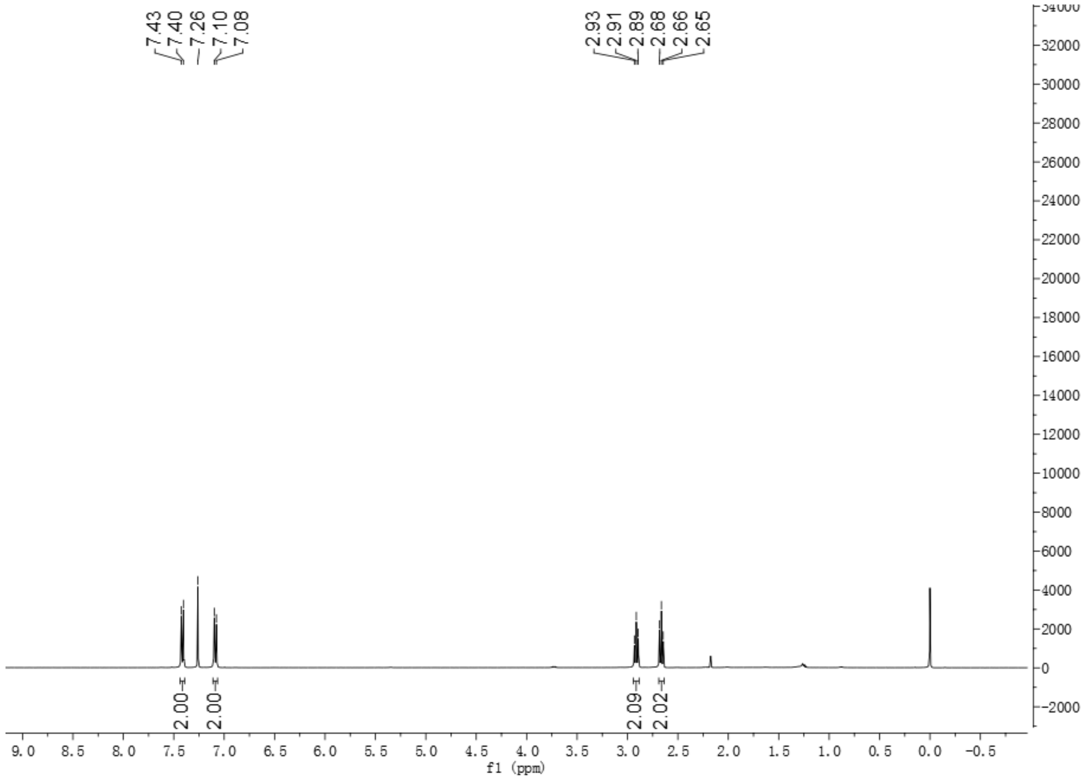

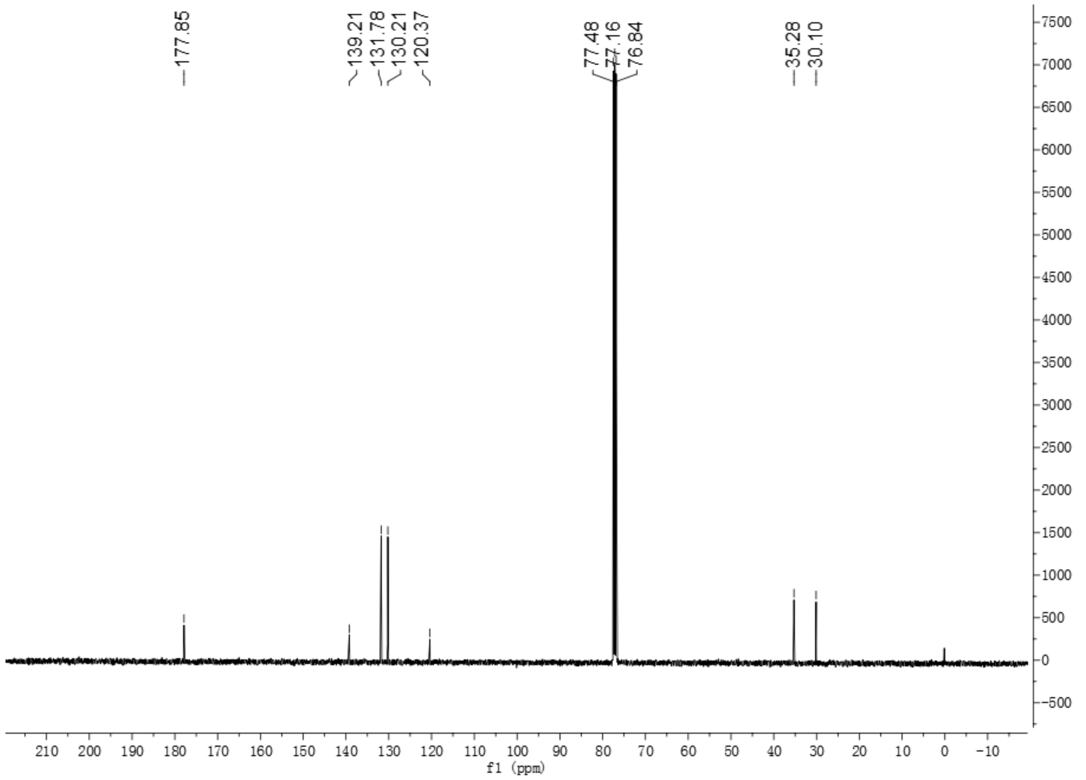

Supplementary Figure 37. ^{1}H and ^{13}C NMR spectra for 4l

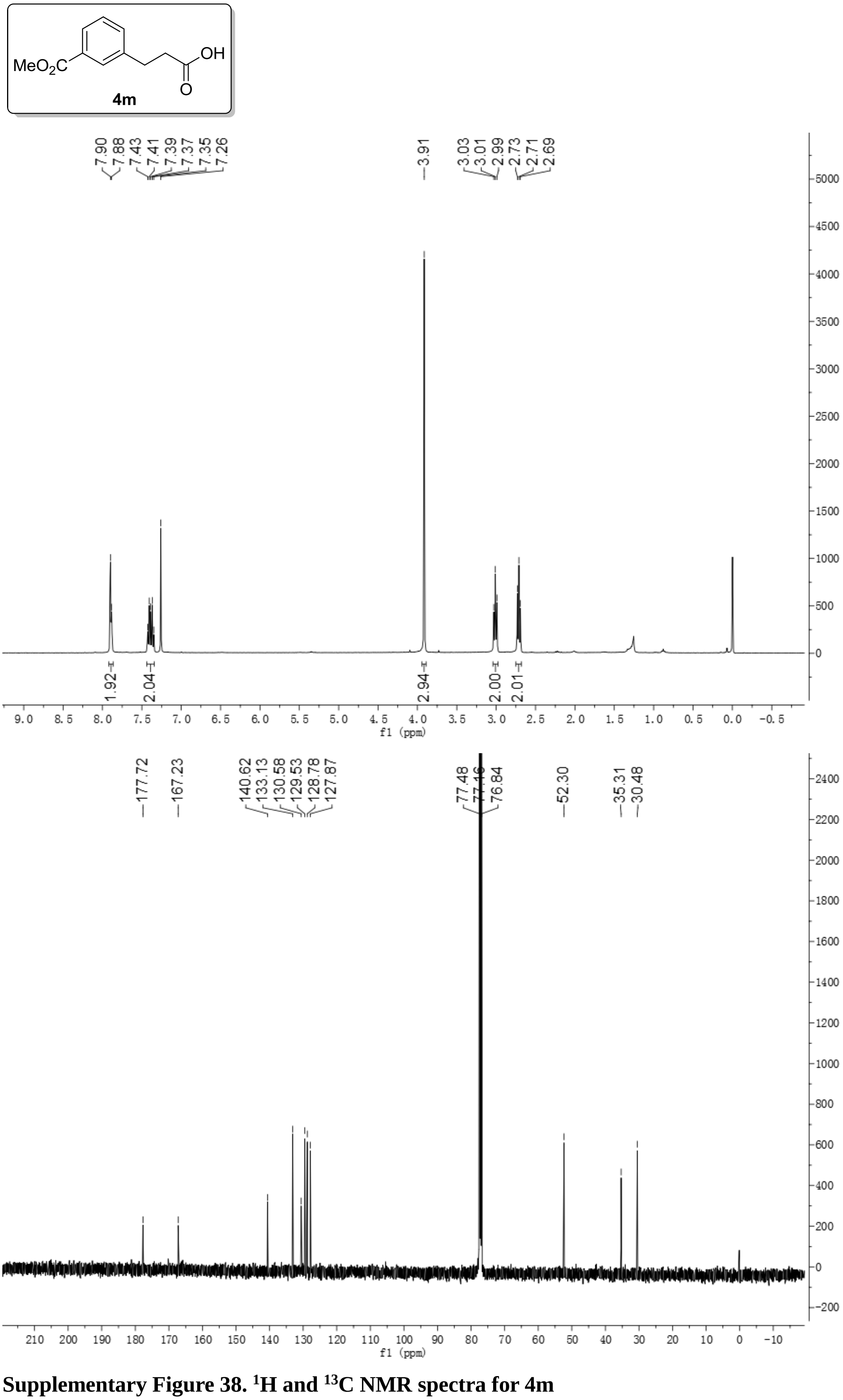

Supplementary Figure 38. ^{1}H and ^{13}C NMR spectra for 4m

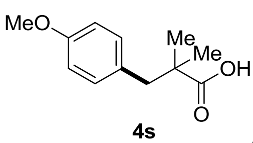

4s

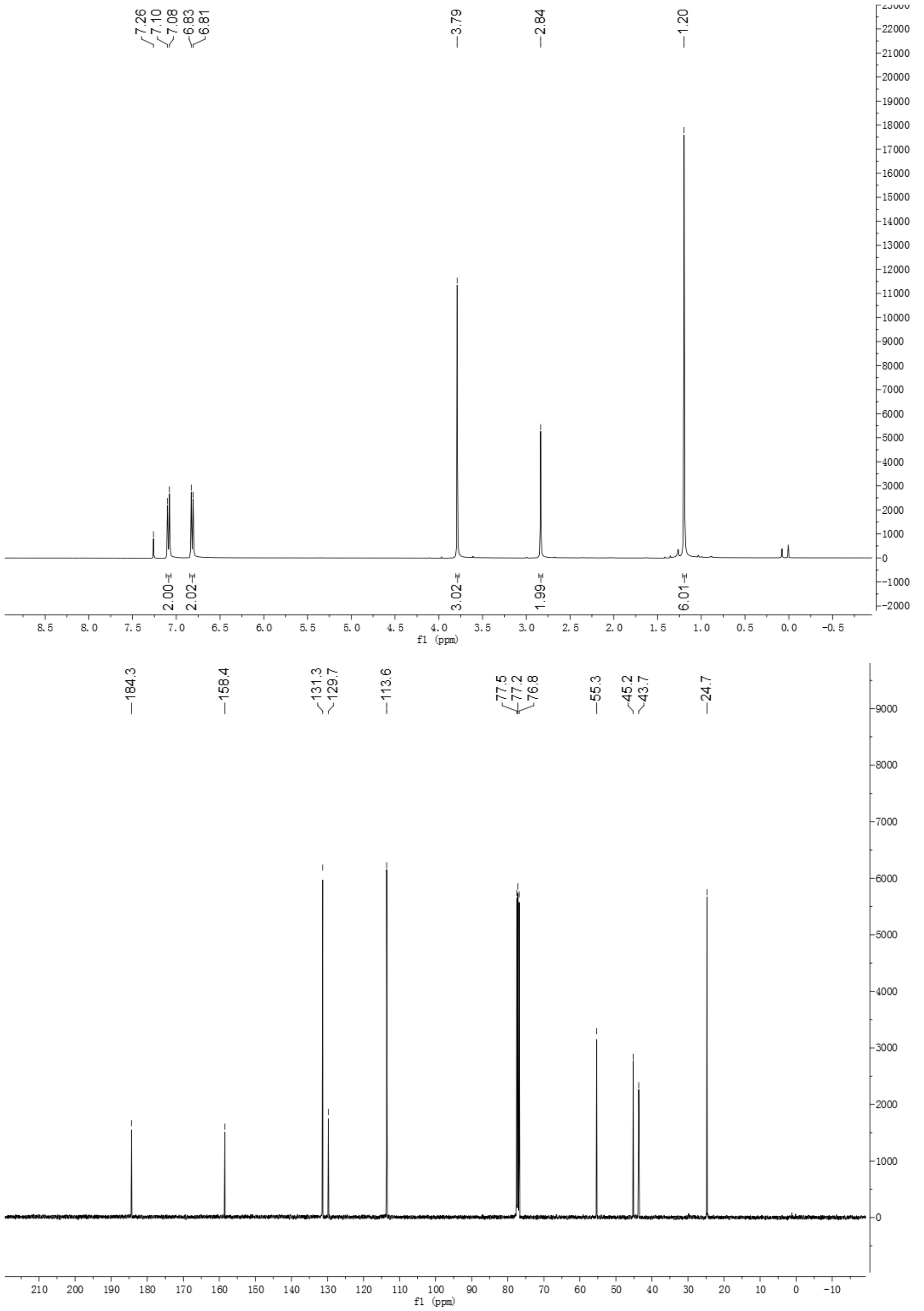

Supplementary Figure 39. ^{1}H and ^{13}C NMR spectra for 4s

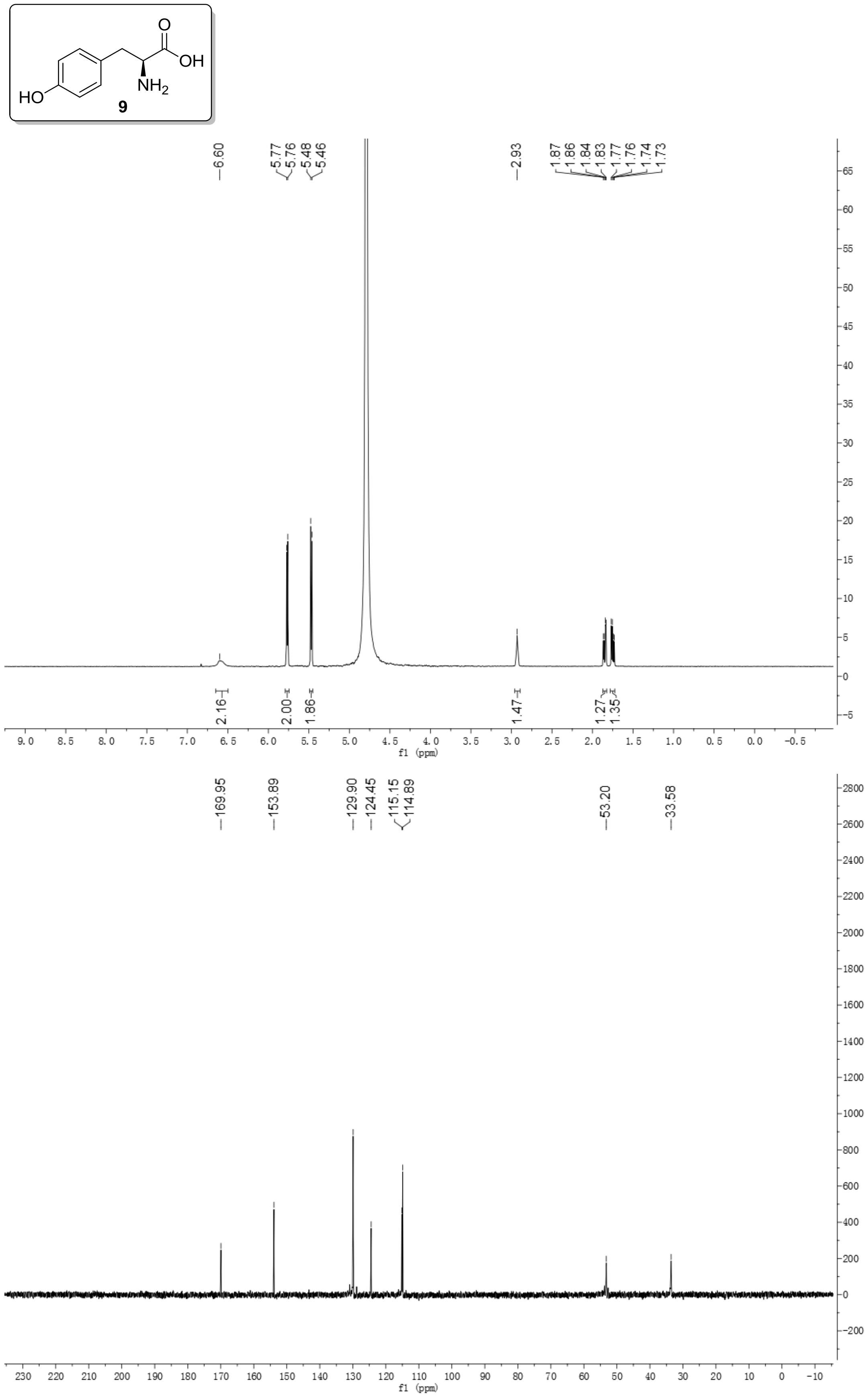

Supplementary Figure 40. ^{1}H and ^{13}C NMR spectra for 9

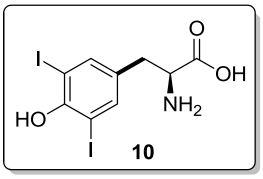

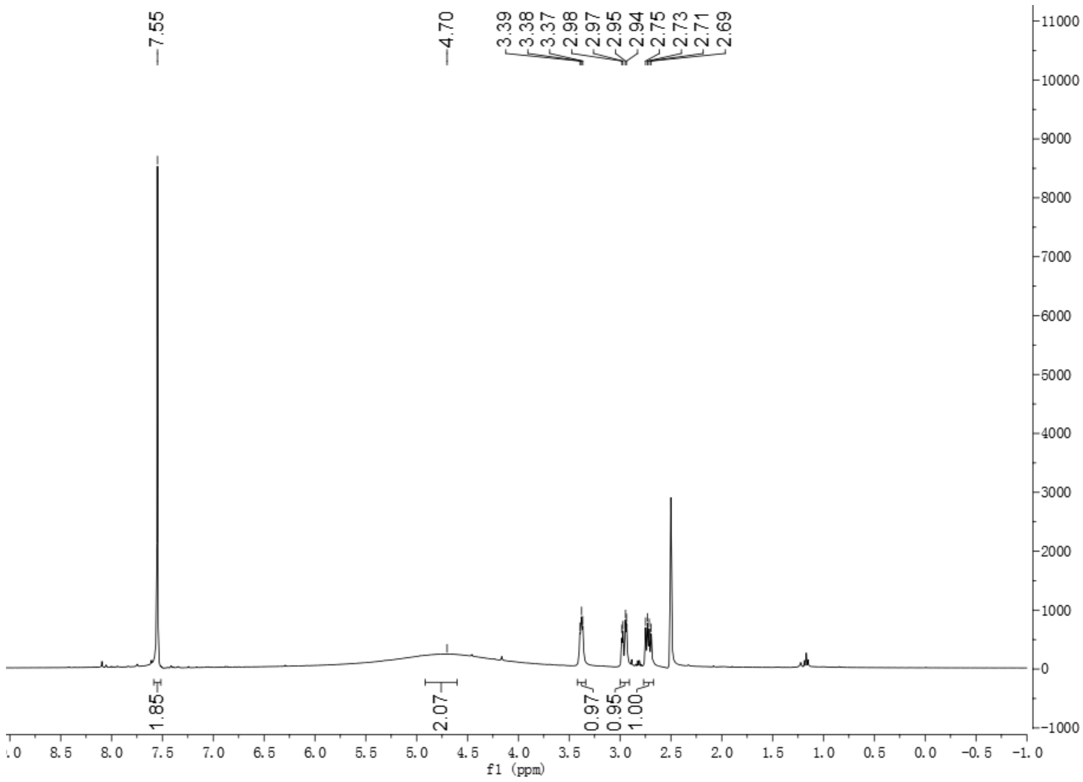

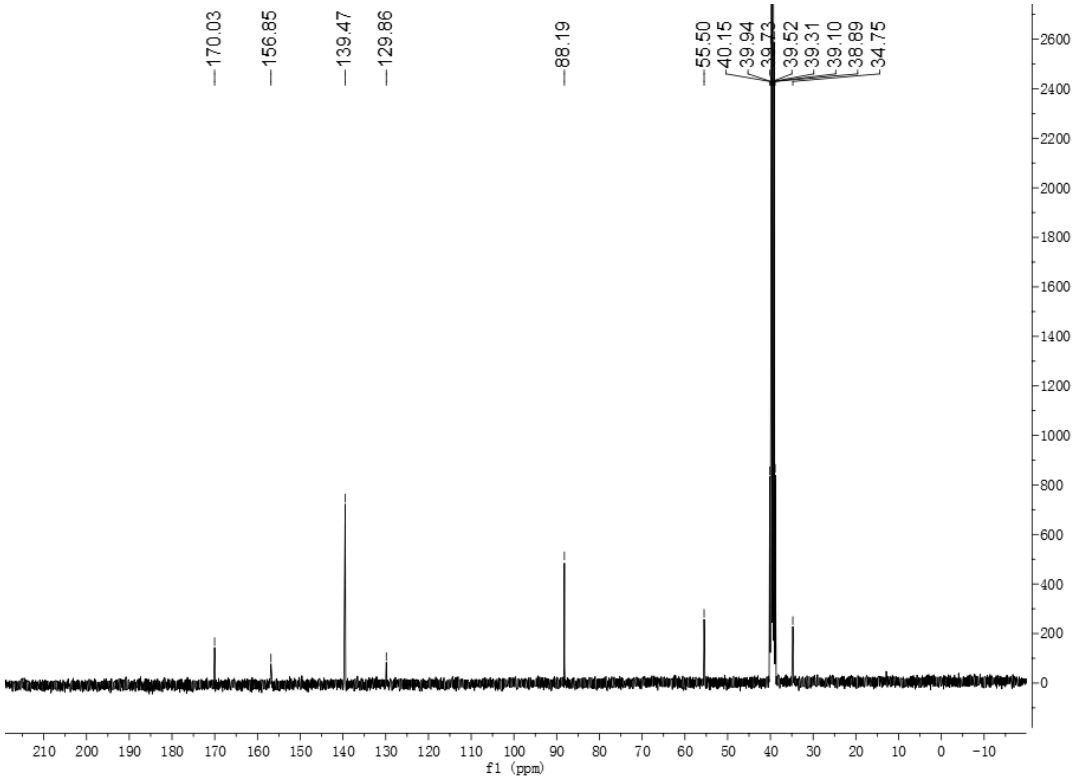

Supplementary Figure 41. ^{1}H and ^{13}C NMR spectra for 10

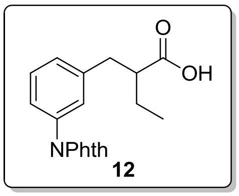

12

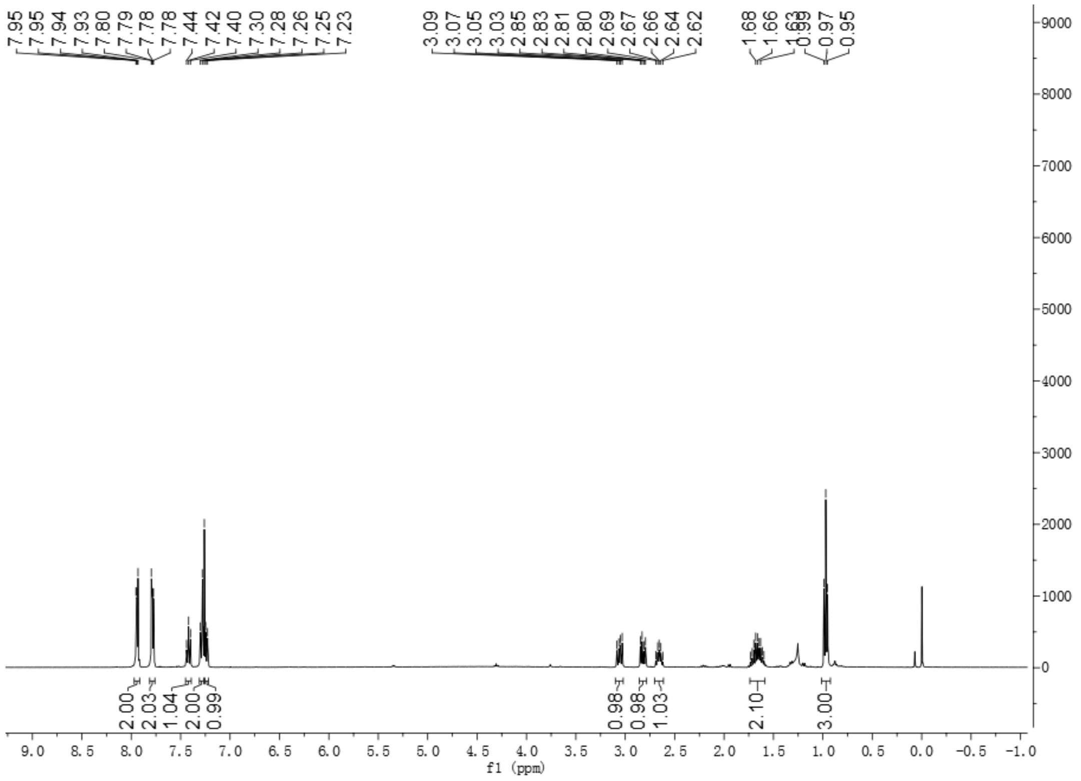

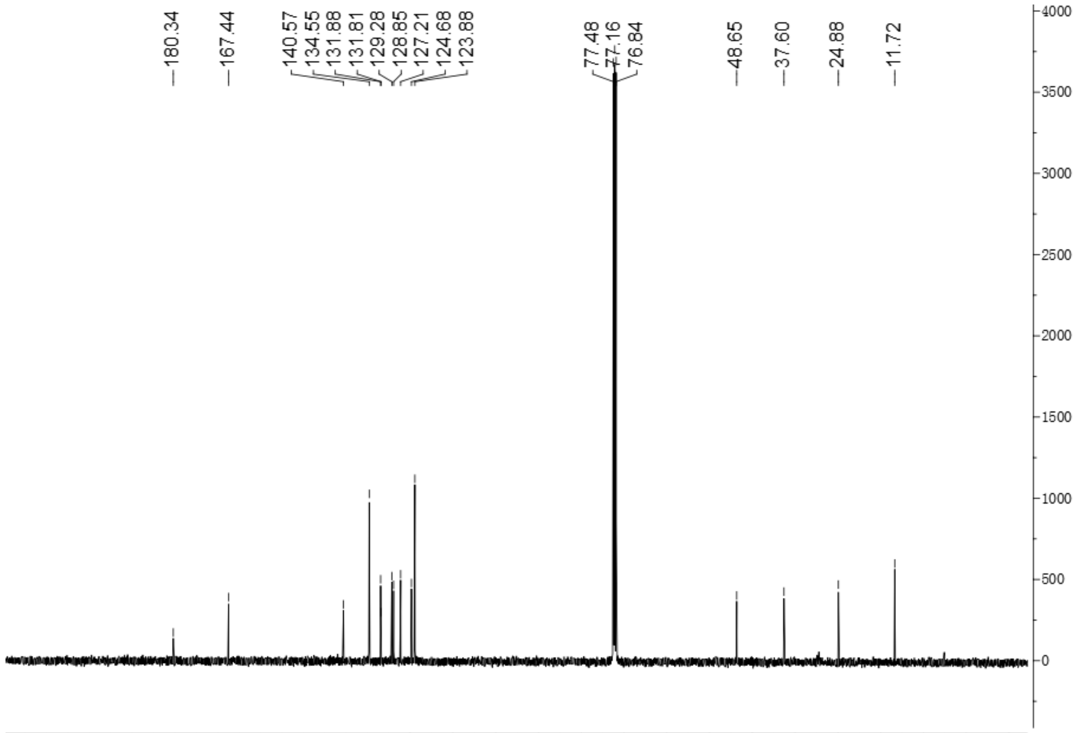

Supplementary Figure 42. ^{1}H and ^{13}C NMR spectra for 12

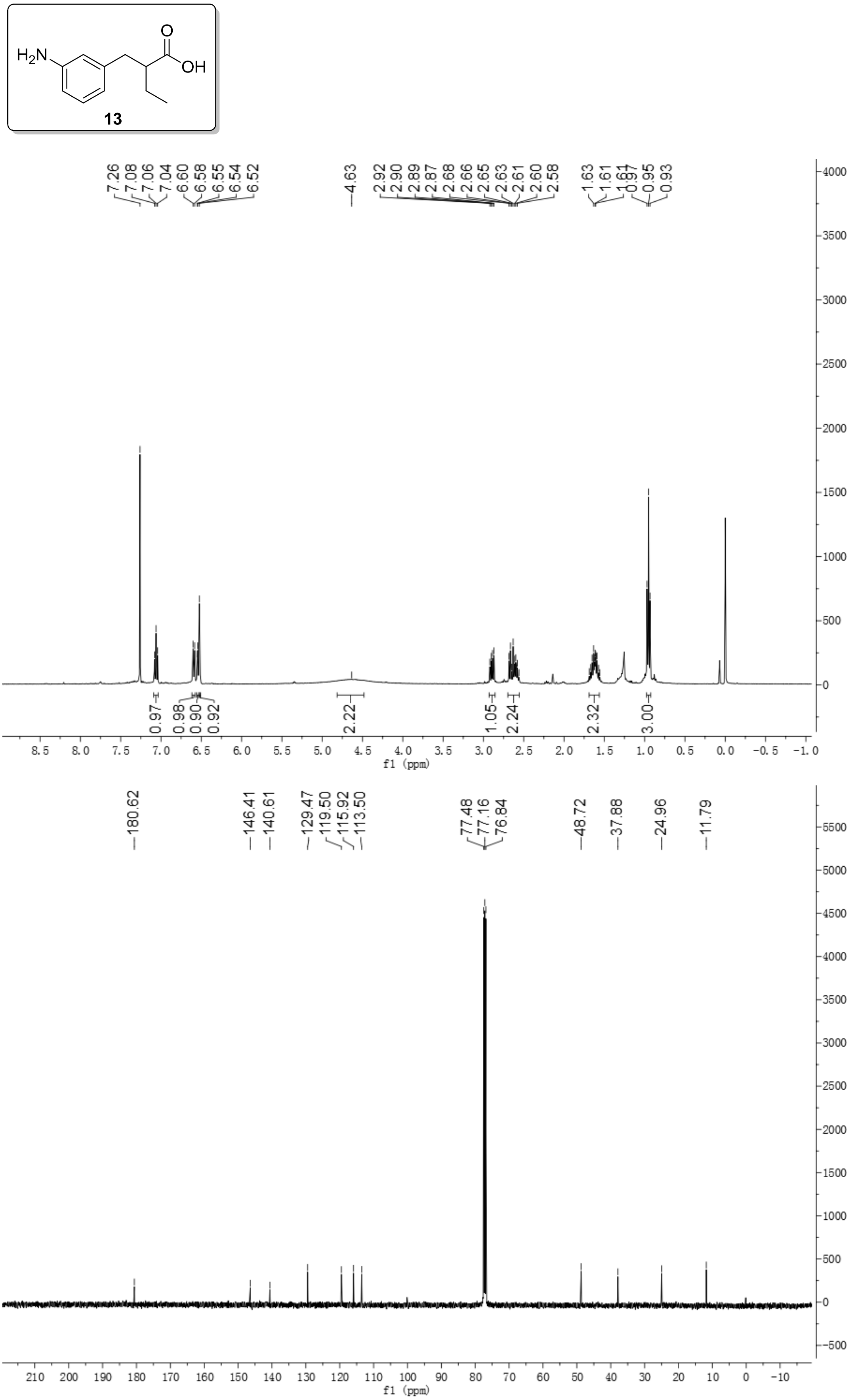

Supplementary Figure 43. ^{1}H and ^{13}C NMR spectra for 13

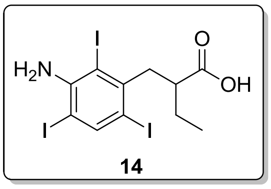

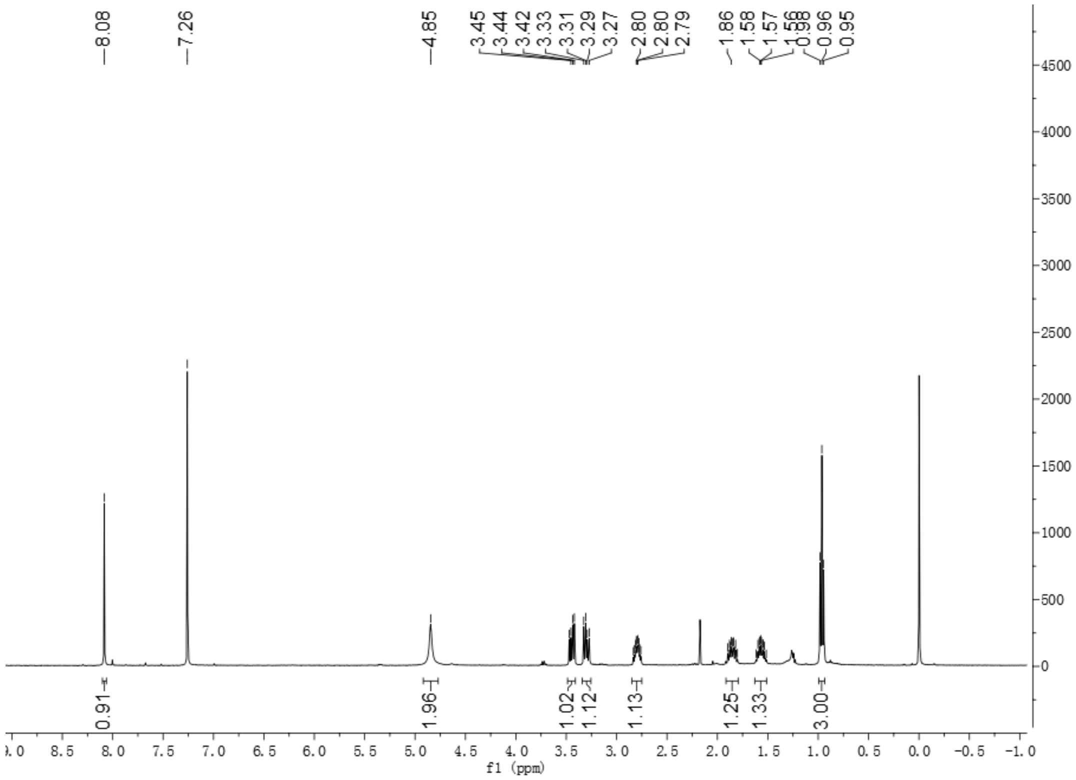

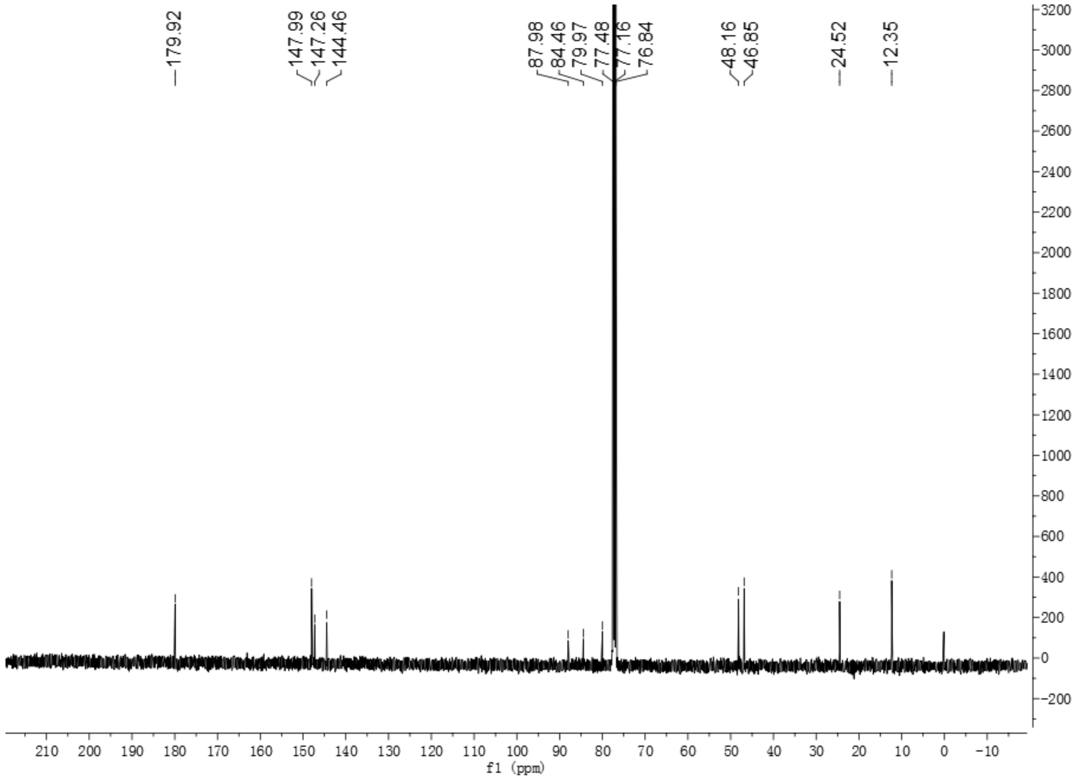

Supplementary Figure 44. ^{1}H and ^{13}C NMR spectra for 14

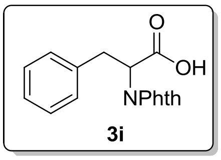

3i

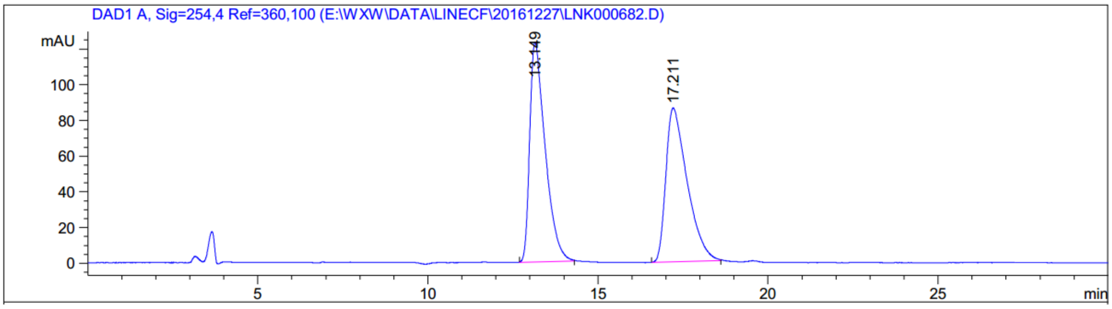

===

Area Percent Report

===

Sorted By : Signal
Multiplier: : 1.0000
Dilution: : 1.0000
Sample Amount: : 1.00000 [ng/ul] (not used in calc.)
Use Multiplier & Dilution Factor with ISTDs

Signal 1: DAD1 A, Sig=254,4 Ref=360,100

Peak #	RetTime [min]	Type	Width [min]	Area [mAU*s]	Height [mAU]	Area %
1	13.149	BB	0.4702	3881.52856	122.99026	50.8678
2	17.211	BB	0.6417	3749.08960	86.14812	49.1322

Totals : 7630.61816 209.13838

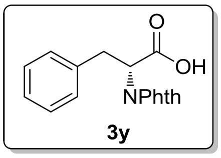

3y

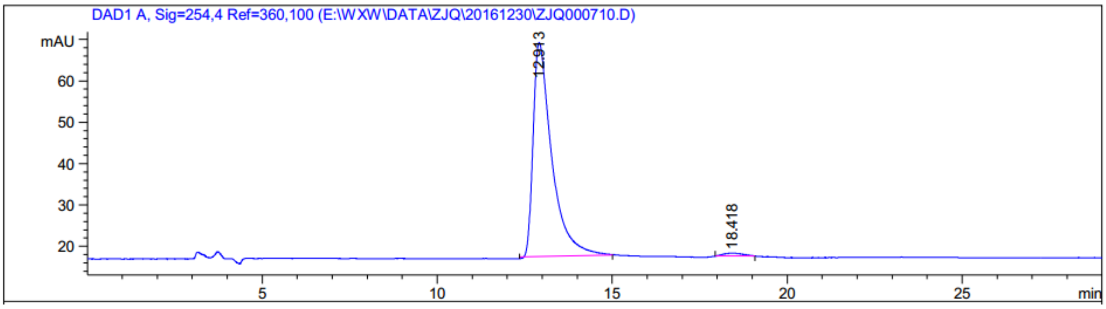

===
Area Percent Report
===

Sorted By : Signal
Multiplier: : 1.0000
Dilution: : 1.0000
Sample Amount: : 1.00000 [ng/ul] (not used in calc.)
Use Multiplier & Dilution Factor with ISTDs

Signal 1: DAD1 A, Sig=254,4 Ref=360,100

Peak #	RetTime [min]	Type	Width [min]	Area [mAU*s]	Height [mAU]	Area %
1	12.913	MM R	0.6178	1915.91809	51.68581	98.7332
2	18.418	MM R	0.6082	24.58137	6.73573e-1	1.2668
Totals :				1940.49946	52.35939	

Supplementary Figure 45. HPLC spectra of 3i and 3y

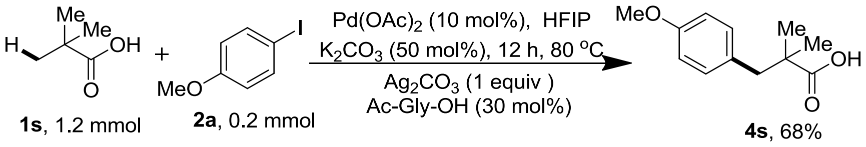

Supplementary Figure 46. Arylation of 1s

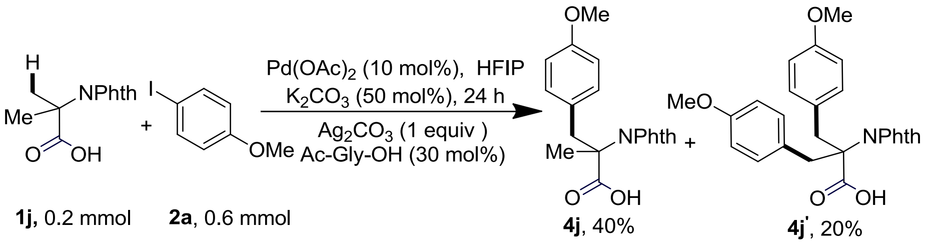

Supplementary Figure 47. Arylation of 1j

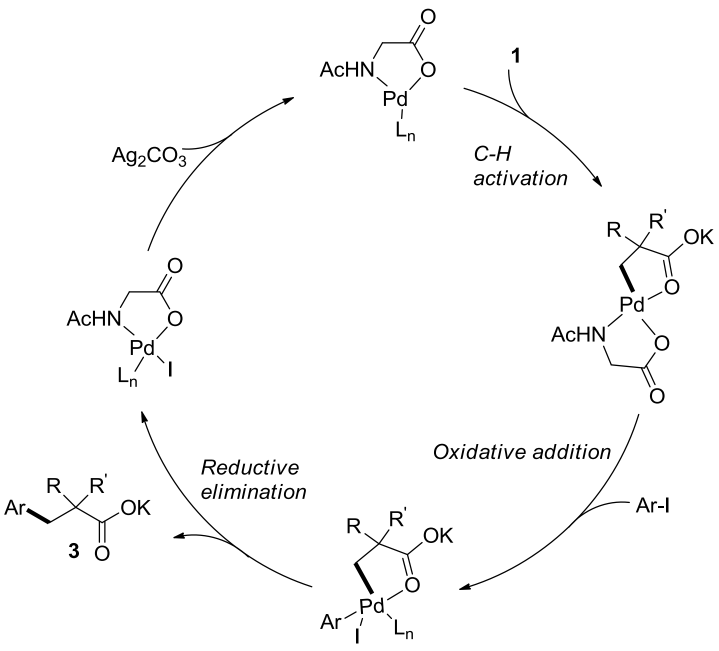

Supplementary Figure 48. Proposed catalytic cycle

Supplementary Table 1. Substrate 1a, 1i, 1j, 1k

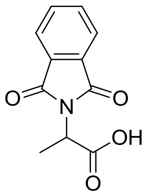	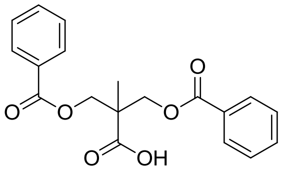	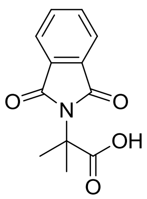	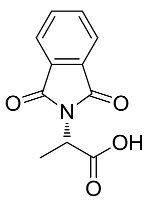
**1a**	**1i**	**1j**	**1k**

Supplementary Table 2. Substrate scope of aryl iodides

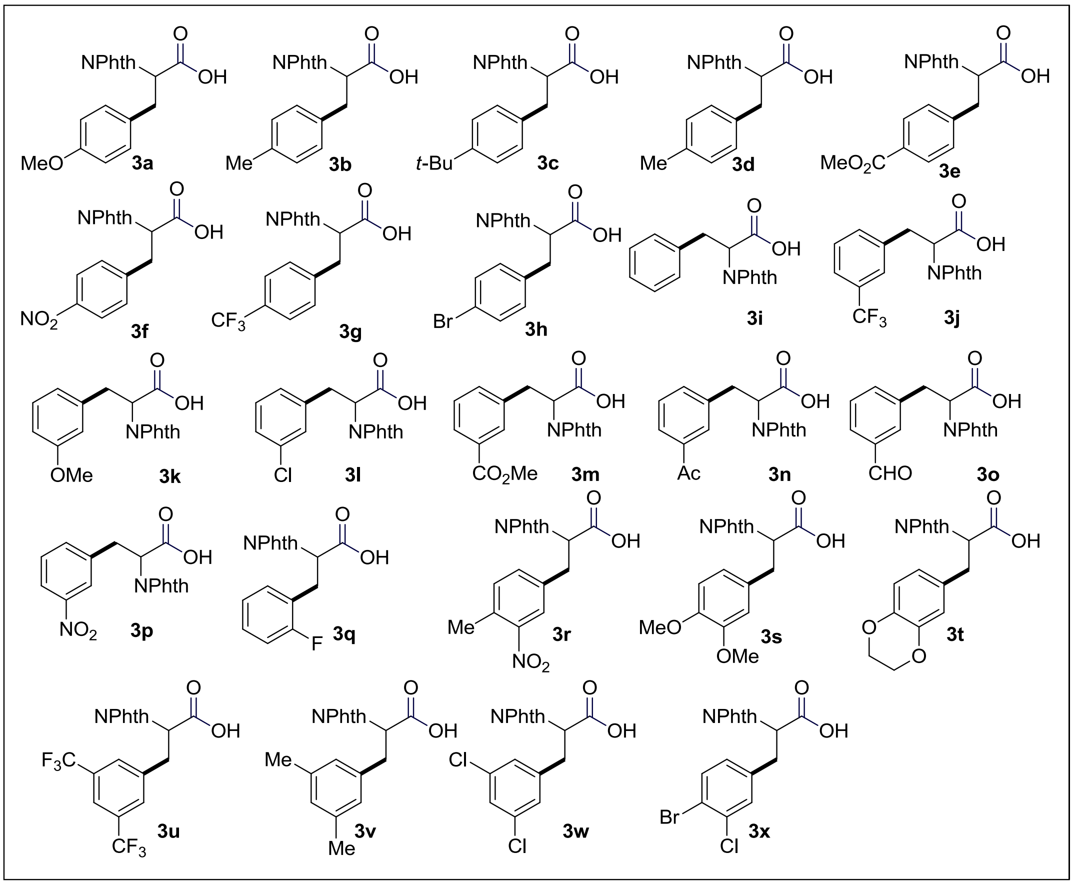

Supplementary Table 3. Substrate scope of carboxylic acids and amino acids

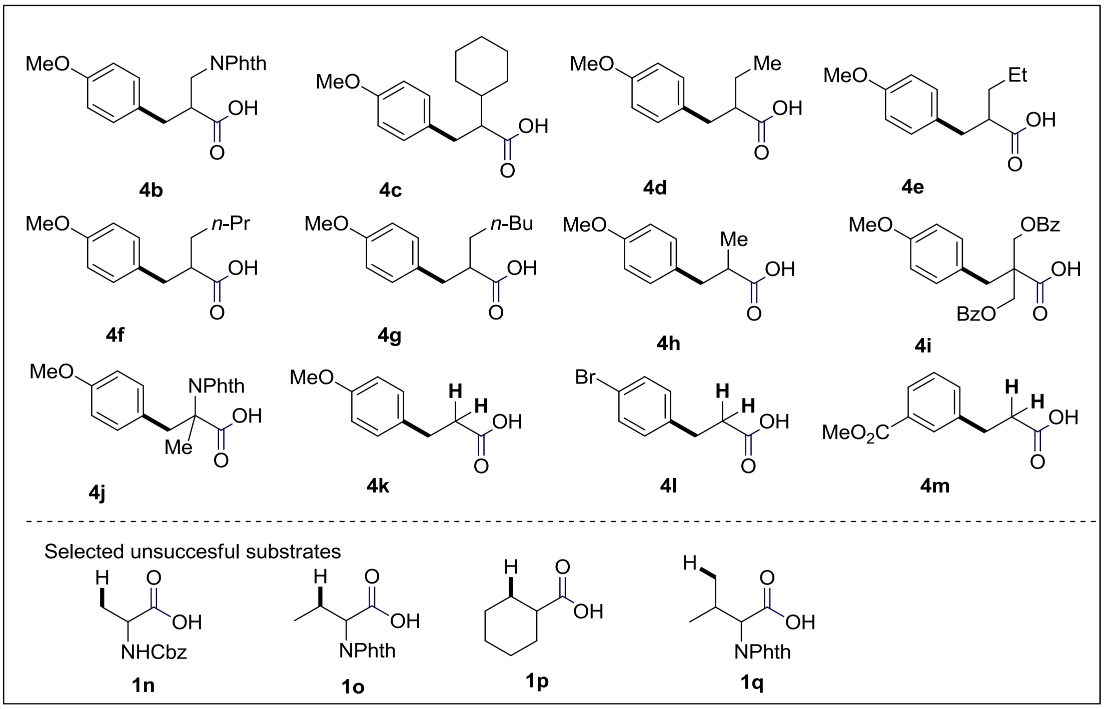

Supplementary Table 4. Screening of solvent[a]

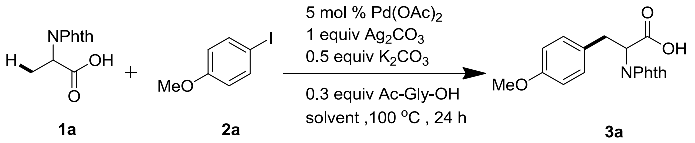

Entry	Solvent	Yield (%)[c]
1	DCE	3
2	toluene	4
3	m-xylene	35
4	mesitylene	5
5	PhCl	3
6	HFIP	56
7	CF_3CH_2OH	20
8	t-AmylOH	18
9	1,4-dioxane	35
[b]10	HFIP	86

Notes: [a]**1a** (0.1 mmol), **2a** (0.15 mmol), $Pd(OAc)_2$ (5 mol%), Ag_2CO_3(0.1 mmol), K_2CO_3(0.05 mmol), Ac-Gly-OH (0.03 mmol), solvent (0.5 mL), 100 °C, 24 h. [b]HFIP (1mL).[c]Yields were based on LC-MS analysis using acetyl benzene as an internal standard.

Supplementary Table 5. Screening of additive 1[a]

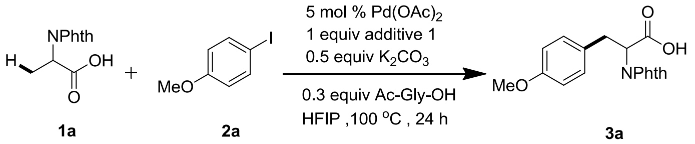

Entry	Additive 1	Yield (%)[b]
1	AgOAc (2 equiv)	24
2	Ag_2O	trace
3	AgF (2 equiv)	10
4	$AgNO_3$ (2 equiv)	28
5	AgOTs (2 equiv)	0
6	AgOTf (2 equiv)	0
7	Ag_2CO_3	86
8	BQ	0
9	$Cu(OAc)_2$	5
10	$K_2S_2O_8$	0
11	$Cu(OAc)_2/O_2$	trace

Notes: [a]**1a** (0.1 mmol), **2a** (0.15 mmol), $Pd(OAc)_2$ (5 mol%), additive 1 (0.1 mmol), K_2CO_3(0.05 mmol), Ac-Gly-OH (0.03 mmol), HFIP (1 mL), 100 °C, 24 h. [b]Yields were based on LC-MS analysis using acetyl benzene as an internal standard.

Supplementary Table 6. Screening of base[a]

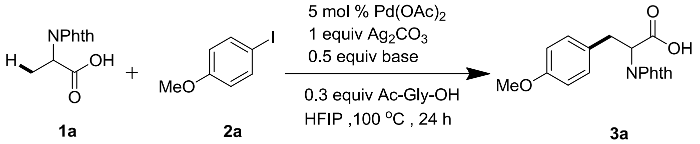

Entry	Base	Yield (%)[b]
1	KOAc	36
2	Cs_2CO_3	42
3	Na_2CO_3	16
4	Li_2CO_3	7
5	LiOAc	14
6	KF	46
7	$KHCO_3$	64
8	K_2CO_3	86

Notes: [a]**1a** (0.1 mmol), **2a** (0.15 mmol), $Pd(OAc)_2$ (5 mol%), Ag_2CO_3 (0.1 mmol), base (0.05 mmol), Ac-Gly-OH (0.03 mmol), HFIP (1 mL), 100 °C, 24 h.
[b]Yields were based on LC-MS analysis using acetyl benzene as an internal standard.

Supplementary Table 7. Screening of ligand[a]

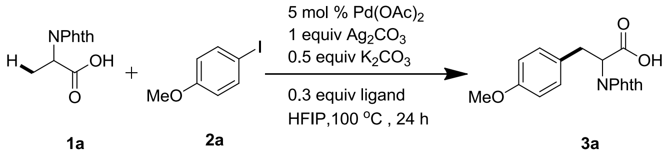

Entry	Ligand	yield(%)[b]	Entry	Ligand	yield(%)[b]
1	Ac-Gly-OH	86			
2	Ac-Leu-OH	65			
3	Ac-Val-OH	70	16	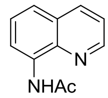	0
4	Ac-isoLeu-OH	54			
5	Boc-Gly-OH	5			
6	Boc-L-Valine	trace			
7	Cbz-Gly-OH	4	17	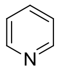	0
8	Fmoc-Val-OH	0			
9	dppf	trace			
10	PPh_3	10	18	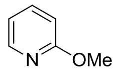	0
11	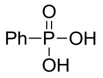	0	19	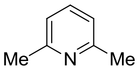	0
12	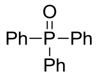	0	20	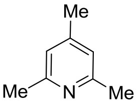	0
13	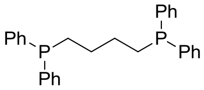	0	21	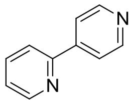	0
14	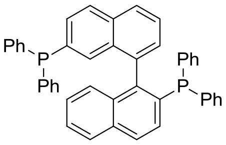	0	22	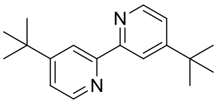	0
15	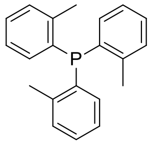	trace	23	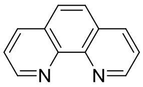	trace

Notes: [a]**1a** (0.1 mmol), **2a** (0.15 mmol), $Pd(OAc)_2$ (5 mol%), Ag_2CO_3 (0.1 mmol), K_2CO_3 (0.05 mmol), ligand (0.03 mmol), HFIP (1 mL), 100 °C, 24 h. [b]Yields were based on LC-MS analysis using acetyl benzene as an internal standard.

Supplementary Table 8. Screening of additive 2[a]

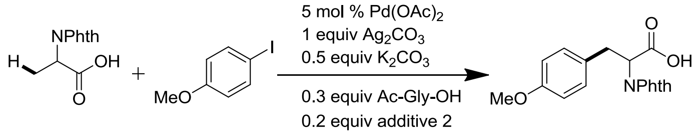

1a **2a** **3a**

**Entry**	**Additive 2**	**Yield (%)[b]**
1	none	86
2	1-$AdCO_2H$	26
3	HOAc	trace
4	(n-BuO)$_2$PO$_2$H	40
5	(BnO)$_2$PO$_2$H	14

Notes: [a]**1a** (0.1 mmol), **2a** (0.15 mmol), $Pd(OAc)_2$ (5 mol%), Ag_2CO_3 (0.1 mmol), K_2CO_3 (0.05 mmol), Ac-Gly-OH (0.03 mmol), additive 2 (0.02 mmol) HFIP (1 mL),100 °C, 24 h. [b]Yields were based on LC-MS analysis using acetyl benzene as an internal standard.

Supplementary Table 9. Condition optimization of 1d[a]

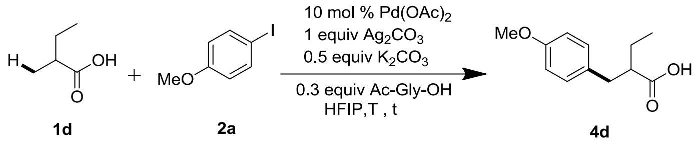

Entry	T (°C)	t (h)	Yield (%)[b]
1	80	12	68
2	100	12	46
3	80	24	50
[c]4	80	12	18
[d]5	80	12	28

Notes: [a]**1d** (0.2 mmol), **2a** (0.1 mmol), $Pd(OAc)_2$ (10 mol%), Ag_2CO_3 (0.1 mmol), K_2CO_3 (0.05 mmol), Ac-Gly-OH (0.03 mmol), HFIP (1 mL), 80 °C, 12 h.
[b]Yields were based on GC-MS analysis using n-tridecane as an internal standard.
[c]K_2CO_3 (0.1 mmol) [d]**1d** (0.1 mmol), **2a** (0.15 mmol).

Supplementary Methods

Instrumentation and Chemical

Unless otherwise noted, all reagents were purchased from Acros, Alfa, Adamas and used without further purification. Column chromatography purifications were performed using 300–400 mesh silica gel. NMR spectra were recorded on Varian Inova-400 MHz, Inova-300 MHz, Bruker DRX-400 or Bruker DRX-500 instruments and calibrated using residual solvent peaks as internal reference. Multiplicities are recorded as: s = singlet, d = doublet, t = triplet, dd = doublet of doublets, m = multiplet. HRMS analysis were carried out using TOF-MS instrument with ESI source.

1. Experimental Procedures:

1.1. Preparation of Substrates

1.1.1. General Procedures for the Preparation of 1a, 1i, 1j, 1k

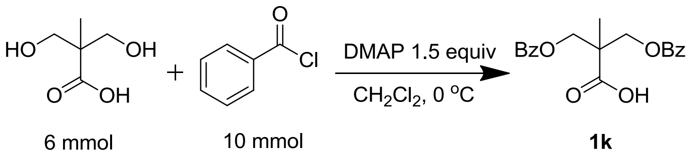

A solution of benzoyl chloride (1.2 ml, 10 mmol) in CH_2Cl_2 (10 mL) was added dropwise to a solution of 2,2-Bis(hydroxymethyl) propionic acid (0.8g, 6 mmol, 0.6 equiv) and DMAP (1.8 g, 1.5 equiv) in CH_2Cl_2 (10 mL) at 0 ℃. The solution was warmed to room temperature and stirred over night. Then, the reaction was quenched by water (15 mL). The organic layer was separated and the aqueous layer was extracted with CH_2Cl_2 (5 mL × 3). The combined organic phase was washed with brine (15 mL), and then dried over anhydrous Na_2SO_4. Evaporation and column chromatography on silica gel afforded corresponding amide substrates as white solid with >80% yield. **1a**, **1j** were prepared according to literature report[1]. **1k** was purchased from Fluorochem Ltd.

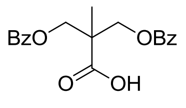

1i

White solid. ^{1}H NMR (400 MHz, $CDCl_3$) δ 8.01 – 7.98 (m, 4H), 7.57 – 7.53 (m, 2H), 7.40 (dd, *J* = 10.7, 4.8 Hz, 4H), 4.60 (s, 4H), 1.47 (s, 3H). ^{13}C NMR (101 MHz, $CDCl_3$) δ 177.84, 166.15, 133.42, 129.72 (d, *J* = 11.4 Hz), 128.62, 66.05, 46.70, 18.08. HRMS Calcd for $C_{19}H_{18}O_6$ [M+H$^+$]: 343.1182; Found: 343.1179.

1.2. General Procedure for the reaction of 1a with different aryl iodides.

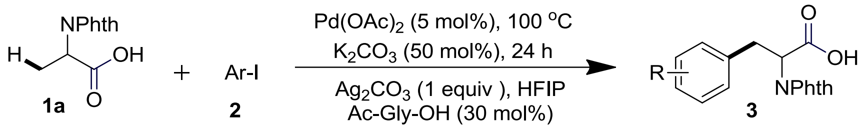

A mixture of **1a** (0.2 mmol, 43.8 mg), **2** (0.3 mmol, 1.5 equiv), $Pd(OAc)_2$ (2.2 mg, 5 mol%), Ag_2CO_3 (55.2 mg, 0.2 mmol, 1 equiv), K_2CO_3 (13.8 mg, 0.1 mmol, 0.5 equiv), Ac-gly-OH (7 mg, 0.06 mmol, 0.3 equiv) and 2 mL HFIP in a 15 mL sealed glass vial was heated at 100 ^{o}C with vigorous stirring for 24 hours. The reaction mixture was cooled to room temperature, and diluted with ethyl acetate and filtered through celite. The filtrate was concentrated in vacuo and purified by column chromatography on silica gel (Ethyl acetate/Petroleum ether/HCOOH = 1:15:0.5% to 1:6:0.5%) to give product.

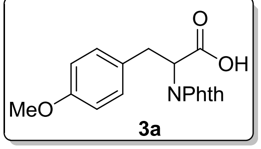

3a

White solid; Yield (83%, 54.0 mg); R_f = 0.53 (petroleum ester/ethyl acetate/HCOOH, 2/1/0.02). ^{1}H NMR (400 MHz, $CDCl_3$) δ 7.78 (dd, *J* = 5.5, 3.0 Hz, 2H), 7.68 (dd, *J* = 5.5, 3.1 Hz, 2H), 7.07 (d, *J* = 8.6 Hz, 2H), 6.71 (d, *J* = 8.7 Hz, 2H), 5.17 (dd, *J* = 9.0, 7.7 Hz, 1H), 3.70 (s, 3H), 3.53 (d, *J* = 8.5 Hz, 2H). ^{13}C NMR (101 MHz, $CDCl_3$) δ 174.22, 167.55, 158.56, 134.29, 131.65, 129.97, 128.48, 123.70, 114.15, 55.27, 53.32, 33.67. HRMS data for the desired product were in agreement with the previously reported literature data[2].

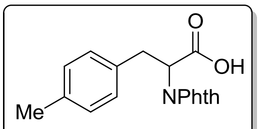

3b

White solid; Yield (86%, 53.1 mg); R_f = 0.52 (petroleum ester/ethyl acetate/HCOOH, 2/1/0.02). ^{1}H NMR (400 MHz, $CDCl_3$) δ 7.78 (d, *J* = 3.0 Hz, 2H), 7.69 (d, *J* = 2.8 Hz, 2H), 7.04 (d, *J* = 7.6 Hz, 2H), 6.98 (d, *J* = 7.6 Hz, 2H), 5.19 (t, *J* = 8.2 Hz, 1H), 3.55 (d, *J* = 8.2 Hz, 2H), 2.22 (s, 3H). ^{13}C NMR (101 MHz, $CDCl_3$) δ 173.94, 167.56, 136.58, 134.26, 133.42, 131.69, 129.44, 128.79, 123.68,

53.27, 34.11, 21.15. HRMS data for the desired product were in agreement with the previously reported literature data[3].

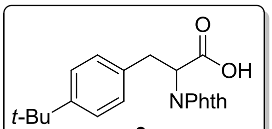

3c

White solid; Yield (79%, 55.5 mg); $R_f = 0.50$ (petroleum ester/ethyl acetate/HCOOH, 2/1/0.02). ^{1}H NMR (400 MHz, $CDCl_3$) δ 7.79 (dd, *J* = 5.4, 3.1 Hz, 2H), 7.68 (dd, *J* = 5.4, 3.1 Hz, 2H), 7.20 (d, *J* = 8.2 Hz, 2H), 7.09 (d, *J* = 8.2 Hz, 2H), 5.22 (t, *J* = 8.2 Hz, 1H), 3.56 (d, *J* = 8.2 Hz, 2H), 1.21 (s, 9H). ^{13}C NMR (101 MHz, $CDCl_3$) δ 174.13, 167.62, 149.83, 134.25, 133.47, 131.75, 128.58, 125.61, 123.64, 53.18, 34.49, 34.00, 31.38. HRMS Calcd for $C_{21}H_{21}NO_4$ [M+H$^+$]: 352.1549; Found: 352.1560.

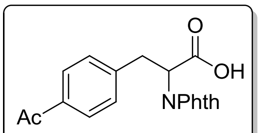

3d

White solid; Yield (71%, 47.9 mg); $R_f = 0.48$ (petroleum ester/ethyl acetate/HCOOCH, 2/1/0.02). ^{1}H NMR (400 MHz, $CDCl_3$) δ 7.76 (d, *J* = 8.3 Hz, 2H), 7.75 – 7.73 (m, 2H), 7.67 (dd, *J* = 5.4, 3.1 Hz, 2H), 7.23 (d, *J* = 8.1 Hz, 2H), 5.17 (t, *J* = 8.3 Hz, 1H), 3.58 (d, *J* = 8.2 Hz, 2H), 2.51 (s, 3H). ^{13}C NMR (101 MHz, DMSO) δ 197.46, 169.87, 167.16, 143.20, 135.25, 135.01, 130.71, 129.08, 128.30, 123.47, 52.72, 34.01, 26.62. HRMS Calcd for $C_{19}H_{15}NO_5$ [M+H$^+$]: 338.1028; Found: 338.1041.

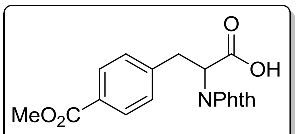

3e

White solid; Yield (63%, 44.5 mg); $R_f = 0.53$ (petroleum ester/ethyl acetate/HCOOH, 2/1/0.02). ^{1}H NMR (400 MHz, $CDCl_3$) δ 7.86 (d, *J* = 8.3 Hz, 2H), 7.78 (dd, *J* = 5.5, 3.0 Hz, 2H), 7.69 (dd, *J* = 5.5, 3.0 Hz, 2H), 7.25 (d, *J* = 8.3 Hz, 2H), 5.23 (dd, *J* = 9.7, 6.9 Hz, 1H), 3.85 (s, 3H), 3.66 – 3.63 (m, 2H). ^{13}C NMR (101 MHz, $CDCl_3$) δ 173.21, 167.48, 167.02, 142.02, 134.45, 131.50, 130.08, 129.06, 123.79, 52.70, 52.21, 34.58. HRMS Calcd for $C_{19}H_{15}NO_6$ [M+H$^+$]: 354.0978; Found: 354.0995.

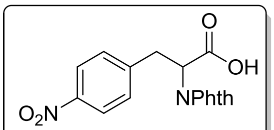

3f

White solid; Yield (80%, 54.4 mg); $R_f = 0.49$ (petroleum ester/ethyl acetate/HCOOH, 2/1/0.02). ^1H NMR (400 MHz, $CDCl_3$) δ 8.08 – 8.06 (m, 2H), 7.80 (dd, J = 5.5, 3.0 Hz, 2H), 7.72 (dd, J = 5.5, 3.1 Hz, 2H), 7.36 (d, J = 8.7 Hz, 2H), 5.25 (dd, J = 9.7, 6.9 Hz, 1H), 3.69 (s, 2H). ^{13}C NMR (101 MHz, $CDCl_3$) δ 173.06, 167.45, 147.24, 144.26, 134.67, 131.36, 129.93, 124.04, 123.93, 52.38, 34.52. HRMS data for the desired product were in agreement with the previously reported literature data[4].

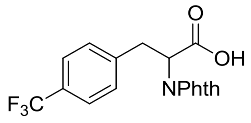

3g

White solid; Yield (77%, 55.9 mg); $R_f = 0.46$ (petroleum ester/ethyl acetate/HCOOH, 2/1/0.02). ^1H NMR (400 MHz, $CDCl_3$) δ 7.80 (dd, J = 5.5, 3.1 Hz, 2H), 7.71 (dd, J = 5.5, 3.0 Hz, 2H), 7.46 (d, J = 8.1 Hz, 2H), 7.30 (d, J = 8.1 Hz, 2H), 5.25 (s, 1H), 3.67 – 3.64 (m, 2H). ^{13}C NMR (101 MHz, $CDCl_3$) δ 173.89, 167.51, 140.68, 134.54, 131.49, 129.33, 123.85, 127.58 (q, $J_{C\text{-}F}$ = 271.0 Hz), 125.79 (q, $J_{C\text{-}F}$ =4.0 Hz), 52.64, 34.38. ^{19}F NMR (376 MHz, $CDCl_3$) δ -62.57. HRMS Calcd for $C_{18}H_{12}F_3NO_4$ [M+H$^+$]: 364.0797; Found:364.0799.

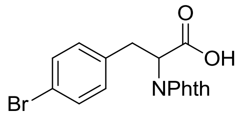

3h

White solid; Yield (68%, 50.9 mg); $R_f = 0.50$ (petroleum ester/ethyl acetate/HCOOH, 2/1/0.02). ^1H NMR (400 MHz, $CDCl_3$) δ 7.79 (dd, J = 5.5, 3.0 Hz, 2H), 7.70 (dd, J = 5.5, 3.1 Hz, 2H), 7.31 (d, J = 8.4 Hz, 2H), 7.04 (d, J = 8.4 Hz, 2H), 5.18 (dd, J = 9.9, 6.7 Hz, 1H), 3.56 – 3.53 (m, 2H). ^{13}C NMR (101 MHz, $CDCl_3$) δ 173.73, 167.51, 135.54, 134.47, 131.90, 131.53, 130.69, 123.82, 121.10, 52.78, 34.00. HRMS data for the desired product were in agreement with the previously reported literature data[4].

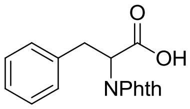

3i

White solid; Yield (69%, 40.7 mg); $R_f = 0.45$ (petroleum ester/ethyl acetate/HCOOH, 2/1/0.02). ^1H NMR (400 MHz, $CDCl_3$) δ 7.78 (dd, J = 5.5, 3.0 Hz, 2H), 7.68 (dd, J = 5.5, 3.1 Hz, 2H), 7.21 – 7.13 (m, 5H), 5.22 (dd, J = 9.0, 7.6 Hz, 1H), 3.59 (d, J = 8.7 Hz, 2H). ^{13}C NMR (101 MHz, $CDCl_3$) δ 174.40, 167.52, 136.54, 134.30, 131.62, 128.95, 128.74, 127.08, 123.69, 53.17, 34.52. HRMS data for the desired product were in agreement with the previously reported literature data[5].

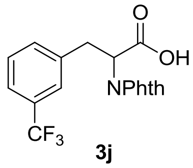

3j

White solid; Yield (77%, 55.9 mg); R_f = 0.46 (petroleum ester/ethyl acetate/HCOOH, 2/1/0.02). ^{1}H NMR (400 MHz, $CDCl_3$) δ 7.84 (dd, *J* = 5.5, 3.1 Hz, 2H), 7.75 (dd, *J* = 5.5, 3.1 Hz, 2H), 7.46 (d, *J* = 9.1 Hz, 2H), 7.44 (d, *J* = 3.0 Hz, 1H), 7.41 – 7.37 (m, 1H), 5.26 (t, *J* = 8.2 Hz, 1H), 3.69 (d, *J* = 8.3 Hz, 2H).^{13}C NMR (101 MHz, $CDCl_3$) δ 173.79, 167.48, 137.61, 134.50, 132.35, 131.51 (q, J_{C-F} = 33.0 Hz), 131.47, 129.28, 125.91 (q, J_{C-F} = 3.0 Hz), 123.97 (q, J_{C-F} = 271.0 Hz), 124.1 (q, J_{C-F} = 4.0 Hz), 123.79, 52.77, 34.39. ^{19}F NMR (376 MHz, $CDCl_3$) δ -62.85. HRMS Calcd for $C_{18}H_{12}F_3NO_4$ [M+H$^+$]: 364.0797; Found: 364.0806.

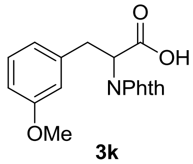

3k

White solid; Yield (62%, 40.3 mg); R_f = 0.44 (petroleum ester/ethyl acetate/HCOOH, 2/1/0.02). ^{1}H NMR (400 MHz, $CDCl_3$) δ 7.78 (dd, *J* = 5.5, 3.0 Hz, 2H), 7.68 (dd, *J* = 5.4, 3.1 Hz, 2H), 7.10 (t, *J* = 7.8 Hz, 1H), 6.75 (d, *J* = 7.6 Hz, 1H), 6.68 (dd, *J* = 11.1, 2.2 Hz, 2H), 5.22 (t, *J* = 8.3 Hz, 1H), 3.67 (s, 3H), 3.56 (d, *J* = 8.3 Hz, 2H). ^{13}C NMR (101 MHz, $CDCl_3$) δ 173.10, 167.55, 159.78, 138.13, 134.32, 131.69, 129.75, 123.69, 121.27, 114.20, 112.96, 55.23, 53.01, 34.60. HRMS Calcd for $C_{18}H_{15}NO_5$ [M+H$^+$]:326.1028; Found: 326.1034.

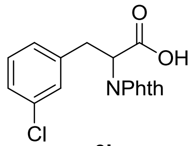

3l

White solid; Yield (40%, 26.0 mg); R_f = 0.43 (petroleum ester/ethyl acetate/HCOOH, 2/1/0.02). ^{1}H NMR (400 MHz, $CDCl_3$) δ 7.80 (dd, *J* = 5.5, 3.1 Hz, 2H), 7.70 (dd, *J* = 5.5, 3.1 Hz, 2H), 7.16 (s, 1H), 7.13 (dd, *J* = 4.8, 1.0 Hz, 2H), 7.09 – 7.04 (m, 1H), 5.19 (dd, *J* = 9.0, 7.5 Hz, 1H), 3.56 (d, *J* = 8.6 Hz, 2H). ^{13}C NMR (101 MHz, $CDCl_3$) δ 173.81, 167.54, 138.64, 134.49, 134.45, 131.55, 130.01, 129.21, 127.39, 127.07, 123.80, 52.86, 34.26. HRMS Calcd for $C_{17}H_{12}ClNO_4$ [M+H$^+$]: 330.0533; Found: 330.0546.

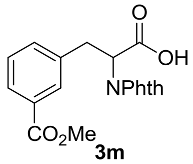

3m

White solid; Yield (87%, 61.4 mg); R_f = 0.53 (petroleum ester/ethyl acetate/HCOOH, 2/1/0.02). ^{1}H NMR (400 MHz, $CDCl_3$) δ 7.85 (s, 1H), 7.82 (d, *J* = 7.8 Hz, 1H), 7.78 (dd, *J* = 5.4, 3.1 Hz, 2H),

7.69 (dd, J = 5.5, 3.0 Hz, 2H), 7.37 (d, J = 7.7 Hz, 1H), 7.27 (t, J = 7.6 Hz, 2H), 5.22 (dd, J = 8.9, 7.5 Hz, 1H), 3.83 (s, 3H), 3.64 (d, J = 8.4 Hz, 2H). ^{13}C NMR (101 MHz, $CDCl_3$) δ 173.74, 167.48, 166.95, 137.03, 134.39, 133.50, 131.57, 130.63, 130.17, 128.87, 128.47, 123.77, 52.97, 52.26, 34.37. HRMS Calcd for $C_{19}H_{15}NO_6$ [M+H$^+$]: 354.0978; Found: 354.0990.

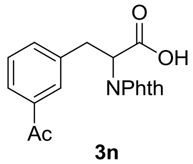

3n

White solid; Yield (68%, 45.8 mg); R_f = 0.54 (petroleum ester/ethyl acetate/HCOOH, 2/1/0.02). ^{1}H NMR (400 MHz, $CDCl_3$) δ 7.79 (dd, J = 5.5, 3.0 Hz, 2H), 7.77-7.74 (m, 2H), 7.70 (dd, J = 5.5, 3.1 Hz, 2H), 7.39 (d, J = 7.7 Hz, 1H), 7.31 (t, J = 7.9 Hz, 1H), 5.24 (dd, J = 8.7, 7.7 Hz, 1H), 3.66 (d, J = 8.4 Hz, 2H), 2.49 (s, 3H). ^{13}C NMR (101 MHz, $CDCl_3$) δ 198.12, 172.98, 167.48, 137.52, 137.21, 134.48, 133.73, 131.54, 129.12, 128.97, 127.15, 123.78, 52.82, 34.46, 26.72. HRMS Calcd for $C_{19}H_{15}NO_5$ [M+H$^+$]: 338.1028; Found: 338.1033.

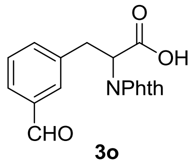

3o

White solid; Yield (62%, 40.1 mg); R_f = 0.50 (petroleum ester/ethyl acetate/HCOOH, 2/1/0.02). ^{1}H NMR (400 MHz, $CDCl_3$) δ 9.89 (s, 1H), 7.78 (dd, J = 5.5, 3.1 Hz, 2H), 7.70 – 7.67 (m, 4H), 7.46 (d, J = 7.7 Hz, 1H), 7.38 (t, J = 7.8 Hz, 1H), 5.24 (dd, J = 9.2, 7.2 Hz, 1H), 3.68 – 3.66 (m, 2H). ^{13}C NMR (101 MHz, $CDCl_3$) δ 192.34, 173.78, 167.49, 137.86, 136.79, 135.09, 134.51, 131.46, 130.51, 129.52, 128.47, 123.82, 52.84, 34.34. HRMS Calcd for $C_{18}H_{13}NO_5$ [M+H$^+$]: 324.0872; Found: 324.0880.

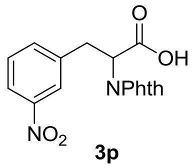

3p

White solid; Yield (68%, 46.2 mg); R_f = 0.49 (petroleum ester/ethyl acetate/HCOOH, 2/1/0.02). ^{1}H NMR (400 MHz, $CDCl_3$) δ 8.05 (s, 1H), 8.03 (d, J = 8.9 Hz, 1H), 7.80 (dd, J = 5.4, 3.1 Hz, 2H), 7.71 (dd, J = 5.5, 3.0 Hz, 2H), 7.53 (d, J = 7.6 Hz, 1H), 7.40 (t, J = 7.9 Hz, 1H), 5.21 (dd, J = 9.7, 6.5 Hz, 1H), 3.71 – 3.65 (m, 2H). ^{13}C NMR (101 MHz, DMSO) δ 169.73, 167.14, 147.63, 139.87, 135.79, 135.02, 130.66, 129.80, 123.57, 123.47, 121.67, 52.55, 33.71. HRMS Calcd for $C_{17}H_{12}N_2O_5$ [M+H$^+$]: 341.0774; Found:341.0770.

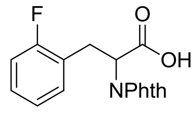

3q

White solid; Yield (72%, 45.1 mg); R_f = 0.47 (petroleum ester/ethyl acetate/HCOOH, 2/1/0.02). ^{1}H NMR (400 MHz, $CDCl_3$) δ 7.79 (dd, *J* = 5.5, 3.1 Hz, 2H), 7.69 (dd, *J* = 5.5, 3.1 Hz, 2H), 7.17 – 7.08 (m, 2H), 6.98– 6.91 (m, 2H), 5.26 (dd, *J* = 11.3, 4.8 Hz, 1H), 3.68–3,54 (m, 2H). ^{13}C NMR (101 MHz, $CDCl_3$) δ 173.66, 167.39, 161.54 (d, $J_{C\text{-}F}$ = 244.0 Hz), 134.33, 131.64, 131.43 (d, $J_{C\text{-}F}$ = 4.0 Hz), 129.15 (d, $J_{C\text{-}F}$ = 8.0 Hz), 124.31 (d, $J_{C\text{-}F}$ = 4.0 Hz), 123.72, 123.70 (d, $J_{C\text{-}F}$ = 15.0 Hz), 115.56 (d, $J_{C\text{-}F}$ = 22.0 Hz), 51.77, 28.82. ^{19}F NMR (376 MHz, $CDCl_3$) δ -117.69 (s). HRMS Calcd for $C_{17}H_{12}FNO_4$ [M+H$^+$]: 314.0829; Found: 314.0839.

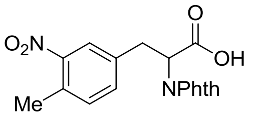

3r

White solid; Yield (80%,56.6 mg); R_f = 0.52 (petroleum ester/ethyl acetate/HCOOH, 2/1/0.02). ^{1}H NMR (400 MHz, $CDCl_3$) δ 7.81 (dd, *J* = 5.5, 3.0 Hz, 2H), 7.78 (d, *J* = 1.5 Hz, 1H), 7.72 (dd, *J* = 5.5, 3.1 Hz, 2H), 7.34 (dd, *J* = 7.9, 1.6 Hz, 1H), 7.20 (d, *J* = 7.9 Hz, 1H), 5.20 (t, *J* = 8.2 Hz, 1H), 3.63 (d, *J* = 8.2 Hz, 2H), 2.49 (s, 3H). ^{13}C NMR (101 MHz, $CDCl_3$) δ 173.00, 167.49, 149.26, 135.99, 134.60, 133.49, 133.31, 132.52, 131.48, 125.17, 123.92, 52.58, 33.85, 20.28. HRMS Calcd for $C_{18}H_{14}N_2O_6$ [M+H$^+$]: 355.0930; Found: 355.0939.

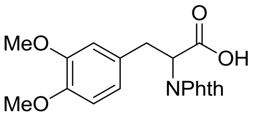

3s

White solid; Yield (75%,53.3 mg); R_f = 0.46 (petroleum ester/ethyl acetate/HCOOH, 2/1/0.02). ^{1}H NMR (400 MHz, $CDCl_3$) δ 7.77 (dd, *J* = 5.5, 3.1 Hz, 2H), 7.67 (dd, *J* = 5.5, 3.1 Hz, 2H), 6.71 (dd, *J* = 8.2, 1.7 Hz, 1H), 6.68 (s, 1H), 6.66 – 6.63 (m, 1H), 5.20 (t, *J* = 8.4 Hz, 1H), 3.76 (s, 3H), 3.69 (s, 3H), 3.53 (d, *J* = 8.4 Hz, 2H). ^{13}C NMR (101 MHz, $CDCl_3$) δ 174.17, 167.57, 148.83, 147.88, 134.33, 131.59, 128.91, 123.64, 121.11, 111.81, 111.32, 55.83, 55.78, 53.14, 34.01. HRMS Calcd for $C_{19}H_{17}NO_6$ [M+H$^+$]: 356.1134; Found: 356.1139.

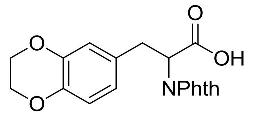

3t

White solid; Yield (69%,48.7 mg); R_f = 0.46 (petroleum ester/ethyl acetate/HCOOH, 2/1/0.02). ^{1}H NMR (400 MHz, $CDCl_3$) δ 7.80 (dd, *J* = 5.5, 3.0 Hz, 2H), 7.69 (dd, *J* = 5.4, 3.1 Hz, 2H), 6.68 (s, 1H), 6.66 (d, *J* = 8.2 Hz, 1H), 6.60 (dd, *J* = 8.3, 2.0 Hz, 1H), 5.13 (d, *J* = 8.2 Hz, 1H), 4.15 (s, 4H), 3.47 (d, *J* = 8.2 Hz, 2H). ^{13}C NMR (101 MHz, $CDCl_3$) δ 173.96, 167.59, 143.55, 142.54, 134.27, 131.72, 129.81, 123.72, 121.84, 117.76, 117.41, 64.34, 53.31, 33.82. HRMS Calcd for $C_{19}H_{15}NO_6$

[M+H$^+$]: 354.0978; Found: 354.0988.

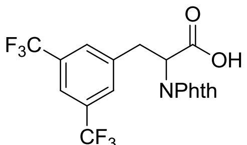

3u

White solid; Yield (78%,67.2 mg); R_f = 0.47 (petroleum ester/ethyl acetate/HCOOH, 2/1/0.02). ^{1}H NMR (400 MHz, $CDCl_3$) δ 7.81 (dd, *J* = 5.5, 3.0 Hz, 2H), 7.72 (dd, *J* = 5.5, 3.1 Hz, 2H), 7.68 (s, 1H), 7.63 (s, 2H), 5.20 (dd, *J* = 9.9, 6.4 Hz, 1H), 3.71 – 3.68 (m, 2H).^{13}C NMR (101 MHz, $CDCl_3$) δ 173.26, 167.42, 139.25, 134.70, 132.37 (q, $J_{C\text{-}F}$ = 33.0 Hz), 131.34, 129.34 (d, $J_{C\text{-}F}$ = 2.0 Hz), 125.89 (q, $J_{C\text{-}F}$ = 271.0 Hz), 123.90, 121.35 (q, $J_{C\text{-}F}$ = 4.0 Hz), 52.34, 34.39. ^{19}F NMR (376 MHz, $CDCl_3$) δ -63.04. HRMS Calcd for $C_{19}H_{11}F_6NO_4$ [M+H$^+$]: 432.0671; Found: 432.0681.

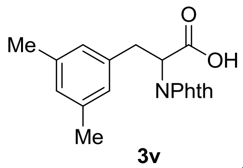

3v

White solid; Yield (90%,58.1 mg); R_f = 0.52 (petroleum ester/ethyl acetate/HCOOH, 2/1/0.02). ^{1}H NMR (400 MHz, $CDCl_3$) δ 7.79 (dd, *J* = 5.4, 3.1 Hz, 2H), 7.68 (dd, *J* = 5.5, 3.0 Hz, 2H), 6.77 (s, 3H), 5.20 (t, *J* = 8.2 Hz, 1H), 3.51 (d, *J* = 8.2 Hz, 2H), 2.15 (s, 6H). ^{13}C NMR (101 MHz, $CDCl_3$) δ 174.81, 167.58, 138.15, 136.39, 134.24, 131.71, 128.67, 126.72, 123.64, 53.27, 34.29, 21.24. HRMS Calcd for $C_{19}H_{17}NO_4$ [M+H$^+$]: 324.1236; Found: 324.1243.

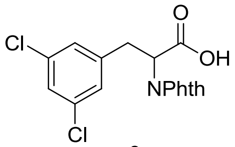

3w

White solid; Yield (62%,45.1 mg); R_f = 0.47 (petroleum ester/ethyl acetate/HCOOH, 2/1/0.02). ^{1}H NMR (400 MHz, $CDCl_3$) δ 7.83 (dd, *J* = 5.5, 3.1 Hz, 2H), 7.73 (dd, *J* = 5.5, 3.1 Hz, 2H), 7.16 (d, *J* = 1.8 Hz, 1H), 7.08 (d, *J* = 1.8 Hz, 2H), 5.17 (dd, *J* = 9.7, 6.6 Hz, 1H), 3.55 – 3.53 (m, 2H). ^{13}C NMR (101 MHz, $CDCl_3$) δ 173.43, 167.50, 139.97, 135.22, 134.59, 131.50, 127.57, 127.54, 123.92, 52.50, 34.12. HRMS Calcd for $C_{17}H_{11}Cl_2NO_4$ [M+H$^+$]: 364.0143; Found: 364.0158.

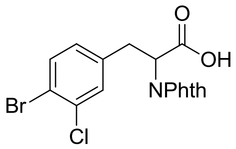

3x

White solid; Yield (62%,58.8 mg); R_f = 0.50 (petroleum ester/ethyl acetate/HCOOH, 2/1/0.02). ^{1}H NMR (400 MHz, $CDCl_3$) δ 7.81 (dd, *J* = 5.2, 3.1 Hz, 2H), 7.72 (dd, *J* = 5.3, 3.0 Hz, 2H), 7.43 (d, *J* = 8.2 Hz, 1H), 7.28 (s, 1H), 6.94 (d, *J* = 6.8 Hz, 1H), 5.18 (t, *J* = 8.2 Hz, 1H), 3.53 (d, *J* = 8.2 Hz,

2H). ^{13}C NMR (101 MHz, $CDCl_3$) δ 173.15, 167.51, 137.57, 134.74,134.58, 134.02, 131.50, 130.96, 128.47, 123.91, 121.14, 52.46, 33.82. HRMS Calcd for $C_{17}H_{11}BrClNO_4$ [M+H$^+$]: 407.9638; Found: 407.9635.

1.3. General Procedure for the reaction of 4-iodoanisole with different carboxylic acids and amino acids

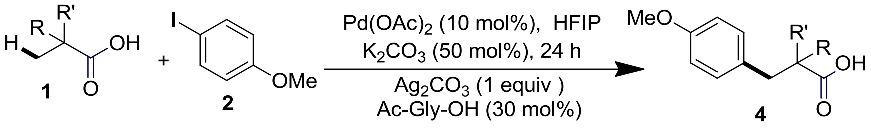

1.3.1. General Procedure for the reaction of 4-iodoanisole with 1d-1h

A mixture of **1** (0.4 mmol, 2 equiv), **4-iodoanisole** (0.2 mmol), $Pd(OAc)_2$ (4.5 mg, 10 mol%), Ag_2CO_3 (55.2 mg, 0.2 mmol, 1 equiv), K_2CO_3 (13.8 mg, 0.1 mmol, 0.5 equiv), Ac-Gly-OH (7 mg, 0.06 mmol, 0.3 equiv) in a 15 mL sealed glass vial was heated at 80 ^{o}C with vigorous stirring for 12 hours. The reaction mixture was cooled to room temperature, and diluted with ethyl acetate and filtered through celite. The filtrate was concentrated in vacuo and purified by column chromatography on silica gel (Ethyl acetate/Petroleum ether/HCOOH = 1:50:0.5% to 1:40:0.5%) to give product.

1.3.2. General Procedure for the reaction of 4-iodoanisole with 1b, 1c, 1i-1m

A mixture of **1** (0.2 mmol), **4-iodoanisole** (0.3 mmol, 1.5 equiv), $Pd(OAc)_2$ (4.5 mg, 10 mol%), Ag_2CO_3 (55.2 mg, 0.2 mmol, 1 equiv), K_2CO_3 (13.8 mg, 0.1 mmol, 0.5 equiv), Ac-Gly-OH (7 mg, 0.06 mmol, 0.3 equiv) in a 15 mL sealed glass vial was heated at 100 ^{o}C with vigorous stirring for 24 hours. The reaction mixture was cooled to room temperature, and diluted with ethyl acetate and filtered through celite. The filtrate was concentrated in vacuo and purified by column chromatography on silica gel (Ethyl acetate/Petroleum ether/HCOOH = 1:50:0.5% to 1:40:0.5%) to give product.

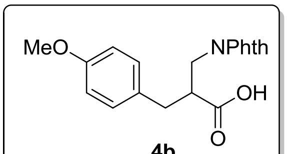

4b

White solid; Yield (71%, 48.1 mg); R_f = 0.46 (petroleum ester/ethyl acetate/HCOOH, 3/1/0.02). ^{1}H NMR (400 MHz, $CDCl_3$) δ 7.79 (dd, *J* = 5.5, 3.0 Hz, 2H), 7.69 (dd, *J* = 5.5, 3.1 Hz, 2H), 7.11 (d, *J* = 8.6 Hz, 2H), 6.75 (d, *J* = 8.6 Hz, 2H), 4.01 (dd, *J* = 13.9, 7.9 Hz, 1H), 3.86 (dd, *J* = 13.9, 6.6 Hz, 1H), 3.71 (s, 3H), 3.36 – 3.29 (m, 1H), 3.05 (dd, *J* = 14.2, 7.7 Hz, 1H), 2.80 (dd, *J* = 14.3, 7.0 Hz, 1H). ^{13}C NMR (101 MHz, $CDCl_3$) δ 177.69, 168.22, 158.38, 134.12, 132.01, 129.85, 129.79, 123.46, 114.04, 55.28, 45.28, 39.38, 35.14. HRMS Calcd for $C_{23}H_{30}N_2NaO_2$ [M+Na$^+$]: 389.2205; Found: 389.2207.

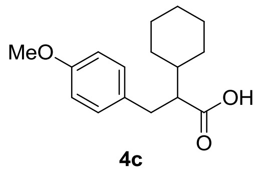

4c

Yellow oil; Yield (75%, 37.7 mg); R_f = 0.54 (petroleum ester/ethyl acetate/HCOOH, 10/1/0.02). ^{1}H NMR (400 MHz, $CDCl_3$) δ 7.08 (d, *J* = 8.6 Hz, 2H), 6.79 (d, *J* = 8.6 Hz, 2H), 3.77 (s, 3H), 2.84 – 2.75 (m, 2H), 2.48 – 2.43 (m, 1H), 1.86 (d, *J* = 12.7 Hz, 1H), 1.78 – 1.59 (m, 5H), 1.30 – 1.08 (m, 6H). ^{13}C NMR (101 MHz, $CDCl_3$) δ 180.46, 180.40, 158.17, 131.85, 129.87, 113.93, 55.32, 54.24, 40.22, 34.54, 30.88, 30.75, 26.48, 26.45, 26.43. HRMS data for the desired product were in agreement with the previously reported literature data[6].

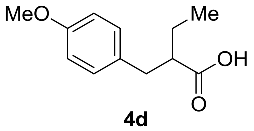

4d

Yellow oil; Yield (68%, 28.3 mg); R_f = 0.48 (petroleum ester/ethyl acetate/HCOOH, 10/1/0.02). ^{1}H NMR (400 MHz, $CDCl_3$) δ 7.10 (d, *J* = 8.5 Hz, 2H), 6.82 (d, *J* = 8.5 Hz, 2H), 3.78 (s, 3H), 2.91 (dd, *J* = 13.8, 8.0 Hz, 1H), 2.70 (dd, *J* = 13.8, 6.9 Hz, 1H), 2.60 – 2.53 (m, 1H), 1.69 – 1.55 (m, 2H), 0.95 (t, *J* = 7.4 Hz, 3H). ^{13}C NMR (101 MHz, $CDCl_3$) δ 180.56, 158.28, 131.34, 129.99, 113.96, 55.36, 49.12, 37.06, 24.89, 11.79. HRMS Calcd for $C_{12}H_{16}O_3$ [M+H$^+$]: 209.1178; Found: 209.1179.

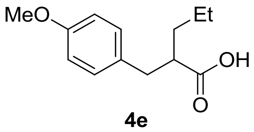

4e

Yellow oil; Yield (55%, 24.4 mg); R_f = 0.47 (petroleum ester/ethyl acetate/HCOOH, 10/1/0.02). ^{1}H NMR (400 MHz, $CDCl_3$) δ 7.10 (d, *J* = 8.5 Hz, 2H), 6.82 (d, *J* = 8.6 Hz, 2H), 3.78 (s, 3H), 2.91 (dd, *J* = 13.4, 7.7 Hz, 1H), 2.70 (dd, *J* = 13.4, 6.7 Hz, 1H), 2.65 (dd, *J* = 10.2, 5.2 Hz, 1H), 1.62 (dd, *J* = 9.2, 4.1 Hz, 1H), 1.48 (dd, *J* = 11.9, 6.5 Hz, 1H), 1.40 – 1.31 (m, 2H), 0.90 (t, *J* = 7.2 Hz, 3H). ^{13}C NMR (101 MHz, $CDCl_3$) δ 180.51, 158.29, 131.33, 129.99, 113.96, 55.36, 47.40, 37.49, 34.03, 20.62, 14.08. HRMS Calcd for $C_{13}H_{18}O_3$ [M+H$^+$]: 223.1334; Found: 223.1327.

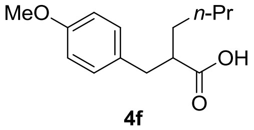

4f

Yellow oil; Yield (66%, 31.2 mg); R_f = 0.53 (petroleum ester/ethyl acetate/HCOOH, 10/1/0.02). ^{1}H NMR (400 MHz, $CDCl_3$) δ 7.09 (d, *J* = 8.3 Hz, 2H), 6.82 (d, *J* = 8.2 Hz, 2H), 3.78 (d, *J* = 0.4 Hz, 3H), 2.91 (dd, *J* = 13.6, 8.0 Hz, 1H), 2.70 (dd, *J* = 13.6, 6.7 Hz, 1H), 2.63 – 2.60 (m, 1H), 1.64 – 1.59 (m, 1H), 1.52 (dd, *J* = 8.4, 4.6 Hz, 1H), 1.32 (t, *J* = 16.5 Hz, 4H), 0.88 (t, *J* = 6.4 Hz, 3H). ^{13}C

NMR (101 MHz, $CDCl_3$) δ 181.33, 158.27, 131.34, 129.98, 113.95, 55.35, 47.69, 37.44, 31.57, 29.54, 22.70, 14.04. HRMS data for the desired product were in agreement with the previously reported literature data[7].

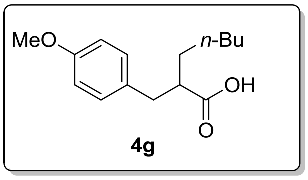

Yellow oil; Yield (61%, 30.5 mg); R_f = 0.53 (petroleum ester/ethyl acetate/HCOOH, 10/1/0.02). ^{1}H NMR (400 MHz, $CDCl_3$) δ 7.09 (d, J = 8.6 Hz, 2H), 6.82 (d, J = 8.6 Hz, 2H), 3.78 (s, 3H), 2.91 (dd, J = 13.6, 7.9 Hz, 1H), 2.70 (dd, J = 13.6, 6.7 Hz, 1H), 2.63 (dt, J = 15.1, 6.5 Hz, 1H), 1.68-1.58(m, 1H), 1.54-1.46(m, 1H), 1.38 – 1.33 (m, 2H), 1.27 (dd, J = 9.6, 6.4 Hz, 4H), 0.87 (t, J = 6.8 Hz, 3H). ^{13}C NMR (101 MHz, $CDCl_3$) δ 180.97, 158.27, 131.35, 129.98, 113.95, 55.35, 47.68, 37.46, 31.84, 31.81, 27.06, 22.61, 14.15. HRMS Calcd for $C_{15}H_{22}O_3$ [M+H$^+$]: 251.1647; Found: 251.1655.

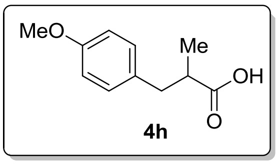

Yellow oil; Yield (80%, 31.0 mg); R_f = 0.47 (petroleum ester/ethyl acetate/HCOOH, 10/1/0.02). ^{1}H NMR (400 MHz, $CDCl_3$) δ 7.10 (d, J = 8.6 Hz, 2H), 6.83 (d, J = 8.6 Hz, 2H), 3.79 (s, 3H), 3.01 (dd, J = 13.4, 6.4 Hz, 1H), 2.72 (dd, J = 14.1, 7.1 Hz, 1H), 2.63 (dd, J = 13.4, 7.9 Hz, 1H), 1.17 (d, J = 6.9 Hz, 3H). ^{13}C NMR (101 MHz, $CDCl_3$) δ 182.33, 158.32, 131.21, 130.10, 113.96, 77.48, 77.16, 76.84, 55.37, 41.55, 38.61, 16.57. HRMS data for the desired product were in agreement with the previously reported literature data[8].

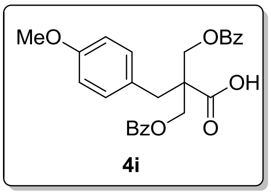

Yellow oil; Yield (60%, 53.8 mg); R_f = 0.53 (petroleum ester/ethyl acetate/HCOOH, 7/1/0.02). ^{1}H NMR (400 MHz, $CDCl_3$) δ 8.02 – 8.00 (m, 4H), 7.56 (t, J = 7.4 Hz, 2H), 7.42 (t, J = 7.7 Hz, 4H), 7.08 (d, J = 8.6 Hz, 2H), 6.75 (d, J = 8.6 Hz, 2H), 4.63 (d, J = 11.3 Hz, 2H), 4.52 (d, J = 11.3 Hz, 2H), 3.73 (s, 3H), 3.15 (s, 2H). ^{13}C NMR (101 MHz, $CDCl_3$) δ 176.36, 166.00, 158.97, 133.46, 131.06, 129.77, 129.68, 128.68, 126.54, 114.26, 63.75, 55.30, 51.53, 36.19, 0.14. HRMS Calcd for $C_{26}H_{24}O_7$ [M+H$^+$]: 449.1600; Found: 449.1609.

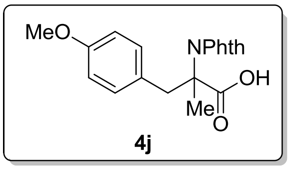

White solid; Yield (65%, 44.1 mg); R_f = 0.45 (petroleum ester/ethyl acetate/HCOOH, 3/1/0.02). ^{1}H NMR (400 MHz, $CDCl_3$) δ 7.78 (dd, *J* = 5.5, 3.0 Hz, 2H), 7.71 (dd, *J* = 5.5, 3.0 Hz, 2H), 6.97 (d, *J* = 8.6 Hz, 2H), 6.71 (d, *J* = 8.7 Hz, 2H), 3.77 (s, 1H), 3.73 (s, 3H), 3.17 (d, *J* = 14.0 Hz, 1H), 1.95 (s, 3H). ^{13}C NMR (101 MHz, $CDCl_3$) δ 177.37, 168.47, 158.81, 134.27, 131.62, 131.55, 127.46, 123.41, 113.86, 64.02, 55.27, 40.15, 21.90. HRMS Calcd for $C_{19}H_{17}NaNO_5[M+Na^+]$: 362.1004; Found: 362.1008.

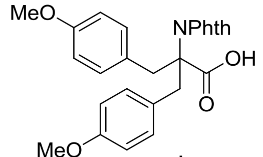

4j'

White solid; Yield (20%, 17.8 mg); R_f = 0.40 (petroleum ester/ethyl acetate/HCOOH, 3/1/0.02). ^{1}H NMR (400 MHz, $CDCl_3$) δ 7.74 (dd, *J* = 5.7, 3.0 Hz, 2H), 7.69 (dd, *J* = 5.6, 3.0 Hz, 2H), 7.19 (d, *J* = 8.6 Hz, 4H), 6.76 (d, *J* = 8.6 Hz, 4H), 3.87 (d, *J* = 14.0 Hz, 2H), 3.75 (s, 6H), 3.39 (d, *J* = 14.0 Hz, 2H). ^{13}C NMR (101 MHz, $CDCl_3$) δ 173.2, 168.4, 158.9, 134.2, 131.8, 1315, 127.4, 123.3, 113.9, 68.67, 55.3, 38.3. HRMS Calcd for $C_{26}H_{23}NaNO_6[M+H^+]$: 446.1604; Found: 446.1616.

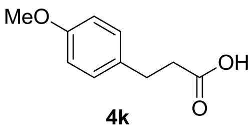

4k

White solid; Yield (55%, 19.8 mg); R_f = 0.56 (petroleum ester/ethyl acetate/HCOOH, 10/1/0.02). ^{1}H NMR (400 MHz, $CDCl_3$) δ 7.13 (d, *J* = 8.6 Hz, 2H), 6.84 (d, *J* = 8.6 Hz, 2H), 3.79 (s, 2H), 2.91 (t, *J* = 7.7 Hz, 2H), 2.65 (t, *J* = 7.7 Hz, 2H). ^{13}C NMR (101 MHz, $CDCl_3$) δ 179.00, 158.27, 132.36, 129.37, 114.10, 55.40, 35.99, 29.88. HRMS data for the desired product were in agreement with the previously reported literature data[9].

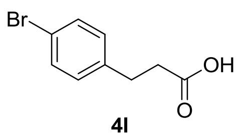

4l

White solid; Yield (39%, 17.9 mg); R_f = 0.54 (petroleum ester/ethyl acetate/HCOOH, 10/1/0.02). ^{1}H NMR (400 MHz, $CDCl_3$) δ 7.41 (d, *J* = 8.4 Hz, 2H), 7.09 (d, *J* = 8.4 Hz, 2H), 2.91 (t, *J* = 7.6 Hz, 2H), 2.66 (t, *J* = 7.6 Hz, 2H). ^{13}C NMR (101 MHz, $CDCl_3$) δ 177.85, 139.21, 131.78, 130.21, 120.37, 35.28, 30.10. HRMS data for the desired product were in agreement with the previously reported literature data[10].

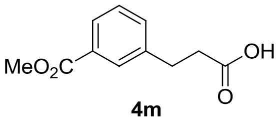

4m

White solid; Yield (35%, 14.6 mg); R_f = 0.55 (petroleum ester/ethyl acetate/HCOOH, 10/1/0.02).

^1H NMR (400 MHz, $CDCl_3$) δ 7.89 (d, *J* = 6.0 Hz, 2H), 7.43-7.25 (m, 2H), 3.91 (s, 3H), 3.01 (t, *J* = 7.7 Hz, 2H), 2.71 (t, *J* = 7.7 Hz, 2H).^{13}C NMR (101 MHz, $CDCl_3$) δ 177.72, 167.23, 140.62, 133.13, 130.58, 129.53, 128.78, 127.87, 52.30, 35.31, 30.48. HRMS Calcd for $C_{11}H_{12}O_4[M+Na^+]$: 231.0633; Found: 231.0635.

1.3.5. Ten gram scale reaction

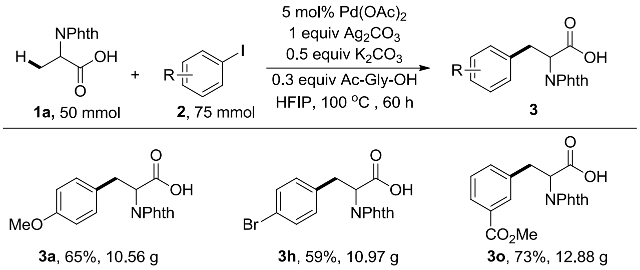

A mixture of **1a** (50 mmol, 10950 mg), **2** (75 mmol, 1.5 equiv), $Pd(OAc)_2$ (561 mg, 5 mol%), Ag_2CO_3 (13787 mg, 50 mmol, 1 equiv), K_2CO_3 (3455 mg, 25 mmol, 0.5 equiv), Ac-Gly-OH (1756mg, 15 mmol, 0.3 equiv) and 500 mL HFIP in a 1000 mL round-bottom flask which was equipped with a reflux condensing tube. A liquid sealing device was also charged to prevent solvent evaporation too fast, the reaction was refluxed at 100 °C with vigorous stirring for 60 hours. The reaction mixture was cooled to room temperature, and diluted with ethyl acetate and filtered through celite. The filtrate was concentrated in vacuo and purified by column chromatography on silica gel to give product **3a, 3h, 3o**.

2. Synthesis of biologically active compounds

2.1. ee value of 3y determined by HPLC

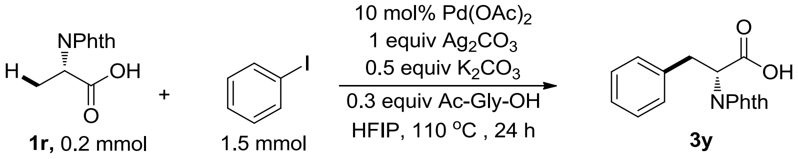

A mixture of **1r** (0.2 mmol, 43.8 mg), iodobenzene (0.3 mmol, 1.5 equiv), $Pd(OAc)_2$ (4.5 mg, 10 mol%), Ag_2CO_3 (55.2 mg, 0.2 mmol, 1 equiv), K_2CO_3 (13.8 mg, 0.1 mmol, 0.5 equiv), Ac-Gly-OH (7 mg, 0.06 mmol, 0.3 equiv) and 2 mL HFIP in a 15 mL sealed glass vial was heated at 110 °C with vigorous stirring for 24 hours. The reaction mixture was cooled to room temperature, and diluted with ethyl acetate and filtered through celite. The filtrate was concentrated in vacuo and purified by column chromatography on silica gel (Ethyl acetate/Petroleum ether/HCOOH = 1:15:0.5 % to 1:6:0.5 %) to corresponding product **3y**. Yield 66% (38.9 mg); white solid; 97.5% ee [Daicel Chiralcel OJ-H, hexanes/*i*-PrOH/TFA= 98.5/1.5/0.02, flow rate: 1.0 mL min^{-1}, λ = 254.4 nm, t (major)= 10.960, t (minor)= 18.418]. $[\alpha]_D^{25}$ = 14.03 (*c* 1.06, acetone).

2.2. Remove the protecting groups and futher transformations

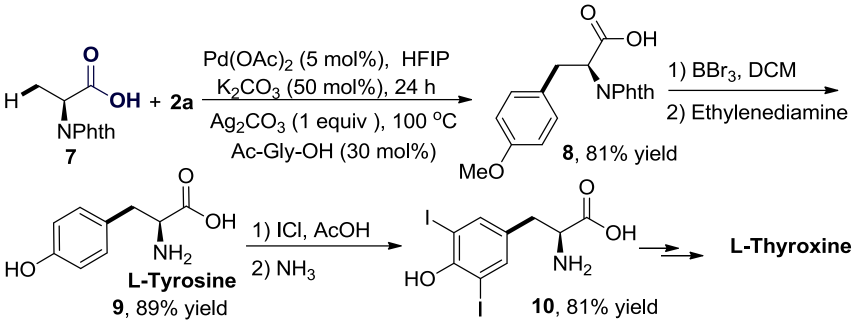

To the solution of **8** (1 mmol, 325 mg) in CH_2Cl_2 (10 mL) at 0 °C and in the atsmosphere of Ar, was added BBr_3 (1 mL, 10 equiv), which was dissolved in 5 mL CH_2Cl_2. The mixture was allowed to gradually warm to room temperature and stirred for 12 hours. The reaction was quenched with cold water in an ice bath. CH_2Cl_2 was used for extraction (5mL × 3). The combined organic phase was dried over anhydrous $MgSO_4$, filtered and concentrated to give the crude product, which was purified by flash chromatography (Ethyl acetate/Petroleum ether/HCOOH =1:2:0.5%) to afford the crude proudct as white solid. (289.2 mg, 93% yield).

To a solution of the crude product from the previous procedure (0.5 mmol, 155.5 mg) in CH_2Cl_2/EtOH (1 mL/1 mL) was added diethylamine (2.5 mmol, 150 mg, 0.16 mL). The mixture was heated to 40 °C and stirred for 4 h. After the solution was cooled to room temperature, water (1 mL) was added and CH_2Cl_2 was used for extraction (3 mL × 3). The combined organic phase was dried over anhydrous $MgSO_4$, filtered and concentrated to give the crude amino acid **9** (80.5 mg, 89% yield) as a white solid without further purification.

9 (5 mmol) and 10% hydrochloric acid solution (2 mL) was added in 25 mL three neck bottle, the mixture was heated to 60 °C and stirred. After the solid was dissoluted, chlorinated iodine (16.8 mmol, 0.8 mL), acetic acid (7.0 mL) solution was slowly dropped. Keep the reaction temperature at 60 °C. After stirring for 4 h. The reaction liquid was washed with saturated solution of sodium bisulfite. The pH value is adjusted by sodium hydroxide to 4 ~ 5 in ice bath, and a large amount of precipitation is precipitated, After filtrating, washing and drying to afford the product **10** (1749.6 mg, 81% yield)[12, 13].

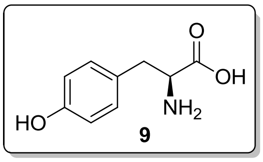

9

^{1}H NMR (600 MHz, D_2O) δ 6.60 (s, 1H), 5.77 (d, *J* = 8.5 Hz, 2H), 5.47 (d, *J* = 8.6 Hz, 2H), 2.94 – 2.92 (m, 1H), 1.85 (dd, *J* = 14.8, 5.6 Hz, 1H), 1.75 (dd, *J* = 14.8, 7.4 Hz, 1H). ^{13}C NMR (151 MHz, D_2O) δ 169.9, 153.9, 129.9, 124.4, 115.2, 114.9, 53.2, 33.6. HRMS data for the desired product was in agreement with the previously reported literature data[11].

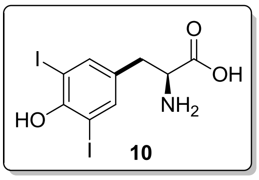

10

^1H NMR (400 MHz, DMSO) δ 7.55 (s, 2H), 4.70 (s, 2H), 3.50 – 3.26 (m, 1H), 2.96 (dd, J = 14.3, 4.3 Hz, 1H), 2.72 (dd, J = 14.3, 7.8 Hz, 1H). ^{13}C NMR (101 MHz, DMSO) δ 170.0, 156.9, 139.5, 129.9, 88.2, 55.5, 34.7. HRMS data for the desired product was in agreement with the previously reported literature data[12].

2.3. Sythesis of lopanoic acid

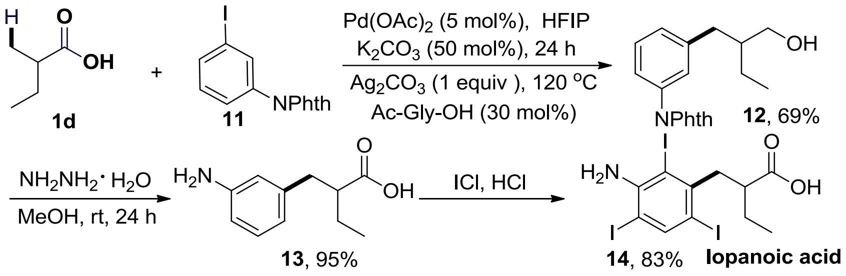

A mixture of **1d** (0.4 mmol), **11** (0.2 mmol), $Pd(OAc)_2$ (10 mol%, 4.5 mg), Ag_2CO_3 (0.2 mmol, 55.2 mg, 1 equiv), K_2CO_3 (0.1 mmol, 13.8 mg, 0.5 equiv), Ac-Gly-OH (0.06 mmol, 7.0 mg, 0.3 equiv) in a 15 mL sealed glass vial was heated at 120 °C with vigorous stirring for 24 hours. The reaction mixture was cooled to room temperature, and diluted with ethyl acetate and filtered through celite. The filtrate was concentrated in vacuo and purified by column chromatography on silica gel (Ethyl acetate/Petroleum ether/HCOOH = 1:15:0.5 % to 1:10:0.5%) to give product **12** (44.6 mg, 69% yield).

To a solution of **12** (0.5 mmol, 155.5 mg) in MeOH (5 mL) was added hydrazine hydrate (37.5 mg, 1.5 equiv). The mixture was stirred for 24 h at room temperature, and then the reaction mixture was filtrated. The filtrated was concentrated in vacuo and dissolved in DMSO (5 mL).5 mL water was added and Ethyl acetate was used for extraction (3 mL × 3). The combined organic phase was dried over anhydrous $MgSO_4$, filtered and concentrated in vacuo and purified by column chromatography on silica gel (Ethyl acetate/Petroleum ether =10:1 to 15:1) to give product **13** (91.7 mg, 95% yield).

To a solution of **13** (0.5 mmol, 96.5 mg) in HCl (145 μL, 6%) stirred and heated at 70°C, was added dropwise 241 μL iodine monochloride in 6 % HCl. At the end of the addition, heating was continued for l h then cooled to room temperature, treated with a solution of sodium bisulfite (97 μL, 20%) and extracted with dichloromethane (3×5 mL). **14** (236.6 mg, 83% yield) was purified by column chromatography on silica gel (Ethyl acetate/Petroleum ether =10:1 to 15:1).

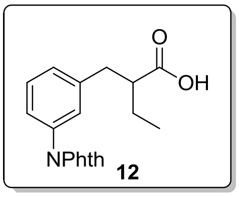

12

^1H NMR (400 MHz, $CDCl_3$) δ 7.94 (dd, J = 5.5, 3.0 Hz, 2H), 7.79 (dd, J = 5.4, 3.1 Hz, 2H), 7.42 (t, J = 8.0 Hz, 1H), 7.29 (d, J = 7.6 Hz, 2H), 7.24 (d, J = 7.7 Hz, 1H), 3.06 (dd, J = 13.8, 7.8 Hz, 1H), 2.82 (dd, J = 13.8, 7.0 Hz, 1H), 2.69 – 2.62 (m, 1H), 1.73 – 1.60 (m, 2H), 0.97 (t, J = 7.4 Hz, 3H). ^{13}C NMR (101 MHz, $CDCl_3$) δ 180.34, 167.44, 140.57, 134.55, 131.88, 131.81, 129.28, 128.85, 127.21, 124.68, 123.88, 48.65, 37.60, 24.88, 11.72. HRMS Calcd for $C_{19}H_{17}NO_4$[M+Na$^+$]: 346.1055; Found: 346.1066.

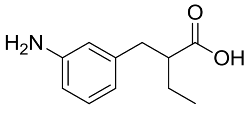

^{1}H NMR (400 MHz, $CDCl_3$) δ 7.06 (t, *J* = 7.6 Hz, 1H), 6.56 (dd, *J* = 23.3, 8.0 Hz, 3H), 4.63 (s, 2H), 2.89 (dd, *J* = 13.2, 7.3 Hz, 1H), 2.68 – 2.56 (m, 2H), 1.69 – 1.56 (m, 2H), 0.95 (t, *J* = 7.4 Hz, 3H). ^{13}C NMR (101 MHz, $CDCl_3$) δ 180.62, 146.41, 140.61, 129.47, 119.50, 115.92, 113.50, 48.72, 37.88, 24.96, 11.79. HRMS data for the desired product were in agreement with the previously reported literature data[14].

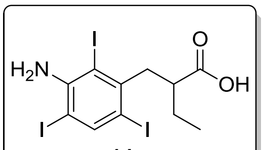

^{1}H NMR (400 MHz, $CDCl_3$) δ 8.08 (s, 1H), 4.85 (s, 2H), 3.45 (dd, *J* = 14.1, 6.9 Hz, 1H), 3.30 (dd, *J* = 14.1, 7.8 Hz, 1H), 2.83 – 2.76 (m, 1H), 1.90-1.80 (m, 1H), 1.60-1.53 (m, 1H), 0.96 (t, *J* = 7.4 Hz, 3H).^{13}C NMR (101 MHz, $CDCl_3$) δ 179.92, 147.99, 147.26, 144.46, 87.98, 84.46, 79.97, 48.16, 46.85, 24.52, 12.35. HRMS data for the desired product were in agreement with the previously reported literature data[14].

Supplementary References

[1] Mingyu Guan, Yubo Pang, Jingyu Zhang, Yingsheng Zhao, *Chem. Commun.* **2016**, *52*, 7043.

[2] D.Uraguchi, N.Kinoshita, T.Ooi, *J. Am. Chem. Soc.* **2010**, *132*, 12240.

[3] Franz Effenberger, Dieter Steegmüller, Volker Null，Thomas Ziegle, *Chemische Berichte*, **1998**,*121*, 125.

[4] J. Miao, K. Yang, M. Kurek, H. Ge, *Org. Let.* **2015**, *17*, 3738.

[5] J. R. Casimir, G. Guichard, J.-P Briand, *J. Org. Chem.* **2002**, *67*, 3764.

[6] Takashi Sugimura, Takayuki Uchida, Tomonori Misaki, Tadashi Okuyama，*J. Cata.* **2009**, *262*, 57.

[7] Shuhei Yoshida, Kenjiro Furuta, Naotaka Yamada, Eiichi Kuwano. *J. Fac. Agr. Kyushu Univ.* **2009**,*54*, 185.

[8] Zhigang Wang, Liqun Chen, Peiqiang Huang, Ying Su. *Eur. J. Med. Chem.* **2013**, *62*, 632.

[9] Zoltán Baán, Zoltán Finta, György Keglevich, István Hermecz. *Tet. Let.* **2005**, *46*, 6204.

[10] Shabashov, D., Daugulis, O. *J. Am. Chem. Soc.* **2010**, *132*, 3965.

[11] Ali Thaqi, Adam McCluskey, Janet L. Scott. *Tet. Let.* **2008**, *49*,6962.

[12] Faul, Margaret M. *Heterocycles* **2001**, *55*, 689.

[13] Grzegorz M. Salamonczyk, Vibha B. Oza, Charles J. Sih. *Tet. Let.* **1997**, *38*, 6965.

[14] Colombo, M.; De Amici, M.; De Micheli, C.; Pitre, D.; Carrea, G.; Riva, S. *Tet.* **1991**, *2*, 1021.
